# Biogeographical patterns of biomass allocation in leaves, stems, and roots in China’s forests

**DOI:** 10.1038/srep15997

**Published:** 2015-11-03

**Authors:** Hao Zhang, Kelin Wang, Xianli Xu, Tongqing Song, Yanfang Xu, Fuping Zeng

**Affiliations:** 1 Key Laboratory of Agro-Ecological Processes in Subtropical Region, Institute of Subtropical Agriculture, Chinese Academy of Sciences, Changsha, 410125, China; 2 Huanjiang Observation and Research Station for Karst Ecosystem, Chinese Academy of Sciences, Huanjiang, 547100, China; 3 State Key Laboratory for Conservation and Utilization of Subtropical Agro-bioresources, Guangxi University, Nanning, 530004, China

## Abstract

To test whether there are general patterns in biomass partitioning in relation to environmental variation when stand biomass is considered, we investigated biomass allocation in leaves, stems, and roots in China’s forests using both the national forest inventory data (2004–2008) and our field measurements (2011–2012). Distribution patterns of leaf, stem, and root biomass showed significantly different trends according to latitude, longitude, and altitude, and were positively and significantly correlated with stand age and mean annual precipitation. Trade-offs among leaves, stems, and roots varied with forest type and origin and were mainly explained by stand biomass. Based on the constraints of stand biomass, biomass allocation was also influenced by forest type, origin, stand age, stand density, mean annual temperature, precipitation, and maximum temperature in the growing season. Therefore, after stand biomass was accounted for, the residual variation in biomass allocation could be partially explained by stand characteristics and environmental factors, which may aid in quantifying carbon cycling in forest ecosystems and assessing the impacts of climate change on forest carbon dynamics in China.

The leaves of forest plants perform photosynthesis, stems (including boles and branches) provide mechanical support and a hydraulic pathway, and roots absorb nutrients and water and provide anchorage^1^,^2^. The allocation of biomass among leaves, stems, roots, and reproductive tissues can be influenced by plant size, growth environment, and species^3^,^4^. A quantitative understanding of such patterns is of fundamental importance to understanding plant ecology and evolution, and biomass patterns have implications for global climate research as well as many uses in forest practice and management^2^,^5^,^6^. Although large data sets within and across the broad spectrum of vascular plant species have been used to evaluate patterns of standing biomass^7^,^8^,^9^,^10^,^11^, theoretical or empirical assessments of these patterns remain contentious^12^,^13^,^14^,^15^,^16^,^17^,^18^. Thus, quantitative assessments of forest biomass allocation patterns remain central to forest biology and ecology.

Optimal partitioning theory (OPT), which is based on the assumption that there are trade-offs in resource allocation between leaf, stem, and root functions, has emphasized the variability in biomass allocation observed across plant species, suggesting that plants allocate biomass to the organ that acquires the most limiting resource^19^,^20^,^21^. Broad-scale biomass observations in vascular plants have supported the existence of OPT^22^,^23^,^24^, but some findings have indicated that variation in biomass allocation may be driven by differences in plant size^5^,^21^,^25^. To resolve this question, allometric biomass partitioning theory (APT), was developed to predict how plants allocate biomass in leaves, stems, and roots, based on the constraints of stand biomass^21^. Although analysis of a large collection of standing organ biomass, sampled across a broad spectrum of taxa from diverse ecological habitats, supports the relationships predicted by the model for leaf, stem, and root biomass allocation, the APT has also been questioned^6^,^13^,^14^. Therefore, it remains unclear whether general patterns in biomass partitioning exist that are linked to environmental variation after plant size is accounted for.

In China, the country with the fifth-largest forest area worldwide, multiple combinations of complex forest characteristics (e.g., age, density, forest type, and forest origin [primary, secondary, and planted forest]) as well as variable environmental factors (e.g., temperature, precipitation, soil nutrients, and topography)^26^, offer a unique opportunity to examine patterns of biomass allocation across diverse forest ecosystems. In addition, forest characteristics and environmental factors are thought to account for a large proportion of forest carbon stocks^27^,^28^,^29^. Obtaining a better understanding of the influence that these factors have on biomass allocation is fundamentally important for developing environmental policies and ecosystem carbon management practices to enhance the forest carbon sink. However, despite the progress made to date, large-scale patterns of biomass allocation in leaves, stems, and roots based on stand biomass constraints are not well quantified in China’s forests.

In this study, we investigated the biogeographic patterns of biomass allocation in leaves, stems, and roots based on a large-scale biomass survey across forest communities in China. We explored the following questions: (1) Does leaf, stem, and root biomass vary with latitude, longitude, and altitude gradients in forests? (2) Are trade-offs in biomass allocation mainly controlled by total tree biomass? (3) Are these patterns of biomass allocation also related to forest stand characteristics and large-scale environmental differences? Based on previous studies, we hypothesized that (1) leaf, stem, and root biomass would show significant biogeographic patterns, since the ability of forest plants to utilize available energy is limited by water and temperature conditions^30^,^31^,^32^; (2) the relative importance of tree size (biomass) to trade-offs in biomass allocation would be greater than that of other factors^5^,^25^; and (3) after constraints imposed by tree size were accounted for, variation in patterns of biomass allocation would be modulated by forest stand characteristics and environmental factors^14^,^25^. Exploring these important questions and hypotheses is essential for quantifying carbon cycles in forest ecosystems and assessing the impacts of climate change on forest carbon dynamics in China^6^,^10^,^33^.

## Results

### Statistics and biogeographic patterns of forest biomass allocation

**Figure 1:**
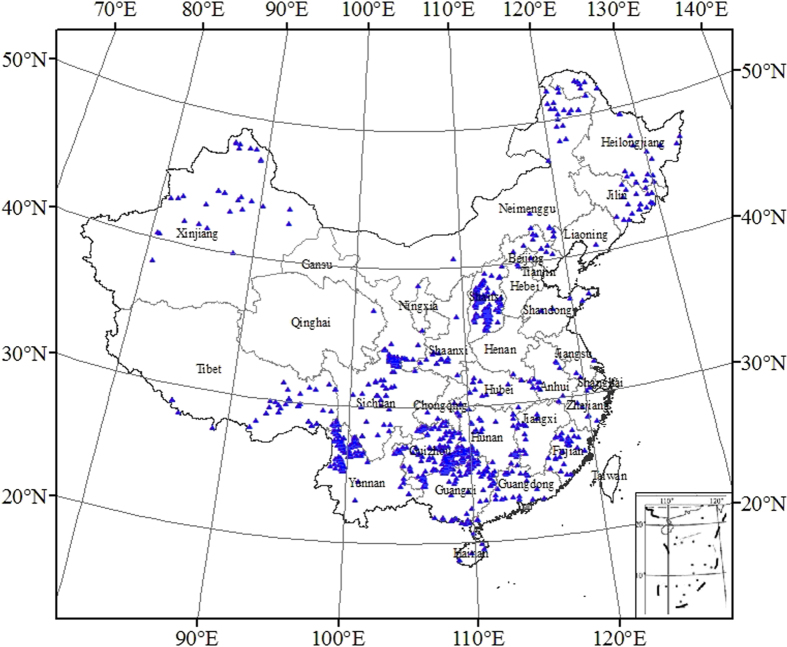
Locations of the 1,022 sampling sites across the forests of China. (The map is made by ArcGIS 10.2 software, http://www.arcgis.com/features/).

**Figure 2:**
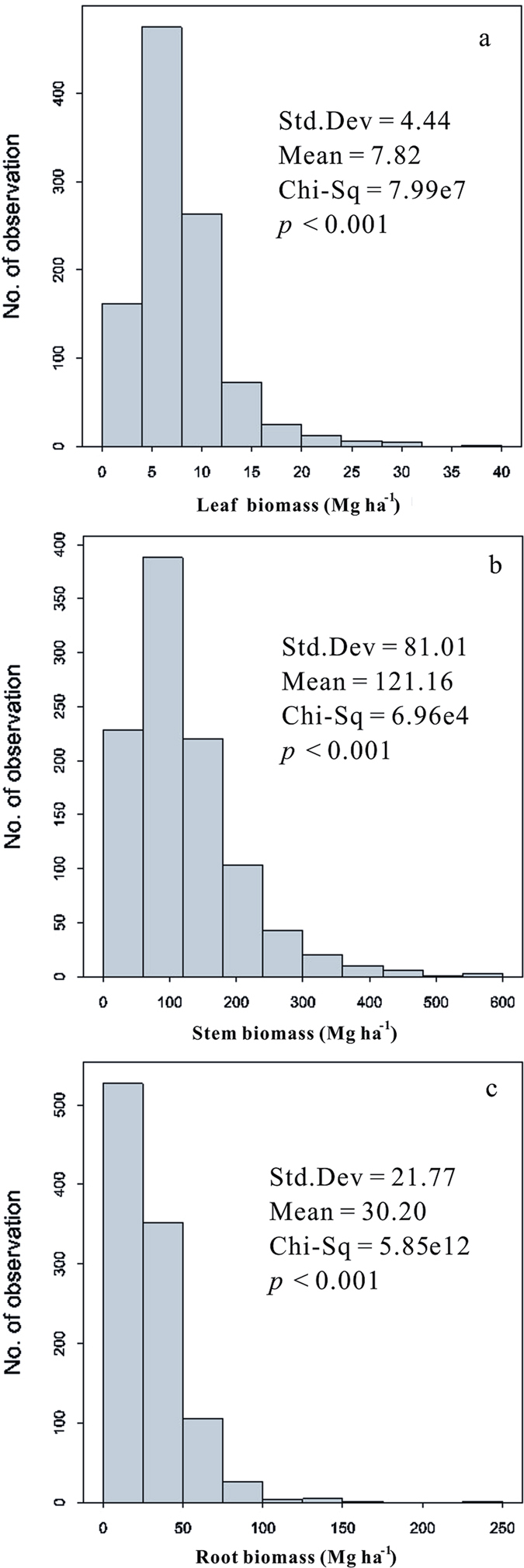
Frequency distributions of biomass in China’s forests. The mean and median values of the frequency distribution of biomass in (**a**) leaves, (**b**) stems, and (**c**) roots are presented.

Tree components exhibited large variation in biomass across sampling sites (Fig. 1), ranging from 0.42 to 38.72 Mg ha^−1^ for leaves, 12.97 to 552.86 Mg ha^−1^ for stems, and 1.48 to 232.53 Mg ha^−1^ for roots (Fig. 2), with mean values of 7.82, 121.16, and 30.20 Mg ha^−1^, respectively, and a ratio of 6.5:100:24.9. The biomass of each component varied markedly across different forest types (Table 1). However, leaf and root biomass did not differ significantly according to forest origin (Table 1).

The tree components revealed significantly different biogeographic trends (*P* < 0.01; Table 2). Leaf biomass increased significantly from west to east and from south to north, while stem and root biomass showed the opposite pattern. In addition, stem and root biomass increased significantly with increasing altitude, but leaf biomass showed no significant altitudinal trend.

### Factors influencing leaf, stem, and root biomass in China’s forests

SMR indicated that leaf, stem, and root biomass was significantly and positively correlated with AGE and MAP (Table 3), while leaf biomass was significantly and negatively correlated with TN in soil. Stem and root biomass were positively and significantly correlated with MAT and MAXT, respectively. MAP explained 13.1% of the variation in leaf biomass, while MAP and AGE combined accounted for 27.2% of this variation. However, explanation of the variation in leaf biomass was improved by only 0.9% when MAP, AGE, and TN were combined. AGE explained 33.8% and 22.0% of the variation in stem and root biomass, respectively. Together, AGE and MAT accounted for 51.2% of the variation in stem biomass, while similarly, 31.3% of the variation in root biomass was explained by the combination of AGE and MAP.

### Relationship of forest biomass allocation to stand characteristics and environmental factors

The PCA demonstrated that 75.5%, 16.6%, and 7.9% of variation in leaf, stem, and root biomass in all forests could be explained by the first, second, and third principal components (PC1, PC2, and PC3), respectively. Although the explanatory power of the PCs varied with forest type and origin, more than 64.5% of the variation in leaf, stem, and root biomass was accounted for by PC1, and more than 7.7% by PC2 (Table 4).

The PC axes contained the trade-offs between the biomass of each component, as reflected in the loadings (Table 5). For all forests, PC1 contained equal loadings for leaf, stem, and root biomass, suggesting there was variation in total stand biomass. Compared with PC1, both PC2 and PC3 loaded positively for leaf biomass and negatively for stem and root biomass, revealing a trade-off between photosynthetic and non-photosynthetic allocation (Table 5). In addition, PC1 had similar loadings for leaf, stem, and root biomass across 11 forest types and three forest origins, while loading trends in PC2 and PC3 varied with forest type and origin (Table 5).

PC1 was positively correlated with age and MAP, which demonstrated that total tree biomass increased with stand age and precipitation (Table 6). Similarly, PC2 was negatively correlated with age and positively correlated with MAP and MAXT, indicating that leaf biomass decreases with stand age and increases with precipitation and maximum temperature in the growing season, at the expense of stem and root biomass. PC3 correlated positively with density and MAT and negatively for age (Table 6), showing that leaf biomass increased with stand density and MAT at the expense of stem and root biomass. In contrast, stem and root biomass increased with age at the expense of leaf biomass. Additionally, the GLM indicated that the three PCs were closely related to forest type and origin (Table 7), which suggested that total biomass and biomass allocation were influenced by these factors.

## Discussion

In this study, the distribution of leaf, stem, and root biomass was examined, and we revealed a large variation among forests in China (Fig. 2, Table 1). Leaf biomass showed significant positive longitudinal trends while it also was characterized by significant negative latitudinal trends. Stem and root biomass revealed significant negative longitudinal trends, as well as significant positive latitudinal trends (Table 3). These results support our hypothesis that different underlying biophysical (environmental) and biological controls shape the biogeographic patterns of biomass allocation. Climatically, the north-to-south and west-to-east gradients in China reflect shifts from cold and dry to warm and moist conditions, and as might be predicted, the thermal gradient is steeper from north to south and the moisture gradient more pronounced from west to east^34^,^35^. Compared with the lack of significant altitudinal trends for leaves, both the stem and root biomass demonstrated a significant positive altitudinal trend. This phenomenon appears to be consistent with the latitudinal trends since temperature decreases as latitude and altitude increase. However, these biogeographic patterns of forest biomass allocation are also associated with geographic patterns in the structure and function of terrestrial ecosystems (e.g., stand age, forest type, biodiversity, soil development, primary production, and plant ecological traits)^36^,^37^,^38^, which also reflect responses to climate gradations and site conditions.

Although previous studies on OPT examined trends in biomass allocation and resource availability and indicated that this theory is generally correct, others have demonstrated that plant ontogeny has a strong effect on allocation patterns^39^,^40^,^41^. During shifts in plant developmental stages, more investment in support tissues is expected as plants increase in size^5^. Therefore, to understand how plants actively alter assimilation partitioning in response to changes in the environment, plant size must be considered^41^. Here, trade-offs between leaves, stems, and roots were mostly explained by PC1 (Table 4), which suggests that variation in biomass allocation is closely related to stand biomass, consistent with our hypothesis and APT.

Forest origin plays an important role in shaping biomass allocation and diversity–productivity relationships^38^,^42^. The differences in biomass allocation among natural, secondary, and planted forests may highlight the importance of the impact of forest age. After stand biomass was constrained, most secondary forests are in early successional stages, which were reflected in relatively high stem biomass, as well as low root biomass and low total forest biomass, while primary forests in mature stages had relatively low stem biomass, high root biomass, and high total forest biomass (Tables 5 and 6). Surprisingly, if stand biomass was not constrained, there was no obvious difference in trade-offs between stems and roots in primary and secondary forests (Table 1), indicating that the role of forest origin in forest biomass allocation in China may be misinterpreted if the stand biomass is not considered^32^. In addition, after stand biomass was accounted for, trade-offs between leaves, stems, and roots differed between forest types (Table 5), similar to what was previously reported^13^,^14^,^43^.

Here, we also demonstrate that leaf and root biomass increases and stem biomass decreases with stand density (Tables 4, 5, 6). Zhang *et al.* (2012) found that stem, branch, and leaf biomass varied with stand density across eight different forest types in China^44^. Some studies have also indicated that resource use is influenced by forest density. For example, due to constraints on stomatal conductance, the photosynthetic rate per unit leaf area was negatively correlated with forest density under hot, dry conditions, suggesting that stem biomass would increase in order to reduce the risk of cavitation^45^.

Our findings indicate that in all forests, leaf and root biomass increase or decrease more markedly depending on MAT or MAXT than does stem biomass (Tables 4, 5, 6). Different responses in forest biomass allocation to temperature may be partly modulated by turnover rate. Leaves and roots have higher turnover rates than stems and are more likely to contribute to biogeographic variation in biomass distribution^6^. Reich *et al.* (2014) found that forests probably allocate a smaller proportion of new biomass to foliage in cold climates since there are low turnover rates^6^. Although there is uncertainty in estimates of root biomass as a result of sampling methods and spatial heterogeneity in soil, root turnover, like leaf turnover, is probably slower in higher-latitude, or higher-altitude environments^30^,^46^. Furthermore, forests in high-latitude and high-altitude conditions are influenced by low temperature, low water availability, and low nutrient supply^47^,^48^,^49^,^50^. Therefore, both cold temperatures and low levels of soil resources contribute to the patterns of greater root biomass associated with low MAT^6^.

Our data revealed that leaf biomass was significantly and negatively correlated with TN in soil (Table 3), which is not consistent with the other studies^51^,^52^. However, after the stand biomass was considered, the leaf biomass was not significantly correlated with soil nitrogen (Table 6), suggesting the soil nitrogen was not the main factor influencing leaf biomass allocation^5^,^6^. In addition, plant response to water deficit or drought may vary with the severity of water stress^53^. Root biomass increases little under moderate water stress^54^ but increases dramatically at the expense of stems when plants are subjected to severe drought. Still, biomass allocation may not respond strongly to a drought of relatively short-duration. In this study, forest root biomass increased with decreasing MAP (Tables 4, 5, 6), which is consistent with previous research. Compared with root biomass, some studies have indicated that leaf biomass changes little with increasing drought^45^, while others found that leaf biomass increased or decreased under different drought-stress conditions^53^,^56^. Our finding that after stand biomass is considered, relative leaf biomass decreased with increasing MAP (Tables 4, 5, 6), which is consistent with some reports^53^.

Moreover, there is a significant uncertainty related to patterns of forest root biomass based on the known aboveground biomass and average root/stem ratios derived from the literature in China^10^. A quantitative understanding of the ways in which root biomass distribution is influenced by stand characteristics and environmental factors is essential for improving the accuracy of carbon inventories and simulation modeling under global climate change^57^,^58^. As described above, our results suggest that, after stand biomass is accounted for, residual variation in biomass allocation could be partially explained by stand characteristics and environmental factors, which may aid in quantifying carbon cycling in forest ecosystems and assessing impacts of climate change on forest carbon dynamics in China^7^,^8^,^9^,^10^,^11^,^59^. In addition, these relationships will help to predict the influence of climate warming, land-use change, and forest thinning on ecosystem carbon of forest vegetation. For example, our observation of higher stem biomass and lower leaf and root biomass under conditions of low stand density implies that high stand density (heavily managed) may lead to slow-growth in new stands, fast self-thinning in mature forests, and low rate of wood production. The mechanisms underlying these ecological consequences, which may involve phenotypic plasticity, tissue turnover, and resource supply, remain unclear^2^,^30^. In the future, we hope that the integration of regional inventory data, long-term monitoring data, and controlled experiments will provide answers to these important questions.

## Methods

### Large-scale forest biomass data

We used forest biomass data from 1022 sites across China, including 110 sites at which we performed field measurements from 2011 to 2012, and 912 sites from the national forest inventory data set (2004–2008) (Fig. 1). In total, 1022 observations of each tree component (leaf, stem, root, and whole tree) were included in the dataset. In detail, three replicate 20 × 50 m plots were established at each sampling site in our field survey and the national forest inventory. Each plot was divided into ten 10 × 10 m quadrats, with the height and diameter at breast height (DBH) of each tree, and the total number of trees in each plot recorded.

Based on height and DBH, five to seven trees of different diameter classes were selected from each species for measurement of tree components biomass. For tree root biomass, coarse roots of the selected trees were dug up, washed, separated by hand into size classes based on diameter (≤2 mm and >2 mm), and weighed to obtain a measure of fresh biomass. Total coarse biomass was estimated by adding together the weights of all root sizes. Fine roots were sampled using the soil block sampling method^30^. Root cores were taken at three random points around each sampled tree at different depths (mostly ≥50 cm). After soil was washed from the roots, fine roots (≤2 mm diameter) were separated from coarse roots (>2 mm) and dried at 65 °C until a constant weight was obtained. Representative root samples were taken to the laboratory, dried, and used to calculate the relationship between dry and fresh weight. Root biomass was then estimated using the regression models considering DBH (see Appendix Table S1 for details)^8^,^29^,^60^. Similarly, the leaf, bole, and branch biomass of the selected trees were separated and weighed, respectively. The representative fresh samples were also dried in the laboratory to calculate the relationship between dry and fresh weight. The biomass of the leaf, bole, and branch were then also estimated using the regression models that accounted for DBH and/or height^8^,^29^,^60^. Finally, the total biomass of the tree components per plot was computed based on the number of trees per plot.

Additionally, the ages of selected trees were measured by counting tree rings using the TSAP-Win computer program (F. Rinn Engineering Office and Distribution, Heidelberg). Stand age of planted forests (even-aged) was determined by the time since the stand was first created, while the ages of primary and secondary forests (uneven-aged) were determined by the mean age of all tree species^29^.

Site-related information, including site conditions (longitude, latitude, altitude), forest characteristics (stand age, stand density, forest type, and forest origin [primary, secondary, and planted forest]), soil chemistry (pH, total nitrogen [TN], and total phosphorus [TP]), and climate factors (e.g., mean annual temperature [MAT], mean annual precipitation [MAP]) were also documented in the dataset (see Appendix Data S1 for details).

### Climatic variables, soil data, and forest types

Growing season temperature and precipitation are important factors that affect large-scale patterns of biomass allocation^18^. Here, we used MAT (°C), MAP (mm), growing season length (GSL, days), maximum growing season temperature (MAXT, °C), mean growing season temperature (MT, °C), potential evapotranspiration (PET, mm), and aridity index (AI, PET/MAP), as indicators of climate variation. MAT, MAP, and PET were extracted from a global climate dataset (0.0083° × 0.0083° resolution, approximately 1 km × 1 km) obtained from http://www.worldclim.org/. Growing season length (GSL, the number of days with diurnal mean temperature >5 °C), MAXT, and MT were estimated from records (1970–1999) from 740 climate stations in China using a Kriging interpolation method^31^. Data were used in the analyses for each site at which the seven climatic variables and site conditions (latitude, longitude, and altitude) were recorded. For records that lacked detailed altitudinal data, we used topographic maps to acquire this information.

Soil pH, TN, and TP data were obtained from the second national soil survey and our field measurements. Forests in the dataset were primarily classified into eleven types: boreal/temperate *Larix* forest (BTLF), boreal/alpine Picea–Abies forest (BAPF), temperate *Pinus tabulaeformis* forest (TPTF), temperate/subtropical montane *Populus*–*Betula* deciduous forest (TSPF), subtropical montane *Pinus yunnanensis* and *Pinus khasya* forest (SPPF), subtropical *Pinus massoniana* forest (SPMF), subtropical montane *Pinus armandii*, *Pinus taiwanensis*, and *Pinus densada* forest (SMPF), subtropical *Cunninghamia lanceolata* forest (SCLF), subtropical evergreen broadleaved forest (SEBF), tropical rainforest and monsoon forest (TRMF), and desert riverside woodland (DRW).

### Statistical analysis

All data analyses were conducted with SPSS 19.0 (SPSS Inc., Chicago, IL). We used analysis of variance (ANOVA) to compare leaf, stem, and root biomass of different forest types and origins to determine the effects of forest characteristics on patterns of biomass allocation. Pearson correlation analyses were performed to determine the relationships between leaf, stem, and root biomass and site conditions (longitude, latitude, and altitude). Principal component analysis (PCA) was completed to determine trade-offs in leaf, stem, and root biomass. The PC axis with equal loading for leaf, stem, and root biomass accounted for stand biomass increases, and the axis with unequal loading encompassed the trade-offs in biomass between leaf, stem, and root biomass^21^. In other words, the PC axis with equal loading was first used to constrain the influence of stand biomass on the forest biomass allocation and then the axis with unequal loading was used to determine trade-offs in leaf, stem, and root biomass after stand biomass was constrained.

Stepwise multiple regressions (SMR) were used to identify the effects of forest stand characteristics (e.g., tree size, stand age, stand density) and environmental factors (e.g., climate and soil chemistry) on forest biomass and PC axes. In addition, general liner model (GLM) regressions were used to separate the variance explained by multiple factors into independent effects of individual factors and their interactive effects with the remaining factors^34^,^35^,^36^.

## Additional Information

**How to cite this article**: Zhang, H. *et al.* Biogeographical patterns of biomass allocation in leaves, stems, and roots in China's forests. *Sci. Rep.* **5**, 15997; doi: 10.1038/srep15997 (2015).

## Supplementary Material

Supplementary Information

Supplementary Data S1

## Figures and Tables

**Table 1:** Mean value of leaf, stem and root mass for eleven forest types and three forest origins.

Forest Category	Leaf biomass (Mg ha^−1^)	Stem biomass (Mg ha^−1^)	Root biomass (Mg ha^−1^)	Data number (n)
Forest type				
BTLF	4.75 ± 2.68 cd	113.10 ± 64.26 cd	34.05 ± 19.76c	46
BAPF	10.41 ± 5.41a	189.22 ± 86.11a	42.97 ± 19.95b	167
TPTF	5.74 ± 2.27c	49.17 ± 26.35d	13.32 ± 6.26d	154
TSPF	4.64 ± 2.17 cd	84.00 ± 34.74d	31.85 ± 14.63 cd	125
SPPF	8.73 ± 3.20ab	130.75 ± 64.86bc	14.64 ± 5.11d	54
SPMF	8.20 ± 3.33bc	115.00 ± 53.79 cd	23.63 ± 18.92d	65
SMPF	8.31 ± 2.94bc	95.33 ± 51.80d	20.65 ± 15.03d	57
SCLF	10.01 ± 4.94ab	101.22 ± 76.81 cd	24.38 ± 15.56d	98
SEBF	8.42 ± 4.36abc	153.75 ± 77.70b	38.65 ± 24.94bc	232
TRMF	10.85 ± 5.60a	198.77 ± 148.21a	66.40 ± 46.33a	15
DRW	1.15 ± 0.68d	47.15 ± 15.09d	10.10 ± 7.97d	9
Forest origin				
Primary	8.90 ± 3.15a	155.56 ± 32.93a	36.14 ± 13.92a	396
Second	7.68 ± 2.96a	90.68 ± 20.41b	21.62 ± 10.57a	130
Planted	7.63 ± 2.57a	112.76 ± 29.06b	28.04 ± 12.26a	496

Note: BTLF, Boreal/temperate *Larix* forest; BAPF, boreal/alpine *Picea–Abies* forest; TPTF, temperate *Pinus tabulaeformis* forest; TSPF, temperate/subtropical *montane Populus–Betula* deciduous forest; SPPF, subtropical *montane Pinus yunnanensis* and *P. khasya* forest; SPMF, subtropical *Pinus massoniana* forest; SMPF, subtropical *montane Pinus armandii, P. taiwanensis* and *P. densada* forest; SCLF, subtropical *Cunninghamia lanceolata* forest; SEBF, subtropical evergreen broadleaved forest; TRMF, tropical rainforest and monsoon forest; DRW, desert riverside woodland. These acronyms are the same as those used below.

**Table 2:** Pearson correlations between leaf biomass, stem biomass, root biomass and site conditions.

Site conditions	Leaf biomass(Mg ha^−1^)	Stem biomass (Mg ha^−1^)	Root biomass (Mg ha^−1^)
Longitude (E, °C)	0.102**	−0.240**	−0.148**
Latitude (N, °C)	−0.242**	0.255**	0.106**
Altitude (m)	0.025	0.213**	0.159**

Note: * and ** denote *p* < 0.05 and *p* < 0.01 res*p*ectively.

**Table 3:** Stepwise multiple regressions (SMR) between leaf, stem and root biomass with stand characters and environmental factors.

Tree components	Models	Equation	*P.*	R^2^
Leaf	1	Leaf = 0.36MAP + 4.18	0.000	0.131
	2	Leaf = 0.46MAP + 0.39AGE + 0.86	0.000	0.272
	3	Leaf = 0.45MAP + 0.41AGE − 0.10TN + 1.14	0.000	0.281
Stem	1	Stem = 0.58AGE + 51.75	0.000	0.338
	2	Stem = 0.79AGE + 0.47MAT − 34.82	0.000	0.512
	3	Stem = 0.76AGE + 0.28MAT + 0.23MAP − 53.84	0.000	0.533
Root	1	Root = 0.47AGE + 16.45	0.000	0.220
	2	Root = 0.55AGE + 0.32MAP − 1.40	0.000	0.313
	3	Root = 0.63AGE + 0.25MAP + 0.17MAXT − 16.08	0.000	0.327

Note: AGE, stand age; MAT, mean annual temperature; MAP, mean annual precipitation; MAXT, maximum temperature in growing season (°C); TN, total nitrogen (g/kg) in soil.

**Table 4:**
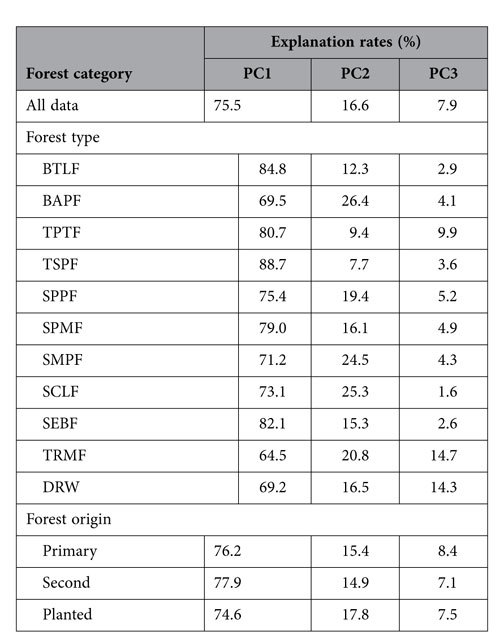
Explanation rates from principal component analyses (PCA) for leaf, stem, and root biomass across China’s forest.

**Table 5:** Eigenvector loadings for leaf, stem, and root mass from principal component analyses (PCA) for China’s forest.

Forest category	Tree part	Loading		
		PC1	PC2	PC3
All data				
	Leaf	0.58	0.84	0.72
	Stem	0.60	−0.28	−0.45
	Root	0.59	−0.47	−0.66
Forest type				
BTLF	Leaf	0.60	0.66	0.75
	Stem	0.59	−0.49	−0.34
	Root	0.57	−0.83	−0.45
BAPF	Leaf	0.52	0.72	0.46
	Stem	0.53	−0.49	−0.38
	Root	0.56	−0.76	−0.74
TPTF	Leaf	0.58	0.85	0.77
	Stem	0.61	−0.31	−0.53
	Root	0.60	−0.42	−0.68
TSPF	Leaf	0.56	0.81	0.77
	Stem	0.59	−0.25	−0.17
	Root	0.58	−0.53	−0.62
SPPF	Leaf	0.59	0.52	0.62
	Stem	0.63	−0.19	−0.25
	Root	0.61	−0.83	−0.71
SPMF	Leaf	0.58	0.84	0.66
	Stem	0.59	−0.26	−0.11
	Root	0.61	−0.48	−0.74
SMPF	Leaf	0.59	0.85	0.74
	Stem	0.62	−0.14	−0.26
	Root	0.59	−0.52	−0.62
SCLF	Leaf	0.63	0.88	0.79
	Stem	0.62	−0.44	−0.65
	Root	0.65	−0.56	−0.73
SEBF	Leaf	0.58	0.56	0.59
	Stem	0.62	−0.17	−0.26
	Root	0.59	−0.81	−0.75
TRMF	Leaf	0.57	0.84	0.79
	Stem	0.60	−0.30	−0.67
	Root	0.59	−0.45	−0.74
DRW	Leaf	0.59	0.83	0.78
	Stem	0.57	−0.20	−0.19
	Root	0.58	−0.56	−0.59
Forest origin				
Primary	Leaf	0.56	0.76	0.34
	Stem	0.60	−0.39	−0.51
	Root	0.57	−0.64	−0.79
Second	Leaf	0.57	0.83	0.71
	Stem	0.60	−0.47	−0.55
	Root	0.59	−0.29	−0.66
Planted	Leaf	0.58	0.85	0.72
	Stem	0.60	−0.35	−0.50
	Root	0.60	−0.38	−0.71

**Table 6:** Stepwise multiple regressions (SMR) between principal components with stand characters and environmental factors.

Principal components	Models	Equation	*P.*	R^2^
PC1	1	PC1 = 0.50AGE − 1.01	0.000	0.251
	2	PC1 = 0.62AGE + 0.47MAP − 2.86	0.000	0.457
	3	PC1 = 0.62AGE + 0.40MAP + 0.10GSL − 3.23	0.000	0.462
PC2	1	PC2 = −0.21AGE + 0.20	0.000	0.094
	2	PC2 = −0.17AGE + 0.16MAP − 0.09	0.000	0.178
	3	PC2 = −0.30AGE + 0.28MAP + 0.29MAXT + 0.72	0.000	0.262
	4	PC2 = −0.28AGE + 0.27MAP + 0.27MAXT + 0.10PET + 0.174	0.000	0.271
PC3	1	PC3 = 0.19DENSITY − 0.09	0.000	0.084
	2	PC3 = 0.20DENSITY + 0.16MAT + 0.01	0.000	0.173
	3	PC3 = 0.15DENSITY + 0.22MAT − 0.20AGE + 0.22	0.001	0.235
	4	PC3 = 0.13DENSITY + 0.20MAT − 0.20AGE − 0.09PET + 0.49	0.000	0.242

Note: AGE, stand age; DENSITY stand density; MAT, mean annual temperature; MAP, mean annual precipitation; GSL, growing season length; PET, potential evapotranspiration; MAXT, maximum temperature in growing season (°C).

**Table 7:** Summary of general linear models for the effects of forest origin (FO), forest types (FT), stand characters (age and density), climate (MAT, MAP, PET, MAXT, GSL), and soil parameters (pH, TN, and TP) on principal components (PC) 1, 2, and 3 across China’s forests.

Principal components	Source	SS	d.f.	F ratio	*P*
PC1	FT	39.09	10	3.80	0.0001
	FO	7.86	2	3.81	0.0271
	FT × FO	45.66	11	4.02	0.0014
	Age	93.21	1	90.58	0.0001
	MAP	72.48	1	72.41	0.0001
	FT × Age	45.52	10	4.42	0.0001
	FO × Age	13.34	2	6.48	0.0016
	FT × MAP	41.45	10	4.03	0.0001
	FO × MAP	11.65	2	5.73	0.0012
PC2	FT	4.88	10	2.84	0.0017
	FO	2.92	2	7.82	0.0148
	FT × FO	10.30	11	3.92	0.0083
	Age	1.14	1	6.60	0.0103
	MAP	1.26	1	6.48	0.0185
	MAXT	1.95	1	11.38	0.0008
	FT × Age	6.36	10	3.70	0.0028
	FO × Age	1.64	2	4.76	0.0132
	FT × MAP	6.97	10	4.06	0.0001
	FO × MAP	1.83	2	5.32	0.0051
	FT × MAXT	5.35	10	3.12	0.0006
	FO × MAXT	1.90	2	5.50	0.0039
PC3	FT	4.88	10	2.84	0.0017
	FO	2.01	2	5.88	0.0041
	FT × FO	9.27	11	3.92	0.0083
	DENSITY	1.13	1	6.60	0.0103
	MAT	1.20	1	7.20	0.0091
	Age	1.95	1	11.38	0.0008
	FT × DENSITY	6.36	10	3.70	0.0186
	FO × DENSITY	2.46	2	7.16	0.0032
	FT × MAT	6.97	10	4.06	0.0001
	FO × MAT	1.83	2	5.32	0.0051
	FT × Age	5.35	10	3.12	0.0006
	FO × Age	3.80	2	11.0	0.0003

Note: d.f., degree of freedom; MS, mean square; SS, sum of squares. Whole model (n = 1,021) for PC1, *R*^*2*^ = 0.56, P < 0.0001; for PC2, *R*^*2*^ = 0.41, P < 0.0001; for PC3, *R*^*2*^ = 0.32, P < 0.0001.
