# Supplementary material for: Biogeographical patterns of biomass allocation in leaves, stems, and roots in China’s forests
Source: Sci Rep. 2015 Nov 3;5:15997. doi: 10.1038/srep15997 (PMC4630587; doi:10.1038/srep15997)
Supplement: Supplementary Information [file srep15997-s1.pdf]

Biogeographical patterns of biomass allocation in leaves, stems and roots in China's forests

Hao Zhang[1,2,3], Kelin Wang[1,2], Xianli Xu[1,2], Tongqing Song[1,2], Yanfang Xu[1,2] & Fuping Zeng[1, 2, *]

[1]Key Laboratory of Agro-Ecological Processes in Subtropical Region, Institute of Subtropical Agriculture, Chinese Academy of Sciences, Changsha, 410125， China, [2]Huanjiang Observation and Research Station for Karst Ecosystem, Chinese Academy of Sciences, Huanjiang, 547100, China, [3]State Key Laboratory for Conservation and Utilization of Subtropical Agro-bioresources, Guangxi University, Nanning, 530004, China

[*]Correspondence and requests for materials should be addressed to F.Z. (fpzeng@isa.ac.cn)

Table S1 The root biomass regression models for trees species across China's forest

**Location (Province)**	**Tree species**	**Regression models**	R^2	**Maximum age (years)**	**Maximum DBH (cm)**
Anhui	*Pinus massoniana Lamb.*	W_R=0.023D$^{2.232}$	0.990**	84	50.4
	Cunninghamia lanceolata (Lamb.) Hook.	W_R=0.0867D$^{1.7792}$	0.956**	129	85.5
	Castanopsis eyrei (Champ.) Tutch.	W_R=0.0042D$^{3.2683}$	0.934**	75	52.9
	Castanopsis sclerophylla (Lindl.) Schott.	W_R=0.0333D$^{2.2546}$	0.999**	27	36.4
	Cyclobalanopsis glauca (Thunb.) Oerst.	W_R=0.0289D$^{2.3442}$	0.989**	79	60.0
	Pinus taiwanensis Hayata.	W_R=0.0319D$^{2.1407}$	0.983**	103	79.5
	Pinus elliottii Engelm.	W_R=0.0205D$^{2.7013}$	0.943**	28	32.5
	Populus nigra	W_R=0.0666D$^{2.0114}$	0.993**	136	98.5
	Quercus acutissima Carruth.	W_R=0.026D$^{2.4283}$	0.969**	71	47.1
	Juniperus formosana Hayata	W_R=0.040D$^{2.062}$	0.942**	124	84.5
	Maackia hupehensis	W_R=0.019D$^{2.562}$	0.921**	131	98.6
	Lindera reflexa Hemsl.	W_R=0.030D$^{2.237}$	0.899**	142	97.2
Beijing	*Larix gmelinii (Rupr.) Kuzen.*	W_R=0.0308D$^{2.2204}$	0.997**	30	31.3
	Quercus liaotungensis	W_R=0.016D$^{2.5912}$	0.995**	47	32.7
	Robinia pseudoacacia	W_R =0.3112D$^{2.3817}$	0.986**	42	38.0
	Populus	W_R= 0.0215 D$^{2.3888}$	0.996**	39	24.6
	Platycladus orientalis L.Franco	W_R= 0.0358 D$^{2.269}$	0.975**	32	22.5
	Juglans Mandshurica Maxim.	W_R= 0.0303 D$^{0.8058}$	0.936**	41	37.4
	Acer mono Maxim.	W_R= 0.067 D$^{2.3783}$	0.977**	39	23.2
	Betula davurica Pall	W_R= 0.098 D$^{2.08055}$	0.997**	35	27.7
	Tilia mongolica Maxim.	W_R= 0.0977 D$^{1.9056}$	0.991**	37	25.1

	Larix principis-rupprechtii Mayr	$W_R= 0.011\ D^{2.549}$	0.759^{**}	34	31.3
	Betula platyphylla Suk.	$W_R= 0.097\ D^{2.273}$	0.490^{*}	47	38.9
	Sorbus discolor (Maxim.) Maxim.	$W_R= 0.126\ D^{1.942}$	0.377^{*}	42	38.4
Chongqing	*Pinus massoniana Lamb.*	$W_R=0.0069D\wedge^{2.7064}$	0.983^{**}	36	37.8
	Cunninghamia lanceolata (Lamb.) Hook.	$W_R=0.0646D\wedge^{1.9265}$	0.976^{**}	49	44.5
	Cupressus funebris Endl.	$W_R=0.1201D\wedge^{1.7965}$	0.948^{**}	37	29.8
	Lithocarpus glaber	$W_R=0.0287D\wedge^{2.5826}$	0.941^{**}	34	29.0
	Pinus massoniana Lamb.	$W_R=0.0532D\wedge^{1.98}$	0.990^{**}	81	74.8
	Schima superba Gardn. et Champ.	$W_R=0.0811D\wedge^{1.93}$	0.900^{**}	67	55.4
	Cyclobalanopsis glauca (Thunb.) Oerst.	$W_R=0.133D\wedge^{1.86}$	0.992^{**}	56	47.7
	Pinus elliottii Engelm.	$W_R=0.0068D^{2.8779}$	0.980^{**}	35	21.7
	Lithocarpus glaber	$W_R=0.075D^{2.01}$	0.930^{**}	46	44.2
	Castanopsis eyrei (Champ.) Tutch.	$W_R=0.0436D^{2.1659}$	0.940^{**}	72	63.5
	Quercus variabilis Bl.	$W_R=0.0261D^{1.6906}$	0.980^{**}	25	18.8
	Cinnamomum bodinieri	$W_R=0.0334D^{2.5276}$	0.926^{**}	33	29.0
Fujian	*Cunninghamia lanceolata （Lamb.）Hook.*	$W_R=0.0165D^{2.4861}$	0.995^{**}	69	66.0
	Pinus massoniana Lamb.	$W_R=0.0095D^{2.6627}$	0.958^{**}	75	61.2
	Eucalyptus	$W_R=0.2358D^{1.6703}$	0.991^{**}	42	35.5
	Castanopsis carlesii (Hemsl.) Hay.	$W_R=0.0785D^{2.2127}$	0.999^{**}	77	61.6
	Cyclobalanopsis glauca(Thunb.) Oerst.	$W_R=0.1031D^{2.07}$	0.990^{**}	39	36.4
	(Cupressus funebris Endl	$W_R=0.0492D^{2.0518}$	0.996^{**}	26	20.9
	Schima superba Gardn. et Champ	$W_R=0.014D^{2,517}$	0.997^{**}	65	60.5
	Taxus chinensis (Pilger) Rehd	$W_R=0.057D^{2.193}$	0.926^{**}	83	74.7

	Eucalyptus L. Herit	$W_R=0.020D^{2.429}$	0.958**	79	74.9
	Castanopsis faberi	$W_R=0.023D^{2.237}$	0.989**	125	98.6
Gansu	*Pinus armandii Franch*	$W_R=0.008D^{2.8075}$	0.980**	48	47.2
	Abies fabri （Mast.）Craib	$W_R=0.0114D^{2.4378}$	0.996**	104	92.7
	Larix gmelinii （Rupr.）Kuzen	$W_R=0.0161D^{2.4605}$	0.989**	39	41.7
	Quercusalienavar．acuteserrataMaxim	$W_R=0.0712D^{1.976}$	0.978**	58	73.1
	Quercus variabilis Bl.	$W_R=0.109D^{2.131}$	0.990**	42	45.5
	Pinus tabuliformis Carrière	$W_R=0.0179D^{2.4651}$	0.980**	50	54.6
	Picea asperata Mast	$W_R=0.0307D^{2.2282}$	0.943**	79	93.3
	Picea crassifolia Kom.	$W_R=0.0113D^{2.5901}$	0.982**	142	124..5
	Betula platyphylla Suk.	$W_R=0.1142D^{1.89862}$	0.941**	87	78.9
Guangdong	*Pinus massoniana Lamb.*	$W_R=0.0375D^{2.2302}$	0.982**	72	65.2
	Eucalyptus robusta	$W_R=0.0101D^{2.6365}$	0.967**	6	17.4
	Acacia mangium	$W_R=0.0049D^{2.9697}$	0.990**	42	37.3
	Rhodoleia championii	$W_R= 0.031D^{2.2582}$	0.977**	15	12.8
	Schima superba Gardn. Champ.	$W_R= 0.031D^{2.2582}$	0.990**	83	72.4
	Michelia macclurei	$W_R= 0.0135D^{2.7935}$	0.986**	112	100
	Liquidambar formosana	$W_R= 0.0256D^{2.6166}$	0.970**	75	63.6
	Cunninghamia lanceolata (Lamb.)Hook.	$W_R= 0.3366D^{1.1953}$	0.889**	69	59.0
	Pinus elliottii Engelm.	$W_R= 0.04D^{2.7599}$	0.934**	35	30.5
	Casuarina equisetifolia Forst.	$W_R= 0.0325D^{2.339}$	0.912**	25	20.7
	Taxodium ascendens Brongn.	$W_R= 0.136D^{1.755}$	0.688**	102	94.1
	Illicium verum	$W_R= 0.0067D^{2.8327}$	0.986**	72	65.1
	Cryptocarya concinna Hance	$W_R= 0.0551D^{2.0671}$	0.733**	112	94.7
Guangxi	*Ligustrum lucidum Ait.*	$W_R=0.1517D^{0.4951}$	0.840**	38	31.3

	Cunninghamia lanceolata (Lamb.) Hook	W_R=0.0135D$^{2.4536}$	0.989**	57	50.1
	Eucalyptus robusta Smith	W_R=0.0068D$^{2.8422}$	0.954**	9	27.8
	yclobalanopsis glauca (Thunb.) Oerst.	W_R=0.0407D$^{2.3376}$	0.997**	74	62.4
	Schima superba Gardn. et Champ.	W_R=0.031D$^{2.2582}$	0.977**	81	72.4
	Hevea brasiliensis	W_R=3.6544D$^{0.929}$	0.959**	42	33.0
	Illicium verum	W_R=0.0019D$^{3.141}$	0.735**	19	16.6
	Quercus fabri Hance	W_R=0.0004D$^{3.2493}$	0.936**	41	35.5
	Viscum liquidambaricolum	W_R=0.0319D$^{1.1972}$	0.535**	38	24.3
	Pinus massoniana Lamb.	W_R= 0.0267D$^{2.1664}$	0.820**	44	31.7
	Eucalyptus urophylla	W_R= 0.0079D$^{2.3737}$	0.983**	31	22.4
	Cupressus funebris Endl.	W_R= 0.019D$^{2.236}$	0.967**	39	34.7
	Alangium chinense (Lour.) Harms	W_R= 0.010D$^{2.431}$	0.880**	189	131.6
Guizhou	*Cunninghamia lanceolata (Lamb.) Hook.*	W_R=0.0657D$^{1.9827}$	0.968**	42	38.8
	Pinusmassoniana Lamb	W_R=0.0201D$^{2.1257}$	0.942**	59	51.3
	Quercus	W_R=0.0394D$^{2.3128}$	0.942**	62	53.8
	Cupressus funebris Endl.	W_R=0.1068D$^{1.7224}$	0.981**	37	27.4
	Pinus armandii Franch.	W_R=0.0022D$^{3.0647}$	0.951**	31	25.9
	Pinus yunnanensis Franch	W_R=0.0107D$^{2.6766}$	0.999**	52	45.5
	Cyclobalanopsis glaucaIllicium verum	W_R=0.044D$^{2.2466}$	0.943**	35	21.9
	Podocarpus macrophyllus (Thunb.) D. Don	W_R= 0.06451D$^{1.9145}$	0.846**	61	60.9
	Schima superba Gardn. et Champ.)	W_R= 0.00149D$^{3.0508}$	0.835**	68	65.9
Hainan	*Hevea brasiliensis (Willd. ex A.Juss.) Muell. Arg.*	W_R=0.108D$^{1.948}$	0.980**	58	47.5
	Eucalyptus robusta Smith	W_R=0.0242D$^{2.1636}$	0.925**	27	19.7
	Burseraceae	W_R=0.0163D$^{2.6352}$	0.978**	38	35.8
	Sapindaceae	W_R= 0.0201D$^{2.4925}$	0.968**	26	20.9

	Glochidion wrightii Benth.	W_R=0.0187$D^{2.5325}$	0.953**	32	29.2
Hebei	*Robinia pseudoacacia*	W_R=0.1676$D^{1.6832}$	0.992**	39	34.0
	Larix gmelinii (Rupr.) Kuzen.Hook	W_R=0.1259$D^{1.6313}$	0.992**	41	35.6
	Populus L.	W_R=0.0192$D^{2.4109}$	0.990**	30	37.1
	Pinus tabuliformis Carr.	W_R=0.0115$D^{2.5534}$	0.978**	89	73.2
	Platycladus orientalis (L.) Franco	W_R= 0.0065$D^{2.9114}$	0.856**	95	79.8
	Betula platyphylla Suk.	W_R= 0.0782$D^{1.9179}$	0.932**	57	49.7
Henan	*Robinia pseudoacacia*	W_R=1.005$D^{1.026}$	0.988**	35	21.6
	Pinus taiwanensis Hayata	W_R=0.0196$D^{2.4088}$	0.989**	51	48.1
	Larix kaempferi (Lamb.) Carr.	W_R=0.0619$D^{2.1041}$	0.994**	43	48.3
	Larix kaempferi (Lamb.) Carr.	W_R=0.040$D^{2.115}$	0.979**	122	97.0
	Platycladus orientalis (L.) Franco	W_R= 0.0294$D^{2.283}$	0.978**	81	78.5
	Quercus fabri Hance	W_R=0.1708$D^{1.7316}$	0.881**	102	97.8
Heilongjiang	*Betula platyphylla Suk.*	W_R=0.0322$D^{2.3602}$	0.962**	72	58.6
	Tilia tuan Szyszyl.	W_R=0.2658$D^{1.2911}$	0.985**	50	37.8
	Betuladahurica Pall.	W_R=0.0251$D^{2.4479}$	0.966**	151	116.5
	Pinus koraiensis Sieb. et Zucc.	W_R=0.0149$D^{2.4367}$	0.936**	189	163.0
	Larix gmelinii (Rupr.)Kuzen.	W_R=0.0039$D^{2.6786}$	0.943**	313	258.7
	Quercusmongolica Fisch. ex Ledeb.	W_R=0.0644$D^{2.3519}$	0.990**	120	108.0
	Populus davidiana	W_R=0.2492$D^{1.6088}$	0.974**	52	49.0
	Pinus sylvestris L. var. mongolica Litv.	W_R=0.0643$D^{2.0103}$	0.997**	84	72.0
	Picea koraiensis Nakai	W_R=0.0041$D^{2.7113}$	0.956**	329	268.2
	Fraxinus mandschurica Rupr.	W_R= 0.0364$D^{2.2986}$	0.981**	164	108.8
Hubei	*Cupressus funebris Endl*.	W_R= 0.0228$D^{2.6569}$	0.967**	31	24.5
	Fagus engleriana Seem.	W_R= 0.0434$D^{2.4476}$	0.990**	119	98.0

	Quercus aliena Bl. var.acuteserrata Maxim. ex Wenz.	W_R= 0.0183$D^{2.5465}$	0.993**	72	63.6
	Liquidambarformosana Hance	W_R= 0.245$D^{1.6905}$	0.995**	46	32.7
	Pinus taiwanensis Hayata	W_R=0.0214$D^{2.3284}$	0.984**	63	55.7
	Pinus henryi Mast.	W_R= 0.058$D^{2.119}$	0.898**	78	66.1
	Dalbergia hupeana	W_R= 0.039$D^{2.413}$	0.961**	91	81.0
Hunan	*unninghamia lanceolata (Lamb.) Hook.*	W_R =0.0434$D^{2.0116}$	0.981**	37	37.8
	Pinus elliottii Engelm.	W_R =0.002$D^{3.132}$	0.995**	31	26.8
	Cupressus funebris Endl.	W_R =0.024$D^{2.41}$	0.979**	43	40.5
	Cinnamomum bodinieri Levl.	W_R =0.007$D^{2.819}$	0.990**	69	60.8
	Liquidambar formosana	W_R =0.0039$D^{2.9001}$	0.995**	58	50.5
	Sassafras tzumu (Hemsl.) Hemsl.	W_R =0.0819$D^{2.0898}$	0.820**	49	41.4
	Fagaceae	W_R =0.0295$D^{2.3669}$	0.928**	45	39.8
	Pinus massoniana Lamb.	W_R =0.0079$D^{2.6849}$	0.916**	43	40.1
	Schima superba Gardn. et Champ.x	W_R =0.0671$D^{2.2193}$	0.953**	48	40.7
	Cyclobalanopsis glauca (Thunb.) Oerst.	W_R =0.1036$D^{2.0491}$	0.948**	31	28.9
	Lithocarpus glaber (Thunb.) Nakai	W_R =0.1689$D^{1.9376}$	0.840**	51	41.8
	Populus	W_R =0.3952$D^{1.5116}$	0.820**	46	42.9
	(Metasequoia glyptostroboides Hu et Cheng	W_R =0.0102$D^{2.6152}$	0.990**	44	34.1
	Sapium discolor (Champ. ex Benth.) Muell. Arg.	W_R =0.011$D^{2.822}$	0.828**	49	37.5
Jilin	*Betula platyphylla Suk.*	W_R =0.0322$D^{2.36}$	0.947**	68	57.8
	Betula dahurica Pall.	W_R =0.0565$D^{2.1311}$	0.996**	65	50.5
	Pinus koraiensis Sieb. et Zucc.	W_R = 0.0457$D^{2.3442}$	0.991**	212	152.0
	Larix gmelinii (Rupr.) Kuzen.	W_R =0.0043$D^{2.6524}$	0.974**	63	52.3

	Quercus mongolica Fisch. Ex Ledeb.	$W_R = 0.0772D^{2.2983}$	0.999^{**}	130	91.5
	Populus davidiana	$W_R = 0.2501D^{1.6022}$	0.989^{**}	84	51.7
	Pinus sylvestris L.var. mongolica Litv.	$W_R = 0.1886D^{1.6143}$	0.987^{**}	36	29.4
	Taxus cuspidata S.et Z.	$W_R = 0.0364D^{2.2529}$	0.925^{**}	69	59.2
	Ulmus pumila	$W_R = 0.0781D^{2.0255}$	0.969^{**}	121	108.4
Jiangsu	*Platycladus orientalis (L.) Franco.*	$W_R = 0.0857D^{1.721}$	0.979^{**}	68	57.1
	Pinus thunbergii Parl.	$W_R = 0.23714D^{2.009}$	0.954^{**}	45	37.3
	Cunninghamia lanceolata (Lamb.) Hook.	$W_R = 0.0984D^{1.5396}$	0.998^{**}	37	31.2
	Populus L.	$W_R = 0.0558D^{1.9226}$	0.977^{**}	38	24.3
	Pinus elliottii Engelm.	$W_R = 0.0586D^{2.5038}$	$0.988^{`**}$	37	23.5
	Robinia pseudoacacia	$W_R = 0.0228D^{2.6026}$	0.966^{**}	31	32.7
	Cupressus funebris	$W_R = 0.003D^{3.239}$	0.573^{*}	78	63.2
	Mallotus apelta (Lour.) Muell. Arg.	$W_R = 0.088D^{1.835}$	0.790^{**}	71	56.3
Jiangxi	*Cunninghamia lanceolata (Lamb.) Hook.*	$W_R = 0.1358D^{1.8017}$	0.924^{**}	58	47.8
	Pinus elliottiiEngelm.	$W_R = 0.0048D^{3.2129}$	0.978^{**}	49	33.1
	Liquidambar formosana	$W_R = 0.0371D^{2.1989}$	0.993^{**}	42	33.1
	Pinus taiwanensis Hayata	$W_R = 0.077D^{2.039}$	0.936^{**}	66	56.1
Liaoning	*Larix olgensis*	$W_R = 0.0074D^{2.6890}$	0.999^{**}	48	36.7
	Larix gmelinii (Rupr.) Kuzen.	$W_R = 0.0399*D^{2.2191}$	0.990^{**}	52	49.7
	Larix principis-rupprechtii Mayr	$W_R = 0.0038D^{2.6932}$	0.997^{**}	49	37.0
	Quercus mongolica Fisch. ex Ledeb.	$W_R = 0.4405D^{1.4368}$	0.956^{**}	41	35.3
	Pinus tabuliformis Carr.	$W_R = 0.1259D^{1.6349}$	0.994^{**}	57	42.8
	Pinus sylvestris L. var. mongolica Litv.	$W_R = 0.1441D^{1.6684}$	0.986^{**}	30	23.0

	Platycladus orientalis (L.) Franco	$W_R = 0.1036D^{1.6805}$	0.868**	37	32.0
	Betula platyphylla Suk.	$W_R = 0.3426D^{1.6164}$	0.903**	39	35.9
Neimenggu	*Larix gmelinii (Rupr.) Kuzen.*	$W_R = 0.0508D^{2.2092}$	0.973**	173	139.4
	Pinus tabuliformis Carr.	$W_R = 0.0118D^{2.5377}$	0.992**	97	83.2
	Pinus sylvestris L. var. mongolica Litv.	$W_R = 0.2705D^{1.3868}$	0.947**	162	125.4
	Betula platyphylla Suk.	$W_R = 0.0133D^{2.7138}$	0.995**	136	100.1
	Populus davidiana	$W_R = 0.0936D^{1.8224}$	0.945**	169	138.3
	Quercus L.	$W_R = 0.3362D^{1.6306}$	0.941**	132	106.5
	Ulmus pumila L.	$W_R = 0.0633D^{2.1362}$	0.823**	159	121.0
	Populus L.	$W_R = 0.0209D^{2.3677}$	0.990**	178	141.6
	Picea asperata Mast.	$W_R = 0.0411D^{2.2702}$	0.906**	155	134.0
	Tilia tuan Szyszyl.	$W_R = 0.0458D^{2.1379}$	0.912**	102	90.1
Ningxia	*Pinus tabuliformis Carr.*	$W_R = 0.0443D^{2.0159}$	0.999**	69	55.6
	Picea crassifolia Kom.	$W_R = 4.2741D^{0.3718}$	0.542*	47	31.5
	Ulmus pumila	$W_R = 1.3251D^{0.8239}$	0.805**	35	26.2
	Larix principis-rupprechtii Mayr.	$W_R = 0.453D^{1.4446}$	0.827**	41	31.2
	Pinus armandii Franch.	$W_R = 0.856D^{1.2308}$	0.691**	46	31.7
	Populus simonii Carr.	$W_R = 0.0986D^{1.8806}$	0.860**	148	128.4
	Platycladus orientalis (L.) Franco	$W_R = 0.8626D^{1.0875}$	0.988**	71	69.2
	Picea crassifolia Kom.	$W_R = 0.0443D^{2.0159}$	0.999**	67	55.6
Qinghai	*Populus davidiana*	$W_R = 0.0279D^{2.3335}$	0.990**	164	126.1
	Picea asperata Mast.	$W_R = 0.0107D^{2.5956}$	0.980**	106	88.0
	Populus L.	$W_R = 0.0272D^{2.3332}$	0.990**	41	21.0
	Platycladus orientalis (L.) Franco	$W_R = 0.0506D^{1.9861}$	0.547*	247	178.5
	Sect. Populus	$W_R = 0.0197D^{2.4689}$	0.978**	84	67.5

	Populus cathayana	W_R = 0.0385$D^{2.0629}$	0.612*	206	178.9
Shandong	*Populus L.*	W_R =0.0038$D^{2.7576}$	0.979**	59	48.8
	Pinus tabuliformis Carr.	W_R =0.0954$D^{1.916}$	0.942**	62	40.7
	Platycladus orientalis (L.) Franco	W_R =0.3184$D^{1.3557}$	0.471*	56	41.8
	Pinus thunbergii Parl.	W_R =0.018$D^{2.7546}$	0.990**	53	40.7
	Larix kaempferi (Lamb.) Carr.	W_R =0.047$D^{2.134}$	0.965**	47	31.9
	Pinus densiflora Sieb. et Zucc.	W_R = 0.0781$D^{2.0118}$	0.837**	41	36.8
	Ailanthus altissima (Mill)	W_R =0.0399$D^{2.1202}$	0.640**	45	32.1
Shanxi	*Larix principis-rupprechtii Mayr*）	W_R =1.7438$D^{0.3216}$	0.889**	64	51.1
(Taiyuan)	*Populus davidiana*	W_R =0.0134$D^{2.5412}$	0.984**	47	37.8
	Pinus tabuliformis Carr.	W_R =0.0583$D^{2.0453}$	0.988**	79	66.3
	Quercus wutaishanica Mayr	W_R =0.2416$D^{2.3582}$	0.969**	68	54.1
	Quercus variabilis Bl.	W_R =0.1022$D^{1.8786}$	0.974**	47	36.1
	Platycladus orientalis (L.) Franco	W_R =0.3184$D^{1.3557}$	0.998**	37	24.6
	Robinia pseudoacacia	W_R =0.01779$D^{2.64480}$	0.994**	41	27.8
	Larix gmelinii (Rupr.) Kuzen.	W_R =0.0785$D^{1.9313}$	0.982**	78	67.3
	Juglans mandshurica	W_R =0.031$D^{2.6978}$	0.591*	69	53.9
Shanxi	*Pinus tabuliformis Carr.*	W_R =0.0334$D^{2.2462}$	0.993**	163	133.0
(Xi’an)	*Betula albosinensis Burk.*	W_R =0.0131$D^{2.6888}$	0.960**	58	44.0
	Larix principis-rupprechtii Mayr	W_R =0.07802$D^{2.04597}$	0.990**	36	20.6
	Pinus armandii Franch.	W_R =0.0284*$D^{2.2126}$	0.956**	47	41.8
	Pinus armandii Franch.	W_R =0.0386$D^{2.1118}$	0.874**	54	49.5
	Robinia pseudoacacia	W_R =0.0178*$D^{2.645}$	0.990**	46	36.3
	Pinus henryi Mast.	W_R =0.0265$D^{2.3865}$	0.984**	58	46.6
	Quercus wutaishanica Mayr	W_R =0.14489$D^{1.7911}$	0.976**	67	58.2

	Quercus aliena var. acuteserrataMaxim.	W_R =0.1502$D^{1.732}$	0.983**	88	72.2
	Pinus tabuliformis Carr.	W_R =0.1036$D^{1.8526}$	0.769**	165	133.0
	Pinus tabuliformis Carr.	W_R =0.0574$D^{2.027}$	0.919**	192	153.0
	Platycladus orientalis (L.) Franco	W_R =0.3184$D^{1.3557}$	0.471*	29	24.6
	Pinus bungeana Zucc.ex Endl.	W_R = 0.0339$D^{2.22}$	0.986**	73	62.9
	Betula platyphylla Suk.	W_R =0.0513$D^{2.1989}$	0.930**	76	72.6
Shanghai	*Elaeocarpus decipiens Hemsl.*	W_R=0.1053$D^{\wedge 1.6796}$	0.990**	28	22.3
	Metasequoia glyptostroboides Hu et Cheng.	W_R=0.0172D^$^{2.3939}$	0.999**	49	35.3
	Cinnamomum bodinieri.	W_R=0.0354$D^{2.4069}$	0.990**	82	64.0
	Ginkgo biloba L.	W_R=0.0948$D^{1.8665}$	0.970**	31	21.7
	Ligustrum lucidum Ait.	W_R=0.1071$D^{1.6211}$	0.921**	16	12.5
	Koelreuteria bipinnata var. integrifoliola	W_R=0.0471$D^{2.3304}$	0.888**	19	14.0
	Magnolia grandiflora	W_R=0.1047$D^{1.8103}$	0.896**	28	21.3
	Taxodium ascendens Brongn.	W_R=0.5838$D^{1.4641}$	0.580*	89	76.9
	Liquidambar formosana	W_R=0.0913$D^{2.0273}$	0.960**	68	56.2
Sichuan	*Pinus massoniana Lamb.*	W_R=0.017$D^{2.4248}$	0.981**	173	141.0
	Picea asperata Mast.	W_R=0.0043$D^{2.7008}$	0.973**	149	123.4
	Pinus tabuliformis Carr.	W_R=0.0052$D^{2.7836}$	0.997**	52	45.0
	Acer truncatum	W_R=0.1819$D^{1.7216}$	0.938**	43	37.1
	Brich	W_R=0.1079$D^{1.9553}$	0.964**	71	57.5
	Abies fabri (Mast.) Craib	W_R=0.0132$D^{2.6321}$	0.974**	165	134.3
	Pinus yunnanensis Franch.	W_R=0.0925$D^{1.1953}$	0.964**	79	66.0
	Cupressus funebris Endl.	W_R=0.2299$D^{1.8478}$	0.979**	72	60.1

	Larix potaninii Batalin	W_R=0.5838$D^{1.4641}$	0.580*	93	76.9
	Alangium chinense (Lour.) Harms	W_R=0.0913$D^{2.0273}$	0.999**	78	56.2
	Cinnamomum camphora (L.) presl	W_R=0.6761$D^{1.4439}$	0.590*	81	76.9
Tianjin	*Platycladus orientalis (L.) Franco*	W_R=0.000793$D^{3.9937}$	0.880**	26	18.3
	Robinia pseudoacacia	W_R=0.1273$D^{1.8307}$	0.963**	36	23.0
	Quercus acutissima Carruth	W_R=0.018$D^{2.3703}$	0.924**	38	27.2
	Populus tomentosa	W_R=0.02577$D^{2.3767}$	0.990**	35	20.2
	Pinus tabuliformis Carr.	W_R=0.008$D^{2.6273}$	0.900**	97	79.8
	Ailanthus altissima (Mill.) Swingle	W_R= 0.0782$D^{1.9179}$	0.932**	53	49.7
	Salix matsudana	W_R= 0.0122$D^{2.6645}$	0.841**	98	75.8
Tibet	*Quercus semicarpifolia Smith*	W_R=0.2852$D^{1.4551}$	0.421*	235	166.0
	Pinus densata Mast.	W_R=0.4445$D^{1.5296}$	0.475*	89	70.2
	Populus L.	W_R=0.0095$D^{2.5964}$	0.990**	72	62.3
	Picea asperata Mast.	W_R=0.0095$D^{2.5964}$	0.990**	253	193.4
	Pinus yunnanensis Franch.	W_R=0.112$D^{2.8575}$	0.897**	175	144.9
	Abies fabri (Mast.) Craib	W_R=0.0061$D^{2.8024}$	0.996**	347	235.2
	Pinus massoniana Lamb.	W_R=0.0506$D^{1.9861}$	0.547*	264	178.5
	Sect. Populus	W_R=0.0197$D^{2.4689}$	0.978**	72	67.5
Xinjiang	*Picea schrenkiana*	W_R=0.0184$D^{2.536}$	0.990**	162	123.5
	Populus euphratica	W_R=0.0588$D^{1.9737}$	0.990**	78	69.2
	Pinus sibirica (Loud.) Mayr	W_R=0.0986$D^{1.8806}$	0.860**	152	128.4
	Populus tremula	W_R=0.8626$D^{1.0875}$	0.988**	81	69.2
	Picea obovata Ledeb.	W_R= 0.2534$D^{1.5442}$	0.854**	176	128.4
Zhejiang	*Cunninghamia lanceolata (Lamb.) Hook.*	W_R=0.0035$D^{3.0514}$	0.880**	86	64.1
	Pinus massoniana Lamb.	W_R=0.0532$D^{1.98}$	0.990**	89	74.0

	Schima superba Gardn. et Champ.	$W_R=0.0811D^{1.93}$	0.949^{**}	71	55.2
	Cyclobalanopsis glauca (Thunb.) Oerst.	$W_R=0.133D^{1.86}$	0.990^{**}	68	47.7
	Pinus elliottii Engelm.	$W_R=0.0068D^{2.8779}$	0.990^{**}	27	21.7
	Lithocarpus glaber	$W_R=0.075D^{2.01}$	0.990^{**}	58	44.2
	Castanopsis eyrei (Champ.) Tutch.	$W_R=0.0436D^{2.1659}$	0.990^{**}	79	63.5
	Castanopsis carlesii (Hemsl.) Hay.	$W_R=0.0481D^{2.506}$	0.990^{**}	46	31.3
	Alniphyllum fortunei	$W_R=0.3907D^{1.2656}$	0.996^{**}	19	13.1
	Cryptomeria fortunei Hooibrenk ex Otto et Dietr.	$W_R=0.0969D^{1.9252}$	0.965^{**}	58	41.7
	Sassafras tzumu (Hemsl.) Hemsl.	$W_R=0.0063D^{2.9634}$	0.986^{**}	39	29.5
	Cupressus funebris Endl.	$W_R=0.0305D^{2.063}$	0.695^{*}	92	73.1
	Dalbergia hupeana	$W_R=0.1525D^{1.8958}$	0.999^{**}	81	66.7
	Ilex chinensis Sims	$W_R=0.0734D^{1.831}$	0.606^{*}	87	73.1

Note: 1)W_R and D are total tree root biomass and diameter at breast height (DBH), respectively; 2) * and ** present $p<0.05$ and $p<0.01$ respectively.
