# Supplementary material for: Biogeographical patterns of biomass allocation in leaves, stems, and roots in China’s forests
Source: Sci Rep. 2015 Nov 3;5:15997. doi: 10.1038/srep15997 (PMC4630587; doi:10.1038/srep15997)
Supplement: Supplementary Data S1 [file srep15997-s2.pdf]

Biogeographical patterns of biomass allocation in leaves, stems and roots in China’s forests

Hao Zhang[1,2,3], Kelin Wang[1,2], Xianli Xu[1,2], Tongqing Song[1,2], Yanfang Xu[1,2] & Fuping Zeng[1, 2, *]

[1]Key Laboratory of Agro-Ecological Processes in Subtropical Region, Institute of Subtropical Agriculture, Chinese Academy of Sciences, Changsha, 410125，China,

[2]Huanjiang Observation and Research Station for Karst Ecosystem, Chinese Academy of Sciences, Huanjiang, 547100, China,

[3]State Key Laboratory for Conservation and Utilization of Subtropical Agro-bioresources, Guangxi University, Nanning, 530004, China

Dataset on the leaves, branches, stems and roots biomass in forest commmunity of China, coupling with associated information on geographic location, climate, stand conditions, complied by H Zhang and H. Du, Institute of Subtropical Agriculture, Chinese Academy of Sciences, 2012-2013.
The dataset consists of the biomass of 11 forest types, 1022 records across China, based on our field measurements (2011-2012) and national forest inventory data (2004-2008).

Sheet1. Original data

ID	Forest Type	Leaf (Mg/ha)	Stem (Mg/ha)	Root (Mg/ha)	MAT (°C)	MAXT (℃)	MT (℃)	GSL (days)	MAP (mm)	PET (mm)	Arid index	Lon. (E, deg.)	Lat. (N, deg.)	Alt.(m)	Soil pH	Soil TN (g/kg)	Soil TP (g/kg)	Age (yr.)	Density (trees/ha)	Forest origin
1	BTLF	6.91	180.53	53.29	1.4	23	8.9	130	732.3	944.78	1.29	129.4081	44.3078	800	4.51	24.2	13.4	114	394	Primary
2	BTLF	2.71	65.33	18.79	-4.4	25	7.3	125	523.5	616.2	1.18	124.7088	52.3052	550	4.73	23.9	12.4	111	291	Primary
3	BTLF	2.93	65.17	25.58	-3.7	25	6.9	150	487.8	649.05	1.33	126.6071	51.7075	441	4.36	25.4	12.4	75	1140	Primary
4	BTLF	4.60	110.62	24.07	0.5	29	7.5	126	642.7	734.78	1.14	131.8004	46.5087	590	4.16	25.8	12.9	108	330	Primary
5	BTLF	7.82	184.89	52.44	1.4	23	6.6	120	767.9	753.86	0.98	130.2073	44.1017	800	5.18	23.6	13.3	158	544	Primary
6	BTLF	5.41	137.66	47.90	-3.9	27	5.8	110	483.9	646.32	1.34	125.2046	51.4073	590	4.99	22.7	15.8	104	600	Primary
7	BTLF	5.20	82.32	34.16	-6.2	21	6.5	128	606.8	576.35	0.95	124.2097	52.6039	876	4.65	22.8	14.3	53	2422	Planted
8	BTLF	3.98	95.58	34.63	-0.7	25	6.8	122	371.7	708	1.90	128.4059	49.507	500	4.17	23.1	15.9	85	675	Primary
9	BTLF	10.64	208.84	21.01	2.5	27	7.9	130	853.6	760.47	0.89	128.1045	42.4011	880	4.23	23.1	14.2	177	367	Planted
10	BTLF	5.06	220.92	20.11	2.1	21	9.6	126	832.2	806.67	0.97	128.2734	42.7585	900	4.71	23.4	5.7	160	360	Primary
11	BTLF	10.56	209.81	19.56	1.9	22	7.8	128	875.3	802.36	0.92	127.3011	42.2012	1075	4.21	25.3	2.1	195	219	Planted
12	BTLF	2.27	47.64	4.68	-5.0	19	5.9	120	554.4	605.85	1.09	124.5029	52.5079	650	6.85	21.4	5.6	100	400	Primary
13	BTLF	3.03	71.07	23.81	-3.1	28	5.5	125	430.8	649.96	1.51	123.0035	50.3353	700	7.14	22.1	6.1	55	1340	Planted
14	BTLF	3.26	110.56	24.38	-4.8	27	7.3	124	531.5	599.83	1.13	120.8061	52.2045	800	6.66	23.3	3.8	171	624	Planted
15	BTLF	2.33	56.61	15.01	-3.4	27	6.8	128	438.2	614.98	1.40	121.7075	50.4084	800	7.03	25	4.2	59	2946	Planted
16	BTLF	4.69	119.15	42.86	-3.6	20	6.6	130	451.8	618.04	1.37	121.5051	50.8035	781	6.42	15.6	2.8	136	748	Primary
17	BTLF	3.66	90.36	32.51	-3.1	29	5.6	120	433.5	681.3	1.57	124.0094	50.4011	637	7.45	14.7	3.4	136	565	Primary
18	BTLF	3.87	66.95	39.71	-1.8	20	5.1	124	370.9	705.84	1.90	121.4046	48.402	820	7.21	9.1	3.8	37	7289	Planted
19	BTLF	2.06	43.31	19.67	-2.7	27	5.4	142	407.5	711.3	1.75	119.9077	47.1009	1280	6.46	14.4	6.4	39	1153	Primary
20	BTLF	3.44	65.28	34.05	-3.8	22	6.2	131	458.1	623.23	1.36	121.0014	50.9097	812	6.87	4.4	2.1	39	4367	Primary
21	BTLF	2.34	39.63	24.34	-3.2	19	5.7	131	436.1	634.13	1.45	123.2057	50.5027	676	6.89	9.8	6.8	36	6568	Primary
22	BTLF	2.94	43.84	32.10	-4.0	23	7.3	147	477.3	583.69	1.22	121.5064	51.3065	774	6.91	15.8	4.5	43	9535	Primary
23	BTLF	3.11	61.78	30.24	-3.8	28	6.5	136	457.0	621.33	1.36	122.4042	50.6017	810	7.04	15.5	6.5	47	2294	Planted
24	BTLF	3.84	73.36	37.93	-3.5	19	6.3	147	435.9	631.09	1.45	121.6091	50.0066	887	7.32	10.7	2.2	50	3679	Planted
25	BTLF	4.30	72.27	44.73	-4.2	23	6.1	130	503.0	580.63	1.15	122.2005	52.0075	660	7.12	9.3	3.2	59	8576	Planted
26	BTLF	2.25	34	24.42	-4.4	24	6.8	143	522.3	579.4	1.11	122.2014	52.3033	660	7.01	10.4	5.2	35	8645	Primary
27	BTLF	3.38	54.47	35.89	-3.7	19	7.8	144	463.4	607.72	1.31	120.8073	51.3004	737	7.54	0.73	0.34	31	8782	Primary
28	BTLF	3.77	71.98	37.30	-2.8	25	5.6	123	405.8	675.86	1.67	121.3076	49.5016	846	7.27	0.78	0.24	53	4040	Planted
29	BTLF	1.95	66.58	9.64	2.0	26	8.0	147	619.1	950.37	1.54	111.5706	37.8606	2212	6.45	1.11	0.4	48	652	Planted
30	BTLF	1.94	66.97	9.79	1.7	28	7.9	120	568.7	948.67	1.67	111.8842	38.6711	2123	7.23	0.69	0.28	54	709	Planted
31	BTLF	1.67	59.06	8.80	0.7	31	8.7	142	594.1	935.3	1.57	112.0437	38.8438	2299	6.32	0.5	0.24	43	1098	Primary
32	BTLF	2.34	79.41	11.44	0.7	28	9.3	121	587.2	921.37	1.57	112.1201	38.9589	2278	6.43	1.38	0.21	46	588	Planted
33	BTLF	3.45	99.23	14.53	-0.3	30	8.4	125	637.6	893.09	1.40	113.6442	39.166	2367	6.22	1.4	0.35	62	1145	Primary
34	BTLF	3.99	146.61	24.03	2.8	26	9.2	139	588.0	950.37	1.62	111.46	37.9173	2055	6.93	1.28	0.54	53	1607	Planted
35	BTLF	4.18	146.79	21.77	0.5	25	7.8	125	590.4	921.37	1.56	112.2061	39.0051	2300	6.43	0.44	0.44	55	2070	Planted
36	BTLF	4.25	130.03	21.18	3.9	31	9.6	132	561.9	950.37	1.69	111.5085	37.8316	1850	6.98	1.29	0.48	31	2166	Primary
37	BTLF	3.95	107.74	34.07	4.0	28	9.7	144	775.2	750.96	0.97	102.4079	30.9022	3638	6.17	1.46	0.34	193	375	Planted

38	BTLF	4.68	129.97	24.73	4.2	28	9.4	144	766.4	750.96	0.98	102.3071	30.9089	3600	5.43	1.16	0.35	164	428	Primary
39	BTLF	1.91	29.51	20.52	-2.1	30	7.9	144	668.1	994	1.49	88.1055	47.8089	1650	7.75	0.31	0.62	30	9380	Planted
40	BTLF	4.64	103.8	42.23	-0.7	26	5.5	121	683.6	1080.88	1.58	93.0009	43.8015	2100	7.78	0.13	0.68	132	1116	Primary
41	BTLF	9.13	164.3	63.93	-3.7	28	6.8	134	931.7	1000.87	1.07	86.4082	48.101	1900	7.91	0.31	0.32	71	2178	Primary
42	BTLF	7.95	194.82	66.58	-3.5	29	5.4	124	862.9	1012.96	1.17	86.9001	48.0028	1883	8.65	0.36	0.53	172	1028	Planted
43	BTLF	10.06	271.99	87.20	-2.1	31	7.5	147	672.3	1007.74	1.50	86.9058	47.7069	1700	8.42	0.31	0.21	177	1117	Planted
44	BTLF	6.82	136.88	50.40	-3.9	25	5.9	139	774.0	838.92	1.08	88.7064	47.8025	1946	8.23	0.22	0.25	107	1775	Primary
45	BTLF	11.25	283.12	92.00	-2.6	25	8.5	124	581.5	957.04	1.65	89.4045	47.004	1871	7.92	0.12	0.59	157	1054	Primary
46	BTLF	8.34	194.93	65.83	-3.4	32	6.4	145	624.7	926.89	1.48	89.3095	47.1078	1989	7.64	0.24	0.59	156	3163	Primary
47	BTLF	10.67	222.97	72.72	-0.8	31	7.8	130	515.9	1358.24	2.63	93.1094	42.8078	2298	7.53	0.15	0.67	139	1263	Primary
48	BTLF	4.27	110.01	37.75	3.2	32	12.6	142	1274.1	1000.12	0.78	99.2261	28.6211	4240	5.42	0.23	0.34	100	428	Primary
49	**BPAF**	23.02	151.28	58.72	1.0	20	7.8	108	741.1	815.53	1.10	103.5004	34.6029	3280	7.96	1.23	0.53	96	2632	Primary
50	**BPAF**	6.99	73.95	11.61	1.0	20	7.9	134	483.7	875.34	1.81	117.2078	42.4091	1536	7.32	1.57	0.3	55	1588	Planted
51	**BPAF**	7.57	96.93	21.72	0.9	18	9.5	117	647.2	734.78	1.14	131.8049	46.5017	508	5.43	2.22	0.68	77	1533	Primary
52	**BPAF**	12.39	151.94	29.71	0.4	20	9.4	108	743.5	813.2	1.09	129.4019	44.3065	1017	5.31	2.17	0.59	131	631	Primary
53	**BPAF**	9.16	100.72	18.32	-6.6	15	7.9	115	623.2	576.35	0.92	124.207	52.6089	950	5.09	2.18	0.27	80	1363	Primary
54	**BPAF**	3.86	52.04	12.58	-3.9	20	8.6	130	510.1	619.26	1.21	125.406	52.4021	410	4.64	1.98	0.63	50	1025	Planted
55	**BPAF**	5.60	75.03	18.89	-4.2	19	5.3	117	522.6	605.85	1.16	124.5053	52.5075	487	5.11	2.18	0.59	75	1583	Primary
56	**BPAF**	13.07	158.93	32.64	-4.2	19	9.3	119	519.0	616.2	1.19	124.7007	52.3041	523	5.78	1.71	0.66	100	1950	Primary
57	**BPAF**	14.22	162.12	27.02	-0.9	16	8.8	122	469.9	720.67	1.53	128.9029	47.7092	800	5.31	2.15	0.43	104	918	Primary
58	**BPAF**	9.28	101.4	16.07	1.6	15	8.1	128	1937.3	921.29	0.48	110.3043	31.0043	3300	6.32	0.65	0.25	87	1028	Primary
59	**BPAF**	9.06	100.52	15.84	1.0	18	7.6	115	1784.4	900.42	0.50	110.7077	32.0084	3260	6.1	0.74	0.43	159	570	Primary
60	**BPAF**	8.63	98.27	16.10	1.5	17	8.6	125	1811.1	815.44	0.45	110.6076	31.7003	3200	6.56	0.41	0.48	151	468	Primary
61	**BPAF**	5.74	61.56	9.91	1.9	17	9.8	114	1919.3	934.42	0.49	110.7071	31.2091	3200	6.89	0.6	0.41	125	1073	Primary
62	**BPAF**	15.31	198.29	43.35	0.6	16	5.8	111	907.1	760.47	0.84	128.1064	42.404	1286	5.34	0.55	0.28	142	868	Primary
63	**BPAF**	11.41	136.93	25.11	1.6	16	11.1	114	824.7	801.73	0.97	129.7091	43.3027	900	5.12	1.45	0.45	132	736	Primary
64	**BPAF**	7.83	62.01	27.41	0.7	15	9.7	114	381.4	872.78	2.29	117.2245	43.5189	1300	6.54	1.02	0.39	170	765	Planted
65	**BPAF**	16.64	62.32	19.32	1.6	16	10.4	113	433.4	946.82	2.18	105.9079	38.7737	2384	6.32	0.96	0.3	74	1718	Primary
66	**BPAF**	11.58	123.2	19.36	0.3	21	8.2	148	615.7	948.67	1.54	111.8341	38.7358	2415	6.54	1.13	0.2	56	2051	Planted
67	**BPAF**	7.44	79.19	12.44	-0.1	18	6.7	167	627.1	871.27	1.39	111.8845	38.7529	2475	7.94	0.74	0.65	55	1394	Planted
68	**BPAF**	7.23	77.59	12.20	0.7	21	7.5	156	598.2	871.27	1.46	111.8833	38.7722	2327	7.23	1.36	0.57	65	1035	Primary
69	**BPAF**	5.24	55.31	8.68	0.8	16	8.2	180	589.9	871.27	1.48	111.9332	38.7986	2276	6.78	1.38	0.6	56	1213	Planted
70	**BPAF**	5.19	55.21	8.67	1.0	20	6.0	160	581.7	935.3	1.61	112.0335	38.8921	2243	6.87	1	0.65	50	1239	Planted
71	**BPAF**	7.52	79.94	12.56	-0.9	16	9.0	146	660.5	818.71	1.24	113.5728	39.1223	2490	7.12	0.54	0.47	61	1361	Planted
72	**BPAF**	10.42	109.26	17.14	0.0	19	8.5	172	568.8	949.07	1.67	112.2091	39.5005	2310	7.21	1.44	0.52	57	3298	Planted
73	**BPAF**	12.69	142.73	24.48	4.1	21	13.4	163	881.0	875.09	0.99	107.9057	33.5009	2680	6.94	0.94	0.67	80	1669	Primary
74	**BPAF**	8.94	109.71	21.38	6.3	21	13.6	153	843.6	858.37	1.02	108.3089	33.3052	2250	7.01	0.69	0.36	76	656	Primary
75	**BPAF**	10.37	115.76	20.33	3.3	22	13.2	155	854.3	820.18	0.96	107.7036	33.9094	2772	7.43	1.06	0.66	125	1085	Primary
76	**BPAF**	9.27	103.38	18.97	4.0	15	10.3	270	902.1	883.55	0.98	107.5022	33.2018	2770	7.9	0.93	0.63	98	1139	Primary
77	**BPAF**	8.56	146.27	40.32	4.9	15	13.1	247	548.8	933.4	1.70	98.8026	31.2039	3550	5.05	1.9	0.92	184	520	Planted
78	**BPAF**	8.63	249.43	53.46	3.1	19	12.4	256	670.3	768.28	1.15	101.6013	31.7099	3750	6	2.32	1.14	178	422	Planted
79	**BPAF**	10.53	252.46	60.89	4.0	16	13.4	264	662.3	926.52	1.40	101.1074	31.0088	3675	5.62	2.01	0.61	165	517	Primary
80	**BPAF**	9.67	552.86	83.36	7.6	17	13.2	275	928.8	876.74	0.94	103.0096	28.8036	3075	5.24	2.34	0.74	184	467	Planted
81	**BPAF**	10.58	276.52	63.52	3.8	16	12.2	274	783.7	748.85	0.96	102.9077	32.0072	3480	5.45	2.49	1.16	166	527	Planted

82	**BPAF**	7.84	244.58	51.93	5.5	18	13.8	251	776.0	904.82	1.17	101.5057	29.0069	3633	5.15	2.06	1.14	169	359	Planted
83	**BPAF**	11.46	250.38	59.47	7.0	19	13.5	257	833.1	832.02	1.00	103.1058	31.407	2804	5.71	2	0.78	163	577	Primary
84	**BPAF**	12.32	259.67	62.62	6.1	17	13.6	254	794.8	832.87	1.05	102.8053	31.6039	3000	5.57	2.13	1.05	169	732	Planted
85	**BPAF**	9.46	324.7	64.84	3.9	16	13.9	252	753.6	793.11	1.05	102.6036	32.0001	3475	5.17	1.93	0.76	206	417	Planted
86	**BPAF**	7.31	226.79	47.41	3.4	20	11.4	241	716.8	793.66	1.11	102.2069	31.9039	3638	5.69	2.18	0.97	243	343	Planted
87	**BPAF**	17.69	341.87	92.54	4.0	22	11.3	256	723.4	793.66	1.10	102.203	31.8039	3500	5.37	2.06	1.1	119	1054	Primary
88	**BPAF**	12.24	215.02	59.60	5.2	19	11.3	261	898.5	765.58	0.85	103.8094	31.6049	3178	5.47	2.52	0.7	91	692	Primary
89	**BPAF**	10.76	201.23	51.89	5.9	19	12.0	258	799.2	1055.53	1.32	101.2004	27.9065	3722	5.24	2.49	0.63	144	669	Primary
90	**BPAF**	10.13	183.35	49.70	4.0	17	13.8	279	831.9	799.66	0.96	103.7715	33.2537	3200	5.49	1.92	0.82	122	643	Primary
91	**BPAF**	10.34	173.6	48.10	4.4	21	10.0	254	855.2	721.34	0.84	103.762	32.6063	3200	5.01	2.35	0.69	210	640	Planted
92	**BPAF**	10.64	170.14	46.62	4.0	18	12.8	252	827.7	799.66	0.97	103.7537	33.3069	3200	5.83	2.12	1.07	103	696	Primary
93	**BPAF**	6.11	134.32	34.56	3.8	19	9.1	275	820.7	765.67	0.93	103.7692	33.5299	3200	5.8	2.59	0.96	143	451	Primary
94	**BPAF**	8.74	201.51	49.54	3.6	18	10.7	262	961.4	901.09	0.94	104.9015	33.529	3200	5.84	2.56	1	208	530	Planted
95	**BPAF**	8.34	180.34	44.69	3.8	19	8.9	244	808.7	765.67	0.95	103.6778	33.5756	3195	5.97	2.42	0.72	151	494	Primary
96	**BPAF**	7.05	120.77	32.65	3.8	18	12.3	271	814.5	765.67	0.94	103.732	33.5864	3200	5.12	2.23	0.94	121	478	Primary
97	**BPAF**	10.89	178.86	49.11	2.7	21	11.7	255	769.4	790.23	1.03	103.3386	33.6019	3480	5.25	1.99	1.19	137	694	Primary
98	**BPAF**	6.10	109.95	30.14	3.7	22	10.2	274	815.9	792.08	0.97	103.7468	33.6074	3200	5.24	2.2	0.62	130	470	Primary
99	**BPAF**	7.01	139.81	36.70	3.5	20	9.5	272	814.0	792.08	0.97	103.7365	33.6231	3252	5.06	2.1	0.81	115	543	Primary
100	**BPAF**	6.12	204.23	42.30	3.7	21	10.3	268	816.5	792.08	0.97	103.765	33.6351	3200	5.6	2.28	0.82	120	260	Primary
101	**BPAF**	11.48	255.98	64.17	4.0	21	10.8	267	814.4	792.08	0.97	103.7536	33.6543	3142	5.04	2.01	0.94	118	720	Primary
102	**BPAF**	7.58	149.94	39.46	3.7	21	11.9	241	816.3	792.08	0.97	103.7725	33.6793	3204	5.86	2.54	0.76	94	569	Primary
103	**BPAF**	11.60	242.87	63.75	3.6	22	11.0	278	916.6	844.92	0.92	104.5592	33.5518	3200	5.39	2.25	0.66	143	620	Primary
104	**BPAF**	9.24	183.93	50.20	3.6	19	11.6	246	928.7	875.01	0.94	104.6653	33.5711	3190	5.16	2.29	0.82	156	628	Primary
105	**BPAF**	12.06	223.83	63.56	3.6	20	9.2	240	847.7	776.37	0.92	104.0455	33.678	3200	5.91	2.29	0.69	162	680	Primary
106	**BPAF**	6.12	153.91	37.15	4.1	21	9.5	266	871.7	814.8	0.93	104.0355	33.0178	3200	5.74	1.95	0.6	171	358	Planted
107	**BPAF**	8.74	115.66	36.14	3.8	20	9.3	243	865.3	819.38	0.95	104.1025	33.381	3200	5.21	2.29	0.61	114	707	Primary
108	**BPAF**	10.41	167.81	45.81	3.9	17	12.2	257	839.0	769.93	0.92	103.8897	33.4262	3200	5.46	2.54	1.11	134	680	Primary
109	**BPAF**	10.28	168.79	47.03	3.0	20	10.3	249	797.1	759.31	0.95	103.5346	33.4756	3421	5.2	2.53	0.82	134	669	Primary
110	**BPAF**	7.74	162.75	39.88	3.9	17	9.2	261	825.2	740.5	0.90	103.7025	33.232	3238	5.11	2.52	0.82	169	459	Planted
111	**BPAF**	8.62	209.39	49.89	4.0	17	12.0	277	879.6	814.8	0.93	104.1227	33.074	3200	5.86	2.49	0.95	170	510	Planted
112	**BPAF**	7.39	152.58	42.17	3.6	15	11.4	249	896.4	847.67	0.95	104.4199	33.6048	3200	5.43	2.58	0.7	121	492	Primary
113	**BPAF**	8.34	169.48	47.76	3.7	20	12.9	259	850.4	776.37	0.91	104.0511	33.6313	3200	5.3	1.96	0.83	121	540	Primary
114	**BPAF**	10.12	235.57	59.27	3.7	17	12.6	269	849.6	776.37	0.91	104.0529	33.6518	3200	5.07	2.55	0.63	171	577	Planted
115	**BPAF**	10.54	201.09	54.68	3.6	18	11.2	257	846.1	776.37	0.92	104.0319	33.6845	3200	5.26	2.07	1.04	169	645	Planted
116	**BPAF**	8.26	164.32	43.44	4.1	20	13.6	267	852.9	762.65	0.89	103.921	33.1731	3188	5.1	2.25	1.12	152	526	Primary
117	**BPAF**	6.32	111.64	29.30	3.9	17	9.4	254	873.3	867.37	0.99	104.1556	33.3326	3200	5.92	2.41	1.01	128	488	Primary
118	**BPAF**	8.88	139.97	40.25	2.7	17	8.2	265	775.7	756.88	0.98	103.3255	33.3832	3500	5.44	2.59	0.84	138	540	Primary
119	**BPAF**	9.47	158.8	43.25	2.6	19	10.4	269	772.4	750.83	0.97	103.3479	33.5399	3500	5.28	1.9	0.95	148	608	Primary
120	**BPAF**	10.89	182.18	50.03	2.6	17	12.0	244	767.4	776.61	1.01	103.3058	33.5589	3500	5.15	2.28	1.12	148	686	Primary
121	**BPAF**	7.78	169.49	43.50	3.5	16	9.8	241	810.9	758.97	0.94	103.6169	33.3259	3320	5.28	2.44	0.8	129	431	Primary
122	**BPAF**	11.32	194.54	53.81	3.3	16	12.8	249	794.7	758.97	0.96	103.47	33.3321	3371	5.5	2.02	1.14	131	666	Primary
123	**BPAF**	13.21	276.01	70.86	2.7	18	10.3	244	787.5	756.88	0.96	103.4196	33.3579	3500	5.3	2.26	0.94	150	690	Primary
124	**BPAF**	9.84	191.48	49.78	2.7	20	8.8	277	781.1	756.88	0.97	103.3631	33.374	3500	5.43	2.14	0.71	158	553	Primary
125	**BPAF**	9.44	161.12	44.46	2.9	22	12.0	244	797.2	758.97	0.95	103.5014	33.3857	3436	5.12	2.27	1.08	142	591	Primary

126	**BPAF**	10.51	177.49	48.77	2.7	16	11.9	265	781.5	756.88	0.97	103.3764	33.4016	3500	5.94	2.09	1.08	154	643	Primary
127	**BPAF**	9.66	176.94	47.06	2.9	16	9.7	272	794.1	759.31	0.96	103.4948	33.4322	3450	5.86	2.1	0.67	135	572	Primary
128	**BPAF**	10.16	204.49	52.04	2.9	22	10.1	279	788.2	759.31	0.96	103.4682	33.4783	3433	5.09	2.48	1.02	146	589	Primary
129	**BPAF**	11.22	153.9	46.67	2.9	18	10.0	268	797.0	759.31	0.95	103.5405	33.4801	3433	5.54	2.53	0.75	137	783	Primary
130	**BPAF**	11.78	195.88	54.91	2.6	19	7.9	246	775.6	790.23	1.02	103.3986	33.6291	3500	5.54	1.93	0.63	150	830	Primary
131	**BPAF**	9.59	203.06	52.22	3.5	16	9.8	244	808.9	765.67	0.95	103.6612	33.5317	3275	5.58	2.51	1.01	148	530	Primary
132	**BPAF**	7.39	124.38	35.55	2.4	22	9.6	261	756.6	790.26	1.04	103.305	33.8503	3500	5.76	2.26	1.16	144	549	Primary
133	**BPAF**	9.67	138.48	40.69	2.5	22	7.8	259	764.4	790.26	1.03	103.3483	33.7613	3500	5.35	1.97	0.87	137	641	Primary
134	**BPAF**	11.64	160.87	47.96	2.3	19	11.1	265	703.7	801.22	1.14	102.9341	34.2047	3500	5.75	2.28	0.98	114	915	Primary
135	**BPAF**	11.64	160.87	47.96	2.3	17	7.6	255	703.7	801.22	1.14	102.9389	34.2033	3500	5.55	2.11	0.76	114	915	Primary
136	**BPAF**	7.44	136.75	37.82	3.8	16	13.1	272	837.0	843.34	1.01	103.8259	33.2742	3243	5.42	2.12	0.91	131	473	Primary
137	**BPAF**	10.83	134.77	44.33	3.8	20	12.9	275	817.8	799.66	0.98	103.6671	33.2803	3238	5.98	2.32	1.17	92	805	Primary
138	**BPAF**	10.14	151.28	43.87	2.6	21	7.7	277	768.7	776.61	1.01	103.3072	33.52	3500	5.32	2.2	1.19	142	664	Primary
139	**BPAF**	8.82	201.63	49.04	3.7	18	11.2	250	803.7	765.67	0.95	103.6253	33.5594	3238	5.74	2.04	0.72	140	485	Primary
140	**BPAF**	10.98	202.78	53.84	2.6	20	7.7	273	769.1	776.61	1.01	103.3213	33.5717	3500	5.94	2.24	0.81	167	658	Planted
141	**BPAF**	10.95	124.73	39.87	2.3	16	10.1	258	693.9	801.22	1.15	102.8238	34.1773	3500	5.29	2.42	1.17	109	928	Primary
142	**BPAF**	10.92	173.72	48.05	2.6	16	8.9	259	763.9	774.76	1.01	103.2894	33.5811	3500	5.08	1.99	1.02	133	740	Primary
143	**BPAF**	10.87	162.91	47.55	3.0	19	12.3	245	800.4	759.31	0.95	103.5719	33.5088	3410	5.32	2.22	1.01	119	710	Primary
144	**BPAF**	26.43	289.28	97.81	2.2	20	8.1	263	1044.6	863.22	0.83	105.4018	33.5202	3500	5.82	2.07	1.05	109	1870	Primary
145	**BPAF**	11.57	225.69	59.62	2.6	19	10.6	268	796.5	759.31	0.95	103.5442	33.5219	3500	5.83	2.6	1.06	146	640	Primary
146	**BPAF**	8.17	96.15	30.71	2.2	17	9.7	260	700.8	793.68	1.13	102.9267	34.2554	3500	5.17	2.55	0.77	90	676	Primary
147	**BPAF**	8.65	139.56	39.58	2.9	17	9.5	245	794.2	759.31	0.96	103.5067	33.4505	3450	5.7	2	0.69	137	642	Primary
148	**BPAF**	8.86	239.74	54.67	4.0	19	12.9	247	837.4	826.54	0.99	103.6041	32.6091	3325	5.13	2.37	0.75	198	413	Planted
149	**BPAF**	14.24	94.09	26.08	4.9	17	10.4	255	874.4	854.16	0.98	103.5506	31.5075	3273	5.05	2.23	1.08	46	848	Primary
150	**BPAF**	17.00	99.1	41.72	7.5	18	16.8	250	847.2	990.21	1.17	102.7064	30.1072	2900	5.74	2.57	1	55	1842	Planted
151	**BPAF**	11.51	79.91	31.22	4.1	15	10.0	242	834.0	738.63	0.89	103.7379	33.0757	3207	5.63	2	0.75	48	1082	Planted
152	**BPAF**	7.69	218.12	47.00	5.8	19	15.7	278	775.8	750.96	0.97	102.3367	30.9038	3208	5.64	2.55	0.99	214	401	Planted
153	**BPAF**	10.00	372.41	73.27	3.7	21	13.3	266	612.7	791.93	1.29	100.3067	30.9013	3800	5.75	2.01	1.19	317	405	Planted
154	**BPAF**	8.81	164.64	43.68	4.4	22	9.7	279	693.8	936.13	1.35	101.0032	30.0015	3758	5.36	2.1	0.74	164	501	Primary
155	**BPAF**	10.86	216.19	56.69	7.4	15	13.8	245	853.2	907.84	1.06	103.1044	30.9048	2805	5.93	1.95	0.76	133	644	Primary
156	**BPAF**	38.72	139.47	131.33	9.0	17	16.8	207	850.0	853.31	1.00	95.8878	29.9288	2750	7.76	1.07	0.7	350	172	Planted
157	**BPAF**	7.15	438.9	44.98	6.1	20	13.5	199	800.0	995.63	1.24	97.4082	28.6085	3500	8.06	0.79	1.02	175	237	Planted
158	**BPAF**	10.04	96.9	27.76	1.9	21	10.8	183	584.4	958.62	1.64	97.1067	31.1023	3900	8.31	1.3	0.8	100	799	Primary
159	**BPAF**	5.69	118.83	23.20	1.0	18	9.3	200	526.9	821.25	1.56	94.7069	30.9045	4150	7.79	1.2	0.99	113	244	Primary
160	**BPAF**	5.24	73.76	20.12	9.5	17	16.2	205	960.0	939.75	0.98	94.9024	30.205	2620	8.42	1.46	1.14	50	313	Planted
161	**BPAF**	5.42	406.64	50.79	6.6	21	12.4	193	850.0	895.46	1.05	95.7047	29.8066	3237	7.54	1.29	0.81	152	253	Primary
162	**BPAF**	6.18	175.29	31.03	1.5	20	6.5	209	592.8	843.37	1.42	98.2089	31.5066	3900	8.17	1.15	1.04	120	250	Primary
163	**BPAF**	8.84	123.92	27.92	2.7	19	9.6	213	486.3	894.41	1.84	94.3032	31.4022	3750	7.99	0.83	0.9	100	427	Primary
164	**BPAF**	6.69	103.25	22.35	2.4	19	8.6	208	561.3	887.98	1.58	96.5018	31.2001	3800	7.82	1.12	0.75	100	314	Primary
165	**BPAF**	7.66	114.29	25.40	1.9	20	9.5	208	570.2	878.6	1.54	95.8063	30.7042	3982	7.7	1.55	1.2	110	392	Primary
166	**BPAF**	9.57	163.96	34.29	1.6	18	8.3	192	712.0	884.4	1.24	98.5057	29.6073	4180	8.13	0.81	1	150	431	Primary
167	**BPAF**	8.87	174.98	34.05	2.6	21	8.4	200	691.8	874.76	1.26	97.8052	29.6051	3999	7.74	1.12	0.74	116	451	Primary
168	**BPAF**	6.18	194.95	36.16	4.5	15	13.7	193	544.7	818.13	1.50	93.302	29.9003	3642	7.9	0.77	0.73	129	322	Primary
169	**BPAF**	4.62	201.08	32.35	4.9	17	12.8	198	596.8	981.9	1.65	94.3069	29.5063	3631	7.71	1.2	0.93	143	214	Primary

170	**BPAF**	5.04	235.37	36.67	7.0	19	15.0	194	608.5	894.56	1.47	94.103	29.2077	3274	7.64	1.43	0.84	140	268	Primary
171	**BPAF**	5.54	284.8	43.27	5.8	21	13.5	198	579.4	832.7	1.44	94.1068	29.7058	3428	8.39	1.1	0.79	149	234	Primary
172	**BPAF**	8.17	228.48	41.64	5.1	21	10.4	185	590.9	901.57	1.53	94.7097	29.8094	3540	8.35	1.25	0.61	86	475	Primary
173	**BPAF**	4.09	283.11	34.15	12.0	20	19.6	183	436.4	815.31	1.87	85.909	28.1035	2590	7.98	1.58	0.6	140	168	Primary
174	**BPAF**	3.30	215.9	29.59	10.4	18	20.2	202	369.6	865.73	2.34	85.2048	28.9041	2786	8.22	1.43	0.89	131	529	Primary
175	**BPAF**	5.60	129.36	26.17	7.2	15	13.7	180	369.6	865.73	2.34	85.2044	28.9044	3398	7.76	1.36	0.74	105	502	Primary
176	**BPAF**	3.31	218.98	31.07	8.3	18	17.0	189	447.9	1013.56	2.26	85.9048	27.9026	3350	8.31	1.42	0.89	192	125	Planted
177	**BPAF**	4.21	272.4	36.63	11.6	21	20.7	202	447.9	1013.56	2.26	85.9091	27.905	2710	7.71	1.5	0.98	170	220	Planted
178	**BPAF**	6.57	182.8	31.63	4.3	20	10.7	184	591.3	925.46	1.57	93.1087	29.0026	3835	8.08	1.04	0.96	160	275	Primary
179	**BPAF**	3.71	290.9	32.59	13.9	20	19.0	206	716.9	1008.09	1.41	96.7069	28.7043	2000	8.35	1.38	0.64	76	169	Primary
180	**BPAF**	8.67	301.32	49.86	5.7	21	15.4	183	738.6	1002.27	1.36	97.0091	28.4054	3616	8.45	0.99	1	144	344	Primary
181	**BPAF**	4.94	219.57	35.61	4.7	15	11.7	182	559.1	950.98	1.70	93.8088	29.9044	3600	8.48	1.29	0.99	125	282	Primary
182	**BPAF**	14.87	149.01	34.57	-1.1	17	7.9	188	826.7	1160.53	1.40	88.0013	44.2071	2173	7.59	0.27	0.64	128	771	Primary
183	**BPAF**	29.23	336.22	97.88	2.3	18	10.7	192	690.9	1026.93	1.49	82.208	43.4045	1870	8.52	0.37	0.56	96	642	Primary
184	**BPAF**	31.97	397.34	129.70	2.1	17	9.2	194	625.7	807.47	1.29	84.6055	43.206	1895	8.34	0.32	0.36	115	394	Primary
185	**BPAF**	19.02	204.75	53.05	-1.1	17	6.0	176	799.1	1128.25	1.41	89.1064	44.0098	2197	8.37	0.32	0.44	122	539	Primary
186	**BPAF**	24.28	250.33	60.58	-0.2	17	8.0	165	814.3	1248.77	1.53	86.2088	44.3091	2050	8.05	0.25	0.62	122	995	Primary
187	**BPAF**	12.91	123.66	26.82	-1.1	22	7.7	191	759.0	1078.9	1.42	90.3036	43.8058	2210	8.18	0.17	0.32	86	1033	Primary
188	**BPAF**	11.00	84.3	12.12	-1.3	17	4.9	182	892.9	1208.98	1.35	85.605	44.308	2240	7.82	0.3	0.35	58	3967	Planted
189	**BPAF**	14.91	149.23	37.46	-0.7	20	8.4	183	853.5	1208.98	1.42	85.6024	44.3087	2133	8.07	0.29	0.24	95	3616	Primary
190	**BPAF**	27.89	341.23	107.79	-1.1	16	7.9	188	839.1	1022.33	1.22	81.801	43.2067	2500	8.39	0.35	0.23	261	325	Planted
191	**BPAF**	20.23	213.41	54.17	-1.3	18	7.4	187	836.4	1139.46	1.36	87.704	43.9075	2285	7.87	0.36	0.62	178	782	Planted
192	**BPAF**	16.20	161.28	40.64	0.2	22	7.3	163	636.0	883.27	1.39	87.1338	43.0352	2200	8.11	0.39	0.48	108	1221	Primary
193	**BPAF**	17.32	166.5	35.98	-0.1	16	9.0	182	676.7	1160.94	1.72	88.3021	43.3034	2170	8.65	0.35	0.26	97	1181	Primary
194	**BPAF**	24.07	260.74	69.12	0.3	16	5.4	184	760.9	997.67	1.31	81.1034	43.1074	2283	7.82	0.34	0.45	116	805	Primary
195	**BPAF**	13.81	225.25	47.97	7.5	20	17.0	238	1235.1	1046.26	0.85	98.9684	27.4575	3390	5.42	2.02	1.08	90	627	Primary
196	**BPAF**	10.39	119.76	29.42	10.0	15	16.7	263	1253.5	1046.26	0.83	98.9698	27.532	2810	6.04	2.42	0.84	90	574	Primary
197	**BPAF**	20.21	259.04	60.55	6.6	19	16.1	269	1173.2	995.11	0.85	99.2092	27.534	3590	6.29	2.05	0.9	100	1027	Primary
198	**BPAF**	29.29	353.45	84.88	5.6	21	13.4	232	1209.4	971.18	0.80	99.1401	27.8298	3780	5.77	2.59	1.2	200	1554	Planted
199	**BPAF**	7.30	430.26	60.19	8.0	17	15.6	279	1200.4	971.18	0.81	99.2688	27.8245	3220	6.51	2.24	1	150	279	Primary
200	**BPAF**	9.94	323.28	53.72	5.5	17	15.0	249	1232.0	973.65	0.79	99.1558	28.0033	3790	5.1	2.26	1	175	406	Planted
201	**BPAF**	6.74	461.77	61.55	5.7	21	11.4	243	1318.6	894.28	0.68	98.96	28.1846	3720	5.46	2.41	1.12	150	256	Primary
202	**BPAF**	5.64	207.21	33.74	6.9	17	15.6	264	1464.4	925.86	0.63	98.8432	28.5396	3420	5.2	2.36	1.57	120	222	Primary
203	**BPAF**	5.26	260.14	38.52	3.8	15	11.3	254	1483.1	963.75	0.65	98.6548	28.6203	4120	6.04	2.34	1.31	150	203	Primary
204	**BPAF**	11.02	148.52	33.99	3.3	21	12.6	251	1411.5	932.19	0.66	99.0216	28.9004	4200	6.31	2.55	1.5	100	543	Primary
205	**BPAF**	10.27	220.84	42.80	6.2	16	13.6	238	1095.0	962.25	0.88	99.5091	26.9558	3720	6.47	2.07	1.29	100	434	Primary
206	**BPAF**	3.96	246.01	33.84	8.4	20	16.4	236	1055.5	962.25	0.91	99.6277	27.0217	3220	5.97	2.09	1.43	100	151	Primary
207	**BPAF**	11.32	316.27	56.27	8.3	16	17.1	277	1095.9	1005	0.92	99.446	27.1086	3240	5.42	1.92	1.21	120	459	Primary
208	**BPAF**	12.86	372.84	65.55	8.7	15	13.8	278	1067.6	1020.14	0.96	99.5702	27.1785	3120	6.76	2.13	1.32	100	519	Primary
209	**BPAF**	10.54	169.21	36.24	5.5	15	13.7	241	981.4	1082.14	1.10	100.2086	26.8095	3880	6.14	2.56	1.52	122	497	Primary
210	**BPAF**	10.22	162.63	34.95	5.4	17	15.0	242	880.1	1081.87	1.23	100.6616	27.3203	3820	6.33	2.04	1.53	120	468	Primary
211	**BPAF**	5.24	316.64	43.94	6.7	17	13.8	231	890.2	1100.7	1.24	100.6078	27.5398	3500	6.93	2.54	1.4	200	200	Planted
212	**BPAF**	8.64	83.21	25.85	4.0	20	13.5	274	876.0	952.58	1.09	100.6036	27.6789	4100	6.86	2.56	1.32	70	880	Primary
213	**BPAF**	6.50	235.62	37.77	8.1	19	17.0	249	1130.2	994.13	0.88	99.3473	27.3687	3260	5.89	2.52	1.46	160	261	Primary

214	**BPAF**	10.90	214.43	45.52	6.9	19	14.6	246	1157.6	1039.9	0.90	99.2079	27.1062	3559	5.27	2.45	1.26	161	651	Primary
215	**BPAF**	6.21	200.67	33.90	9.6	18	17.6	278	1150.9	1053.94	0.92	99.3826	26.1406	3055	6.02	2.36	1.45	105	249	Primary
216	**BPAF**	7.85	238.2	41.09	8.1	17	14.9	258	1076.5	946.87	0.88	99.7062	27.7097	3200	6.43	2.44	1.21	155	342	Primary
217	**TPTF**	5.13	49.15	10.45	13.1	21	19.7	225	1173.1	1038.38	0.89	118.0091	32.6022	400	6.78	0.67	0.49	18	2165	Second
218	**TPTF**	4.55	121.55	16.05	4.0	21	9.5	250	841.0	762.65	0.91	103.8423	33.2227	3200	4.63	0.57	0.35	51	840	Planted
219	**TPTF**	11.16	136.8	24.97	9.2	23	18.1	200	780.7	874.16	1.12	108.5062	33.3322	1670	5.67	1.34	0.43	60	985	Planted
220	**TPTF**	4.07	103.79	23.81	4.0	24	13.6	210	819.5	811.49	0.99	103.7919	33.6448	3114	5.76	1.31	0.52	40	790	Primary
221	**TPTF**	5.09	213.71	59.96	9.6	25	16.1	220	656.6	778.63	1.19	107.3028	34.0069	1520	6.34	1.39	0.28	86	301	Primary
222	**TPTF**	2.99	22.04	7.58	7.1	22	15.4	150	730.8	1027.81	1.41	112.1211	35.434	1589	6.91	1.48	0.64	36	1010	Primary
223	**TPTF**	4.36	35.25	10.42	8.7	23	17.3	187	683.5	982.27	1.44	112.023	35.59	1242	6.95	1.52	0.47	53	760	Planted
224	**TPTF**	4.70	38.02	11.25	9.1	23	14.9	158	677.5	1015.38	1.50	112.0852	35.6017	1150	7.37	1.2	0.62	55	824	Planted
225	**TPTF**	3.10	22.41	7.96	8.9	25	14.5	186	673.2	1015.38	1.51	112.102	35.6806	1180	6.6	0.45	0.4	28	1205	Second
226	**TPTF**	3.40	24.15	8.81	9.8	23	18.0	176	662.4	1045.44	1.58	112.2228	35.7203	990	7.43	0.98	0.54	24	1481	Second
227	**TPTF**	2.79	21.73	6.84	9.1	22	15.1	192	665.6	1038.78	1.56	112.1726	35.7598	1120	7.37	1.1	0.22	35	640	Primary
228	**TPTF**	1.88	12.97	4.94	9.8	21	15.3	180	659.6	1038.78	1.57	112.2829	35.7737	980	6.77	0.93	0.56	28	984	Second
229	**TPTF**	8.76	77.36	19.87	7.6	28	16.2	207	699.9	1034.84	1.48	113.1164	35.9555	1350	7.19	0.95	0.56	59	830	Planted
230	**TPTF**	6.44	49.83	15.82	8.1	23	15.3	186	655.1	1001.42	1.53	112.0675	35.9827	1300	7.15	0.77	0.61	45	1532	Primary
231	**TPTF**	2.58	21.08	6.14	8.5	20	17.2	134	657.9	1042.74	1.58	112.4093	36.0047	1200	7.46	1.41	0.36	46	423	Primary
232	**TPTF**	4.25	35.54	9.96	7.8	20	16.1	193	618.0	1003.22	1.62	111.0732	36.0814	1380	6.67	1.14	0.45	56	587	Planted
233	**TPTF**	3.90	29.19	9.80	8.9	22	15.7	206	632.5	1030.32	1.63	112.2942	36.1703	1100	7.39	1.59	0.41	47	1186	Planted
234	**TPTF**	4.67	37.84	11.15	8.1	23	15.9	179	649.8	1014.51	1.56	112.4763	36.1761	1250	7.14	0.52	0.23	38	803	Primary
235	**TPTF**	3.14	22.94	7.98	8.5	25	17.0	146	618.8	1041.49	1.68	112.1643	36.3224	1150	6.5	1.48	0.41	53	1113	Planted
236	**TPTF**	3.63	34	7.20	7.7	25	13.1	143	1021.4	1230.42	1.20	117.2039	36.404	1000	6.92	1.28	0.36	23	1710	Planted
237	**TPTF**	5.01	41.1	11.88	8.8	23	16.6	197	607.9	1038.84	1.71	112.2533	36.4212	1065	6.95	1.13	0.5	60	791	Planted
238	**TPTF**	3.41	26.86	8.29	7.5	20	13.1	138	636.0	1036.73	1.63	112.4772	36.4229	1332	7.39	1.44	0.64	33	719	Primary
239	**TPTF**	7.97	68.18	18.45	4.7	27	10.4	139	677.0	990.6	1.46	111.8718	36.4327	1900	7.03	0.51	0.52	60	946	Planted
240	**TPTF**	3.33	23.65	8.64	6.6	26	12.3	167	610.9	990.25	1.62	111.2258	36.4585	1550	6.84	1.26	0.55	29	1461	Second
241	**TPTF**	11.80	41.62	11.20	7.1	20	15.4	181	604.7	1017.27	1.68	111.3652	36.4592	1440	6.89	1.2	0.22	40	2733	Primary
242	**TPTF**	5.27	45	12.21	8.5	28	17.6	186	611.6	1030.08	1.68	112.2986	36.4509	1135	7.5	0.99	0.3	56	633	Planted
243	**TPTF**	5.42	43.95	12.95	8.4	24	17.0	140	613.8	1030.08	1.68	112.4158	36.4768	1130	7.14	1.24	0.49	56	934	Planted
244	**TPTF**	3.36	26.74	8.14	8.0	23	17.7	185	619.7	1030.08	1.66	112.4392	36.4834	1210	6.79	0.66	0.43	56	668	Planted
245	**TPTF**	2.62	19.47	6.62	7.8	21	15.6	195	623.5	1036.73	1.66	112.4545	36.4866	1250	7.32	0.46	0.66	56	840	Planted
246	**TPTF**	7.14	54.13	11.47	9.1	29	18.8	185	962.2	1119.69	1.16	118.2025	36.5071	680	7.12	0.77	0.36	27	2513	Planted
247	**TPTF**	4.69	86.78	26.05	8.9	23	16.3	162	508.3	997.8	1.96	109.407	36.5076	1153	7.08	1.58	0.53	45	767	Primary
248	**TPTF**	3.68	28.73	8.98	8.1	27	17.0	146	606.7	1038.84	1.71	112.188	36.5266	1197	6.65	1.23	0.68	45	818	Primary
249	**TPTF**	3.05	24	7.44	7.5	23	13.2	203	627.6	1036.73	1.65	112.5494	36.5286	1300	7.44	1.36	0.23	30	654	Planted
250	**TPTF**	5.34	42.4	12.94	7.8	23	14.0	132	615.1	1030.08	1.67	112.3094	36.5382	1260	7.43	0.44	0.44	50	1072	Planted
251	**TPTF**	3.13	27.37	7.16	7.3	25	16.0	161	626.9	1030.08	1.64	112.4391	36.5358	1345	6.76	0.59	0.7	36	321	Primary
252	**TPTF**	2.98	22.39	7.46	7.6	21	13.8	204	619.8	1030.08	1.66	112.4101	36.5562	1290	7.06	1.51	0.32	46	877	Primary
253	**TPTF**	7.80	61.96	18.87	6.0	24	13.7	203	611.4	990.25	1.62	111.2012	36.5812	1650	7.44	0.74	0.49	37	1546	Planted
254	**TPTF**	3.04	25.54	7.12	6.8	22	13.3	209	621.3	1026.67	1.65	112.1024	36.5842	1450	6.82	1.45	0.32	50	412	Planted
255	**TPTF**	4.13	36.81	9.32	6.3	27	14.4	132	629.0	971.47	1.54	112.0757	36.6055	1550	6.59	0.53	0.25	59	367	Planted
256	**TPTF**	3.81	27.83	9.70	8.0	22	15.3	194	606.6	1022.44	1.69	112.3457	36.6007	1207	7.26	0.97	0.59	29	1358	Planted
257	**TPTF**	5.51	41.38	13.81	6.3	26	13.1	132	600.6	978.74	1.63	111.2283	36.6393	1580	7.06	0.67	0.23	38	1634	Primary

258	TPTF	6.34	57.29	14.19	5.6	25	15.5	136	641.2	971.47	1.52	112.0748	36.6388	1680	7.19	0.88	0.38	56	514	Planted
259	TPTF	2.48	18.09	6.34	7.7	20	15.1	185	607.7	1022.44	1.68	112.3422	36.6305	1251	6.94	0.68	0.5	41	910	Primary
260	TPTF	7.27	61.4	16.94	5.0	20	10.0	186	655.4	971.47	1.48	112.1048	36.6556	1800	7.43	1.46	0.32	56	938	Planted
261	TPTF	5.64	44.88	13.63	7.9	21	16.1	165	600.4	1017.58	1.69	112.2789	36.652	1210	7.23	0.89	0.54	45	1107	Primary
262	TPTF	7.90	68.99	18.05	5.0	20	13.8	156	649.4	971.47	1.50	112.0739	36.7044	1790	6.92	1.43	0.64	56	811	Planted
263	TPTF	6.48	60.93	14.15	4.9	25	12.0	172	652.9	1017.58	1.56	112.1253	36.7056	1804	7.28	1.59	0.66	50	397	Planted
264	TPTF	3.63	30.98	6.43	10.5	20	18.8	171	913.1	1105.29	1.21	121.1066	36.7003	250	6.7	0.5	0.63	15	3533	Second
265	TPTF	8.66	57.87	23.47	6.7	28	15.8	198	610.2	971.47	1.59	112.0079	36.7023	1445	7.45	1.47	0.4	25	8506	Primary
266	TPTF	3.89	31.3	9.33	7.2	28	13.3	139	604.8	1017.58	1.68	112.2353	36.7338	1352	6.68	0.75	0.38	43	703	Primary
267	TPTF	7.76	65.84	18.06	7.1	20	16.0	196	605.5	1004.83	1.66	112.3431	36.7761	1352	7.17	0.74	0.51	71	979	Primary
268	TPTF	4.30	31.27	11.00	7.2	24	16.3	147	603.8	1004.83	1.66	112.3789	36.7774	1322	7.07	0.45	0.35	36	1601	Primary
269	TPTF	7.84	65.97	18.32	4.8	27	13.4	183	647.7	1002.14	1.55	112.1251	36.7851	1810	6.71	1.02	0.41	45	1042	Primary
270	TPTF	7.41	63.68	17.11	5.8	27	15.6	169	621.1	1002.14	1.61	112.1705	36.829	1600	6.5	0.88	0.24	43	854	Primary
271	TPTF	5.68	46.32	13.54	6.2	21	12.8	204	618.9	1004.83	1.62	112.345	36.8223	1527	7.07	0.45	0.33	44	943	Planted
272	TPTF	5.30	46.31	12.10	6.7	25	11.9	183	609.8	1004.83	1.65	112.4154	36.8398	1421	7.34	0.44	0.53	68	541	Primary
273	TPTF	8.52	74.43	19.47	5.6	29	12.2	154	649.5	1052.81	1.62	113.0451	36.8502	1610	6.78	1.29	0.51	62	874	Primary
274	TPTF	5.31	40.49	13.17	5.8	24	12.4	134	620.0	1004.83	1.62	112.3744	36.9089	1583	6.72	1.52	0.69	39	1406	Primary
275	TPTF	5.50	41.69	13.69	6.4	23	11.8	164	609.3	1004.83	1.65	112.4105	36.9039	1470	7.18	0.72	0.43	56	1517	Planted
276	TPTF	6.32	48.07	15.69	4.7	20	10.1	186	636.1	940.23	1.48	112.0477	36.927	1820	7.28	0.79	0.36	38	1698	Primary
277	TPTF	3.01	21.36	7.80	5.7	29	11.6	151	618.1	963.33	1.56	112.3031	36.9299	1600	7.1	1.16	0.27	57	1316	Planted
278	TPTF	6.54	55.89	15.14	5.3	28	13.9	134	630.4	963.33	1.53	112.3952	36.924	1680	7.4	1.23	0.69	66	781	Primary
279	TPTF	7.66	58.4	19.01	5.1	21	14.1	195	633.1	963.33	1.52	112.3598	36.9351	1720	7.49	0.86	0.49	36	2032	Primary
280	TPTF	5.85	45.44	14.35	4.6	27	13.3	139	641.1	963.33	1.50	112.3064	36.9782	1819	6.8	1.54	0.28	52	1362	Planted
281	TPTF	4.96	44.61	11.15	4.8	22	11.3	178	637.9	963.33	1.51	112.3577	36.9765	1781	6.56	1.31	0.2	61	418	Primary
282	TPTF	4.72	38.02	11.32	4.4	22	9.4	174	643.8	967.25	1.50	112.2824	36.9881	1850	6.81	1.28	0.24	46	851	Primary
283	TPTF	3.82	29.69	9.38	4.6	25	10.3	133	642.0	963.33	1.50	112.3788	36.9823	1813	7.46	0.46	0.54	36	892	Primary
284	TPTF	6.72	53.87	16.19	4.6	20	12.7	131	639.1	963.33	1.51	112.3057	37.003	1820	7.37	1.51	0.62	41	1261	Primary
285	TPTF	5.26	43.55	12.41	5.3	21	13.0	164	621.9	963.33	1.55	112.3564	37.0007	1670	7.36	0.67	0.38	51	782	Planted
286	TPTF	3.08	22.76	4.58	10.5	24	16.3	151	854.9	1103.85	1.29	120.0003	37.0043	240	7.1	0.71	0.33	15	7095	Second
287	TPTF	3.20	24.32	7.96	5.2	28	14.1	202	622.2	981.5	1.58	112.3303	37.0269	1690	7.38	0.79	0.24	46	871	Primary
288	TPTF	3.71	29.41	8.97	4.3	21	10.6	183	646.0	963.33	1.49	112.3747	37.0229	1865	7.41	1.28	0.44	41	742	Primary
289	TPTF	4.62	38.01	10.94	5.5	26	11.1	170	618.1	963.33	1.56	112.4216	37.0252	1630	6.64	0.61	0.35	46	716	Primary
290	TPTF	4.80	40.42	11.20	5.3	27	13.4	158	641.6	978.44	1.53	113.4598	37.1271	1600	7.47	1.04	0.47	62	631	Primary
291	TPTF	5.42	45.21	12.74	4.0	23	13.3	157	671.4	978.44	1.46	113.5242	37.1827	1856	7.24	0.71	0.67	51	767	Planted
292	TPTF	6.39	59.81	12.67	10.0	20	19.0	141	906.1	1110.53	1.23	121.7093	37.2056	275	7.16	0.81	0.33	24	3812	Second
293	TPTF	6.13	45.47	15.45	6.8	28	16.7	135	573.3	100.86	0.18	112.5157	37.2368	1320	7.01	0.98	0.34	40	1960	Primary
294	TPTF	8.21	26.42	8.26	6.2	20	13.4	170	547.6	983.94	1.80	111.2391	37.252	1500	7.39	1.06	0.26	43	1479	Primary
295	TPTF	4.72	38.1	11.31	4.7	27	13.2	139	646.9	966.65	1.49	113.5986	37.2742	1700	7.13	1.6	0.34	41	837	Primary
296	TPTF	9.41	33.49	8.79	6.0	29	14.3	147	547.2	983.94	1.80	111.1815	37.2832	1530	6.53	0.87	0.21	37	2409	Planted
297	TPTF	7.54	27.59	7.03	6.7	24	12.6	169	534.2	983.94	1.84	111.2393	37.3087	1395	7	0.48	0.57	49	2161	Planted
298	TPTF	4.39	37.37	10.18	5.4	25	13.3	142	622.0	966.65	1.55	113.4888	37.3258	1551	7.01	1.36	0.65	53	535	Planted
299	TPTF	4.14	32.04	10.17	5.6	21	11.9	147	614.3	978.51	1.59	113.3764	37.3398	1520	7.24	0.64	0.46	35	984	Primary
300	TPTF	5.32	38.4	13.64	5.8	22	12.0	140	549.1	988.85	1.80	111.3264	37.3516	1550	7.48	1.16	0.26	33	2061	Primary
301	TPTF	3.43	27.95	8.19	5.9	22	15.2	204	604.7	966.65	1.60	113.4884	37.4028	1445	6.71	0.6	0.52	42	575	Planted

302	**TPTF**	4.68	36.22	11.52	5.6	27	13.0	141	607.3	982.14	1.62	113.4626	37.4251	1490	6.78	1.44	0.36	46	1125	Primary
303	**TPTF**	4.37	33.83	10.73	5.5	23	14.3	165	558.4	993.44	1.78	111.7739	37.4747	1575	7.07	1.3	0.4	52	1037	Planted
304	**TPTF**	6.41	61.21	13.87	5.1	22	13.7	187	568.5	1031.97	1.82	111.8171	37.48	1650	7.12	0.98	0.53	62	353	Primary
305	**TPTF**	5.62	46.29	13.29	5.9	29	15.2	164	592.4	965.45	1.63	113.3157	37.4722	1430	6.83	1.27	0.57	55	864	Planted
306	**TPTF**	4.15	34.77	9.71	5.1	22	11.3	207	611.3	965.45	1.58	113.3584	37.4788	1590	6.85	0.74	0.38	65	566	Primary
307	**TPTF**	7.56	67.52	17.06	5.4	25	14.3	177	558.4	993.44	1.78	111.7044	37.4777	1595	7.24	0.44	0.59	53	667	Planted
308	**TPTF**	6.27	52.91	14.63	4.9	21	14.7	172	615.0	965.74	1.57	113.282	37.4706	1641	6.62	1.28	0.6	56	817	Planted
309	**TPTF**	6.34	53.41	14.81	5.2	25	11.4	166	609.7	965.45	1.58	113.3307	37.4765	1582	6.71	0.95	0.25	50	837	Planted
310	**TPTF**	7.61	68.98	17.02	5.1	23	11.4	154	609.9	965.74	1.58	113.2791	37.4716	1600	6.61	1.08	0.21	74	606	Primary
311	**TPTF**	7.77	72.84	17.00	5.3	21	14.2	151	562.3	993.44	1.77	111.7525	37.4884	1620	6.81	1.23	0.39	64	486	Primary
312	**TPTF**	4.32	41.65	9.30	4.6	28	12.2	184	571.9	958.87	1.68	111.5993	37.5027	1750	6.55	0.45	0.59	61	223	Primary
313	**TPTF**	6.33	63.09	13.36	5.0	27	12.3	197	567.3	993.44	1.75	111.7295	37.5079	1680	6.74	0.6	0.64	61	260	Primary
314	**TPTF**	5.00	38.29	12.39	6.0	29	11.8	176	546.5	1031.97	1.89	111.8688	37.5073	1460	6.94	1.31	0.68	55	1292	Planted
315	**TPTF**	10.29	95.13	22.80	5.2	28	11.8	151	559.8	993.44	1.77	111.6662	37.5243	1645	6.72	1.26	0.56	63	836	Primary
316	**TPTF**	4.99	40.59	11.90	4.2	26	9.3	186	575.0	986.12	1.71	111.6619	37.6368	1820	7.31	0.69	0.62	52	838	Planted
317	**TPTF**	7.38	60.22	17.57	4.9	26	11.8	165	558.0	986.12	1.77	111.7087	37.6345	1680	7.41	0.62	0.6	52	1217	Planted
318	**TPTF**	9.36	82.01	21.35	6.1	25	14.7	167	530.3	986.12	1.86	111.7535	37.6329	1424	7.04	1.09	0.61	56	940	Planted
319	**TPTF**	5.03	39.23	12.30	6.7	26	15.8	148	520.9	1012.23	1.94	111.849	37.6358	1300	6.98	1.47	0.26	50	1133	Planted
320	**TPTF**	11.15	108.11	23.92	4.5	28	11.1	137	562.1	938.48	1.67	111.5974	37.6596	1750	6.53	1.12	0.45	61	553	Primary
321	**TPTF**	5.97	54.58	13.28	5.4	26	12.7	197	541.7	986.12	1.82	111.632	37.6575	1575	7.48	1.32	0.59	66	448	Primary
322	**TPTF**	5.16	44.82	11.84	6.7	26	11.7	206	516.1	986.12	1.91	111.7375	37.6517	1300	7.23	1.15	0.61	56	552	Planted
323	**TPTF**	5.46	48.3	12.36	6.0	24	11.2	158	533.9	1012.23	1.90	111.8261	37.655	1450	7.36	1.37	0.45	54	509	Planted
324	**TPTF**	5.49	54.43	11.64	4.3	28	11.0	207	566.8	938.48	1.66	111.5725	37.671	1800	7.44	1.42	0.33	71	235	Primary
325	**TPTF**	10.37	29.03	13.32	7.0	22	12.1	189	516.0	1036.51	2.01	111.9784	37.673	1230	6.92	1.28	0.64	30	1058	Planted
326	**TPTF**	3.68	29.31	8.89	6.4	19	15.6	178	522.6	1012.23	1.94	111.8274	37.6893	1360	7.07	0.71	0.69	33	717	Primary
327	**TPTF**	10.42	107.17	21.59	3.3	28	12.1	196	586.8	978.32	1.67	111.4308	37.7031	1985	7.2	1.55	0.57	55	345	Planted
328	**TPTF**	5.78	48.44	13.54	5.5	23	12.1	195	536.5	986.12	1.84	111.6658	37.7073	1550	7.41	0.75	0.65	58	791	Planted
329	**TPTF**	3.62	29.64	8.60	6.1	20	13.7	204	523.6	986.12	1.88	111.7099	37.7076	1420	6.5	0.58	0.65	51	583	Planted
330	**TPTF**	11.52	112.41	24.63	4.9	25	14.7	164	544.5	938.48	1.72	111.5026	37.7264	1670	7.2	0.88	0.67	56	549	Planted
331	**TPTF**	5.17	42.93	12.17	5.2	29	14.5	165	547.4	1012.23	1.85	111.8616	37.7257	1600	6.91	0.41	0.67	47	751	Planted
332	**TPTF**	2.60	20.88	6.27	7.3	29	13.5	151	553.1	1017.07	1.84	113.5989	37.728	1100	6.87	0.48	0.3	47	485	Planted
333	**TPTF**	4.22	43.13	8.78	4.9	28	13.9	148	543.2	938.48	1.73	111.4898	37.7382	1670	7.24	0.88	0.23	51	146	Planted
334	**TPTF**	6.11	52.9	14.02	5.2	21	11.8	149	538.8	938.48	1.74	111.6172	37.7392	1600	7	1.09	0.27	42	663	Planted
335	**TPTF**	7.35	65.05	16.66	4.9	20	13.4	173	546.4	986.12	1.80	111.6607	37.7328	1650	7.08	1.14	0.37	56	687	Planted
336	**TPTF**	5.63	51.91	12.45	6.1	28	11.6	161	520.5	986.12	1.89	111.7081	37.7369	1410	7.38	0.96	0.62	56	397	Planted
337	**TPTF**	5.84	53.66	12.93	4.6	27	10.4	159	558.4	1012.23	1.81	111.8486	37.7396	1700	6.97	0.53	0.58	59	420	Planted
338	**TPTF**	8.32	75.17	18.64	3.5	22	10.3	152	583.7	972.52	1.67	111.6458	37.7556	1940	7.3	0.53	0.33	57	677	Planted
339	**TPTF**	5.06	42.09	11.91	5.3	28	13.1	149	538.5	965.23	1.79	111.7902	37.7516	1560	6.63	0.9	0.24	59	731	Planted
340	**TPTF**	3.73	33.89	8.33	6.1	27	15.9	149	521.9	965.23	1.85	111.8221	37.7546	1400	6.59	1.56	0.62	66	292	Primary
341	**TPTF**	4.72	41.28	10.79	5.4	27	10.7	190	539.5	965.23	1.79	111.8632	37.7504	1550	7.01	1.13	0.63	66	482	Primary
342	**TPTF**	6.35	54.75	14.61	5.3	22	15.2	199	541.7	965.23	1.78	111.914	37.7539	1555	7.13	1.54	0.62	55	709	Planted
343	**TPTF**	5.39	40.77	13.45	4.3	28	13.3	139	565.1	978.88	1.73	111.9508	37.7865	1750	6.84	0.73	0.47	41	1519	Primary
344	**TPTF**	4.06	35.59	9.25	4.4	29	9.8	186	554.6	972.52	1.75	111.7023	37.8071	1740	7.26	1.13	0.28	56	404	Planted
345	**TPTF**	4.19	32.67	10.25	5.2	21	12.9	199	543.5	978.88	1.80	111.9879	37.8062	1580	6.94	1.13	0.3	56	947	Planted

346	TPTF	4.09	32.33	9.93	5.6	28	13.3	182	541.0	1036.51	1.92	112.3269	37.8262	1475	6.66	1.46	0.28	42	841	Planted
347	TPTF	4.47	38.64	10.28	4.6	27	13.9	171	533.8	941.25	1.76	111.369	37.9055	1711	7.14	1.35	0.58	62	494	Primary
348	TPTF	13.81	114	32.80	4.6	28	14.5	194	536.4	950.37	1.77	111.5062	37.9012	1692	7.4	0.64	0.48	30	3013	Planted
349	TPTF	3.97	29.89	9.94	5.5	25	12.8	187	526.1	1025.04	1.95	111.9853	37.9207	1497	7.32	0.95	0.35	58	1157	Planted
350	TPTF	2.99	21.43	7.68	5.7	22	11.9	189	540.1	1027.11	1.90	112.988	38.0275	1400	7.24	0.62	0.39	27	1208	Planted
351	TPTF	6.23	53.45	14.38	4.6	28	12.1	187	514.1	977.76	1.90	111.2574	38.1095	1680	7.3	0.63	0.32	48	722	Planted
352	TPTF	5.94	50.29	13.84	5.8	19	14.3	153	513.5	1002.54	1.95	112.3918	38.1389	1390	6.94	1.57	0.57	56	760	Planted
353	TPTF	5.26	45.56	12.07	4.5	25	10.5	158	507.3	968.36	1.91	111.3233	38.224	1667	7.17	0.77	0.43	54	569	Planted
354	TPTF	5.61	53.83	12.12	4.1	20	9.9	160	518.0	954.64	1.84	111.3726	38.2533	1750	6.86	0.86	0.43	52	301	Planted
355	TPTF	5.90	48.14	14.07	3.0	29	12.0	135	539.6	954.64	1.77	111.4323	38.3831	1940	7.24	0.96	0.47	51	980	Planted
356	TPTF	3.53	25.81	8.97	6.5	28	14.7	157	476.3	1007.82	2.12	112.4051	38.4225	1200	6.51	1.27	0.6	31	1248	Primary
357	TPTF	7.30	61.25	17.09	4.6	29	10.7	179	490.4	951.39	1.94	111.3789	38.4325	1620	7.37	0.93	0.35	50	991	Planted
358	TPTF	7.55	65.69	17.31	4.4	23	13.3	154	493.7	951.39	1.93	111.3967	38.459	1650	7.02	0.72	0.34	68	800	Primary
359	TPTF	4.84	39.42	11.54	4.0	28	10.8	169	499.3	929.19	1.86	111.7302	38.6251	1690	6.54	1.33	0.49	42	809	Planted
360	TPTF	4.81	35.13	12.24	3.8	26	13.5	158	504.3	967.47	1.92	111.9756	38.6851	1700	7.09	1.46	0.23	28	1713	Second
361	TPTF	6.68	55.08	15.80	3.8	23	11.3	135	503.4	948.67	1.88	111.9405	38.6887	1700	7.27	1.32	0.53	51	1024	Planted
362	TPTF	10.54	33.33	12.58	7.2	25	16.6	155	570.7	1127.84	1.98	116.8091	40.3024	553	6.76	1.33	0.69	30	1475	Planted
363	TPTF	6.39	34.74	7.91	7.8	20	12.9	143	613.7	1089.04	1.77	118.9004	40.4064	350	6.46	0.41	0.7	26	2543	Planted
364	TPTF	10.44	103.96	13.22	5.9	24	14.4	147	802.8	942.29	1.17	123.2099	40.501	600	7.01	0.69	0.39	95	1417	Primary
365	TPTF	7.62	48.83	11.24	4.0	24	13.3	192	637.8	1089.06	1.71	117.9076	40.9022	1100	6.64	1.21	0.54	25	1533	Primary
366	TPTF	11.14	76.57	17.70	4.8	19	10.5	152	635.1	1060.93	1.67	118.6028	41.0075	898	6.32	1.19	0.65	42	1380	Planted
367	TPTF	7.76	89.63	29.07	2.9	28	8.7	163	469.1	1045.87	2.23	119.3035	41.6023	1110	6.47	1.04	0.23	36	3363	Primary
368	TPTF	12.08	96.35	22.48	3.1	20	8.8	189	505.5	957.55	1.89	117.3015	42.004	1140	6.91	1.47	0.3	49	699	Planted
369	TPTF	5.80	83.04	26.98	3.7	20	11.8	163	403.3	1052.11	2.61	118.9791	42.2755	843	7.43	0.45	0.6	35	1793	Primary
370	TPTF	5.81	55.73	11.85	3.7	28	9.1	169	836.9	758.83	0.91	129.5061	42.6003	560	6.6	1.51	0.26	30	2343	Planted
371	TSPF	2.02	33.66	12.42	3.2	31	12.4	145	676.1	1018.18	1.51	115.6007	39.8051	1500	6.78	1.54	0.33	31	2340	Primary
372	TSPF	4.88	59.38	20.41	3.2	26	10.9	163	645.3	1124.58	1.74	115.4399	39.9784	1492	6.12	0.84	0.63	58	1075	Planted
373	TSPF	2.45	50.34	12.96	15.5	31	25.0	205	1147.9	923.34	0.80	107.8517	27.7837	680	7.78	1.3	0.46	27	626	Second
374	TSPF	7.78	122.29	57.32	11.4	29	18.5	184	1282.8	934.3	0.73	105.9059	26.7054	1800	6.43	1.18	0.51	25	1325	Second
375	TSPF	6.90	143.89	69.78	16.0	33	24.4	206	1240.9	984.64	0.79	108.0965	25.7508	748	7.54	1.43	0.67	36	724	Primary
376	TSPF	4.41	76.37	28.72	3.7	29	12.3	210	646.5	1089.06	1.68	117.9032	40.9002	1170	7.29	1.26	0.24	31	2001	Primary
377	TSPF	3.86	80.32	28.38	2.0	34	12.0	131	533.4	993.87	1.86	115.2082	40.9075	1600	6.74	1.53	0.31	50	1035	Planted
378	TSPF	3.09	57.5	20.88	3.1	24	12.8	178	544.4	980.23	1.80	116.3074	41.2085	1300	7.14	0.63	0.68	40	2040	Primary
379	TSPF	4.11	88.22	30.90	2.7	28	8.9	221	734.2	1078.13	1.47	118.4041	40.6045	1400	6.56	0.68	0.42	42	855	Planted
380	TSPF	3.18	61.3	22.09	2.9	25	9.2	206	549.4	979.4	1.78	117.208	41.6094	1260	6.69	1.58	0.62	33	1560	Primary
381	TSPF	2.79	56.48	20.10	1.0	24	8.6	187	568.1	944.18	1.66	117.6026	41.9094	1600	6.89	1.43	0.51	37	915	Planted
382	TSPF	3.55	65.61	23.85	0.9	29	9.4	135	538.9	957.55	1.78	117.3075	42.0062	1600	6.55	0.65	0.66	30	2460	Second
383	TSPF	4.99	117.2	38.79	2.4	26	9.1	215	661.8	758.79	1.15	130.5094	45.7025	350	6.26	2.12	0.65	78	518	Primary
384	TSPF	4.05	97.44	32.17	2.7	28	8.9	149	698.2	795.3	1.14	129.7087	44.7057	450	6.3	1.98	0.58	66	527	Primary
385	TSPF	4.49	106.04	34.94	3.4	29	11.0	207	681.9	739.66	1.08	128.4044	44.3051	400	6.31	1.9	0.23	89	403	Primary
386	TSPF	2.52	45.57	18.46	-2.8	28	2.6	164	457.1	649.05	1.42	126.6061	51.7092	261	6.18	1.95	0.35	36	1595	Primary
387	TSPF	3.73	79.81	28.34	-1.2	31	5.9	169	365.3	649.05	1.78	112.6022	51.7093	507	5.85	2.15	0.46	79	938	Primary
388	TSPF	7.46	123.29	53.12	-3.4	26	4.2	210	478.2	605.55	1.27	124.7077	52.0074	400	6.36	1.92	0.53	44	1026	Planted
389	TSPF	7.21	103.57	40.00	2.1	27	8.3	216	723.2	725.68	1.00	133.5024	46.3021	226	5.25	2.09	0.57	61	578	Primary

390	TSPF	5.81	131.63	39.65	-0.5	32	8.2	166	603.4	781.06	1.29	130.2087	46.3084	880	5.2	2.14	0.61	53	782	Planted
391	TSPF	2.71	65.14	21.28	1.1	30	9.6	190	737.9	763.28	1.03	130.2061	44.5019	800	5.07	2.03	0.67	77	184	Primary
392	TSPF	4.87	94.78	28.98	2.5	32	11.7	138	754.0	764.79	1.01	129.2072	43.8093	650	6.37	1.77	0.39	49	800	Planted
393	TSPF	8.83	101.42	45.43	1.9	32	10.0	144	719.4	713.19	0.99	134.0084	46.8001	170	5.89	2.03	0.49	81	254	Primary
394	TSPF	7.11	94.17	39.96	2.7	34	11.5	173	635.4	806.01	1.27	128.2037	45.0006	450	5.17	2.26	0.63	26	1474	Second
395	TSPF	3.50	135.36	30.59	2.6	24	9.7	148	596.5	818.55	1.37	127.5521	45.3211	455	5.33	2.29	0.41	32	2046	Planted
396	TSPF	4.52	60.54	8.58	-4.3	28	5.5	176	525.1	605.85	1.15	124.5096	52.5091	500	5	1.7	0.63	45	500	Primary
397	TSPF	7.29	123.07	40.47	2.5	31	11.7	235	626.5	844.35	1.35	127.6067	44.902	533	6.64	1.95	0.69	44	864	Planted
398	TSPF	4.84	105.46	36.79	-1.7	27	4.3	181	343.5	708	2.06	128.4054	49.5015	700	5.03	2.05	0.23	63	697	Primary
399	TSPF	7.11	107.16	41.49	0.5	28	6.7	160	532.5	731.23	1.37	129.0052	47.0016	600	6.04	1.93	0.66	68	396	Primary
400	TSPF	4.91	96.71	34.27	1.4	24	6.7	155	653.1	734.78	1.13	131.8009	46.5053	400	6.4	1.86	0.7	66	407	Primary
401	TSPF	6.00	76.86	7.60	3.6	33	10.1	187	796.9	806.67	1.01	128.2774	42.7576	600	5.38	2.12	0.49	35	3018	Primary
402	TSPF	6.12	99.14	40.83	3.9	34	12.9	234	815.4	760.47	0.93	128.1031	42.4084	590	5.25	2.06	0.6	58	1693	Planted
403	TSPF	9.41	134.71	61.90	3.3	24	10.7	178	863.4	846.29	0.98	126.9003	41.8049	845	5.12	1.8	0.34	83	724	Primary
404	TSPF	6.93	87.2	38.56	1.9	26	9.5	137	781.4	801.89	1.03	128.1007	43.505	817	5.62	2.3	0.59	40	6975	Primary
405	TSPF	8.72	127.47	56.25	3.5	33	9.6	207	842.9	739.38	0.88	128.8092	42.4091	650	5.79	2.19	0.38	41	3160	Primary
406	TSPF	4.23	61	19.97	4.7	24	11.4	214	794.6	824.12	1.04	126.4053	41.9085	550	6.79	2.13	0.55	30	1030	Second
407	TSPF	7.85	119.07	36.08	4.3	30	9.6	210	759.0	864.69	1.14	125.7055	42.2032	600	5.32	1.77	0.24	30	1450	Second
408	TSPF	8.75	148.53	37.80	2.9	25	8.7	209	638.7	858.56	1.34	126.9077	44.4034	550	5.96	2.05	0.45	78	666	Primary
409	TSPF	7.01	101.85	42.73	2.9	32	9.7	181	677.6	835.41	1.23	127.2012	44.0095	590	6.53	2.1	0.42	27	2935	Second
410	TSPF	7.43	124.79	51.47	2.7	33	7.7	183	802.8	801.73	1.00	129.7063	43.3029	650	6.11	1.82	0.51	68	1244	Primary
411	TSPF	7.15	122.9	46.31	2.1	26	8.4	216	803.0	776.58	0.97	129.2003	43.3074	800	6.13	2.2	0.57	35	967	Primary
412	TSPF	9.24	113.93	50.43	3.0	25	12.9	203	843.1	767.62	0.91	129.4083	42.7036	700	6.05	2.15	0.42	38	728	Primary
413	TSPF	7.35	122.72	46.66	2.3	26	10.1	186	870.2	758.83	0.87	129.5068	42.6004	847	5.95	1.84	0.61	37	2791	Planted
414	TSPF	5.89	84.82	36.60	3.0	27	10.4	150	702.5	830.23	1.18	126.7094	43.5045	650	6.59	2.05	0.45	25	4066	Second
415	TSPF	7.69	132.63	45.47	3.3	29	12.4	145	761.8	834.36	1.10	127.1058	42.9092	684	5.44	2.07	0.57	40	1062	Primary
416	TSPF	7.91	108.92	52.18	5.9	32	14.0	154	540.7	1013.77	1.87	119.4092	42.0031	500	6.52	1.72	0.31	34	1595	Planted
417	TSPF	6.21	97.55	34.26	-3.3	29	3.9	231	442.0	618.04	1.40	121.5014	50.8038	712	5.06	2	0.21	50	1510	Planted
418	TSPF	2.00	84.4	17.20	-0.9	29	7.6	181	523.8	1052.11	2.01	119.0077	42.3023	1750	6.46	1.04	0.2	28	7320	Second
419	TSPF	6.27	105.21	41.79	1.2	26	9.8	215	512.9	1045.87	2.04	119.3064	41.6082	1434	6.71	0.8	0.35	37	2913	Planted
420	TSPF	7.62	128.07	56.36	0.5	26	7.8	180	310.1	763.69	2.46	122.0056	49.5055	150	6.74	0.72	0.27	69	811	Primary
421	TSPF	3.66	78.26	27.24	-3.3	26	3.5	217	441.4	681.3	1.54	124.0078	50.4053	677	6.78	0.45	0.26	58	1188	Planted
422	TSPF	3.66	87.66	29.60	-5.5	28	0.8	166	554.0	649.05	1.17	126.6024	51.7007	800	6.16	0.4	0.44	79	403	Primary
423	TSPF	6.19	85.35	38.21	7.2	28	16.6	154	241.0	1106.71	4.59	109.4051	40.7017	800	5.99	0.61	0.35	38	4255	Primary
424	TSPF	7.23	147.61	54.80	-0.9	28	7.0	193	348.1	740.77	2.13	122.11	48.506	600	5.72	0.42	0.38	68	1822	Primary
425	TSPF	2.77	50.73	16.57	5.5	27	15.2	182	565.6	830.05	1.47	106.2732	35.6011	2125	6.82	0.8	0.35	29	2000	Second
426	TSPF	9.40	46.64	9.41	3.2	26	10.4	160	376.6	879.78	2.34	101.8019	37.0088	2650	5.95	0.81	0.62	39	2774	Primary
427	TSPF	3.83	49.17	18.76	5.9	34	15.3	170	753.3	982.27	1.30	111.986	35.4386	1838	7.13	1.41	0.27	43	1479	Primary
428	TSPF	2.59	35.89	12.25	6.6	33	14.2	213	731.5	982.27	1.34	111.9955	35.4743	1675	7.15	1.54	0.23	55	642	Planted
429	TSPF	1.86	32.06	11.78	6.7	32	12.5	201	628.9	991.1	1.58	111.0201	36.1841	1590	7.47	0.82	0.58	29	2377	Second
430	TSPF	1.98	38.16	13.75	6.0	26	11.7	179	633.5	972.92	1.54	111.0017	36.2704	1700	7.26	1.48	0.5	35	980	Primary
431	TSPF	3.60	82.46	28.29	7.0	26	13.7	187	615.0	990.6	1.61	111.8975	36.5327	1420	7.37	1.21	0.65	57	460	Planted
432	TSPF	2.39	47.63	17.03	4.1	25	10.8	191	677.9	971.47	1.43	112.0509	36.6531	1980	7.5	1.2	0.47	36	898	Primary
433	TSPF	6.04	77.18	28.95	6.4	33	15.4	133	660.2	1057.65	1.60	113.3041	36.6515	1480	7.31	1.57	0.36	34	1814	Planted

434	**TSPF**	2.48	46.12	16.74	4.4	38	13.1	204	655.8	914.41	1.39	112.0567	36.805	1900	7.32	1.52	0.68	27	1626	Second
435	**TSPF**	3.20	69.52	24.22	2.8	26	8.4	231	697.8	914.41	1.31	112.0319	36.8646	2210	6.67	0.54	0.28	59	654	Planted
436	**TSPF**	2.75	59.18	29.66	5.8	30	15.5	189	652.2	1046.74	1.60	113.3634	36.8723	1550	7.2	1.47	0.57	36	1117	Primary
437	**TSPF**	2.98	60.02	29.50	5.0	38	10.5	223	624.9	940.23	1.50	112.0836	36.9765	1750	7.28	1.34	0.55	65	936	Primary
438	**TSPF**	2.44	46.32	16.73	4.3	31	13.5	234	609.9	991.83	1.63	111.2343	37.0828	1910	6.79	0.41	0.31	48	1414	Planted
439	**TSPF**	2.99	64.17	32.28	4.1	36	13.4	148	673.7	978.44	1.45	113.5005	37.1249	1845	7.45	0.62	0.35	30	1489	Second
440	**TSPF**	2.24	44.13	15.83	3.2	26	9.2	139	691.4	978.44	1.42	113.5581	37.2025	2000	7.13	1.24	0.43	35	939	Primary
441	**TSPF**	1.94	42.19	14.70	5.3	34	10.6	162	567.3	959.69	1.69	111.1456	37.2223	1680	6.93	0.46	0.4	59	357	Planted
442	**TSPF**	2.85	61.32	30.65	5.2	34	10.6	171	560.0	970.69	1.73	111.4118	37.4256	1659	6.76	1.05	0.66	48	987	Planted
443	**TSPF**	3.01	65.9	33.03	4.0	38	12.9	148	590.1	958.87	1.62	111.5765	37.4854	1880	6.57	0.87	0.22	40	1025	Primary
444	**TSPF**	4.58	106.89	53.66	4.3	38	13.9	212	584.1	993.44	1.70	111.6886	37.4814	1810	6.69	1.45	0.28	44	782	Planted
445	**TSPF**	3.16	76.9	39.93	4.5	33	14.2	168	572.8	958.87	1.67	111.5767	37.5214	1772	6.62	0.95	0.34	26	2559	Second
446	**TSPF**	3.77	70.34	33.72	4.6	28	14.2	189	570.9	993.44	1.74	111.6679	37.5579	1750	6.96	1.43	0.6	54	885	Planted
447	**TSPF**	2.13	41.89	15.01	3.7	29	10.1	238	592.6	993.44	1.68	111.6339	37.5707	1925	6.66	1.57	0.41	46	873	Planted
448	**TSPF**	3.07	76.49	39.52	5.5	35	10.8	206	529.9	978.32	1.85	111.391	37.6766	1550	7.33	0.56	0.57	35	1488	Primary
449	**TSPF**	3.59	74.38	26.31	2.1	35	7.1	160	630.8	938.48	1.49	111.529	37.6749	2230	6.6	0.66	0.32	39	984	Primary
450	**TSPF**	1.92	39.02	13.87	4.2	38	13.6	208	576.1	1012.23	1.76	111.8663	37.6839	1805	7.09	1.52	0.31	58	601	Planted
451	**TSPF**	2.23	45.91	16.28	6.0	28	13.1	229	520.7	938.48	1.80	111.5288	37.7052	1447	6.9	1.06	0.52	48	651	Planted
452	**TSPF**	2.11	46.91	16.25	2.7	27	12.2	176	604.1	938.48	1.55	111.451	37.7273	2100	6.51	1.42	0.56	66	335	Primary
453	**TSPF**	2.13	46.54	16.20	3.4	34	12.8	174	579.4	950.37	1.64	111.4527	37.7522	1952	7.25	0.74	0.64	54	377	Planted
454	**TSPF**	4.51	89.98	43.74	3.4	39	11.4	237	585.2	950.37	1.62	111.5717	37.7596	1965	7.5	0.69	0.48	61	943	Primary
455	**TSPF**	3.45	81.29	41.64	4.2	30	10.2	144	557.3	950.37	1.71	111.483	37.7753	1798	6.96	0.58	0.44	38	1779	Primary
456	**TSPF**	3.45	75.35	26.25	2.9	33	8.6	185	596.9	950.37	1.59	111.5954	37.7801	2050	7.16	0.64	0.66	46	629	Planted
457	**TSPF**	2.93	63.37	31.75	4.1	26	10.3	232	551.1	941.25	1.71	111.3935	37.852	1810	6.61	1.41	0.59	32	1113	Planted
458	**TSPF**	3.78	82.96	41.26	3.8	34	9.4	177	550.7	936.7	1.70	111.3682	37.9315	1850	7.14	0.64	0.42	51	814	Planted
459	**TSPF**	2.59	55.2	19.38	3.0	37	12.1	218	579.4	924.89	1.60	111.5778	37.9399	2000	6.95	0.64	0.21	33	576	Primary
460	**TSPF**	3.07	61.1	21.85	3.3	38	13.1	213	572.0	924.89	1.62	111.5935	37.9399	1947	7.03	0.51	0.38	37	1159	Planted
461	**TSPF**	2.02	38.33	13.85	4.0	34	9.8	201	547.1	924.89	1.69	111.56	37.9769	1799	7.4	0.91	0.44	40	1108	Primary
462	**TSPF**	2.43	46.01	22.69	4.6	24	10.1	164	527.0	936.7	1.78	111.4309	37.9845	1680	7.29	0.98	0.56	41	1676	Primary
463	**TSPF**	4.03	78.13	37.65	4.1	28	13.9	191	530.7	936.7	1.77	111.3211	38.0837	1780	6.97	0.82	0.56	36	788	Primary
464	**TSPF**	3.15	68.51	23.89	4.1	27	11.3	160	516.6	954.64	1.85	111.3293	38.2584	1751	7.1	0.76	0.58	31	585	Primary
465	**TSPF**	2.50	53.34	18.71	2.3	36	7.8	144	564.7	954.64	1.69	111.4384	38.3299	2078	7.01	0.68	0.64	47	542	Planted
466	**TSPF**	2.82	59.59	20.97	2.8	24	9.9	178	547.8	954.64	1.74	111.3943	38.3581	1990	6.73	0.47	0.23	48	669	Planted
467	**TSPF**	5.50	103.02	49.71	3.3	33	9.8	197	533.5	954.64	1.79	111.435	38.354	1888	6.69	1.29	0.34	32	1659	Planted
468	**TSPF**	2.70	56.24	28.06	4.7	37	12.5	144	483.1	951.39	1.97	111.373	38.4744	1580	6.88	1.4	0.6	37	1224	Planted
469	**TSPF**	3.11	60.87	21.85	2.7	35	9.7	223	542.1	986.01	1.82	111.6276	38.5225	1970	6.51	0.83	0.23	38	1344	Primary
470	**TSPF**	3.10	62.93	30.82	2.3	28	10.0	180	547.6	929.19	1.70	111.7887	38.6548	2020	7.04	1.12	0.63	37	758	Planted
471	**TSPF**	2.09	41.85	14.93	1.5	32	7.0	208	549.0	950.51	1.73	112.9538	39.3018	2030	7.31	0.5	0.31	49	741	Planted
472	**TSPF**	2.31	40.73	14.92	0.8	29	8.2	139	542.9	980.11	1.81	113.9746	39.9377	2010	6.81	0.6	0.26	35	2449	Primary
473	**TSPF**	5.23	95.81	35.82	4.3	38	10.3	151	875.6	875.09	1.00	107.9039	33.5093	2637	7.52	1.4	0.31	44	4167	Planted
474	**TSPF**	4.22	57.64	19.86	7.7	27	15.7	168	721.9	863.2	1.20	106.1097	33.3016	2060	7.05	1.47	0.26	52	335	Planted
475	**TSPF**	4.94	85.64	32.61	6.9	26	12.6	237	829.8	858.37	1.03	108.3038	33.3044	2137	6.92	0.82	0.35	48	1781	Planted
476	**TSPF**	4.56	52.56	19.72	7.7	26	15.7	229	799.9	858.37	1.07	108.4069	33.401	1950	6.68	0.91	0.53	47	432	Planted
477	**TSPF**	5.43	101.71	34.79	6.4	38	12.2	164	829.0	822.6	0.99	108.5062	33.5052	2210	7	0.47	0.62	49	1320	Planted

478	**TSPF**	4.37	108.02	36.03	9.0	25	14.1	172	746.5	880.82	1.18	108.0093	33.5094	1700	7.2	0.91	0.53	56	320	Planted
479	**TSPF**	4.02	82.15	29.16	9.3	37	18.1	205	751.7	883.55	1.18	107.5083	33.2064	1700	6.77	0.46	0.52	65	1215	Primary
480	**TSPF**	4.48	92.41	29.57	4.1	25	12.6	205	891.5	814.8	0.91	104.1946	32.9837	3200	6.64	2.54	1.01	83	500	Primary
481	**TSPF**	7.25	106.8	53.84	5.9	31	11.0	158	555.0	933.4	1.68	98.8023	31.2069	3300	6.9	2.06	0.73	45	2300	Primary
482	**TSPF**	3.81	84.17	29.05	8.5	36	17.9	162	831.0	906.85	1.09	102.7095	30.6075	2580	6.7	2.52	0.75	52	704	Planted
483	**TSPF**	8.03	176.37	55.50	7.8	32	16.3	228	834.6	719.14	0.86	103.1051	31.403	2600	6.69	2.11	1.13	94	1009	Primary
484	**TSPF**	2.52	50.09	17.50	3.6	28	12.7	204	953.4	901.09	0.95	104.8378	33.5064	3200	6.98	2.03	0.61	49	780	Planted
485	**TSPF**	4.43	88.59	29.33	3.6	26	13.5	223	963.0	901.09	0.94	104.9185	33.5382	3200	6.57	2.37	1.02	98	780	Primary
486	**TSPF**	7.69	115.75	42.35	3.9	34	10.6	190	810.7	765.67	0.94	103.6943	33.5728	3180	7	2.25	0.84	110	1200	Primary
487	**TSPF**	5.92	96.85	28.56	3.9	34	9.2	158	829.3	799.66	0.96	103.7741	33.3379	3200	6.75	2.5	0.79	72	670	Primary
488	**TSPF**	5.10	116.22	39.37	7.3	37	14.0	171	780.1	750.96	0.96	102.3091	30.9013	2850	6.51	2.29	0.85	63	679	Primary
489	**TSPF**	3.94	87.08	26.37	9.9	31	17.4	205	857.6	907.84	1.06	103.1097	30.9069	2185	6.86	2.32	0.77	82	430	Primary
490	**TSPF**	10.61	190.66	87.09	11.2	30	21.1	172	582.3	1004.04	1.72	95.0058	30.103	2300	8.02	1.56	0.7	50	496	Planted
491	**TSPF**	2.79	57.29	24.46	6.3	28	14.0	199	568.0	853.6	1.50	93.3084	29.5045	3355	8.75	1.56	0.68	58	672	Planted
492	**TSPF**	1.85	45.03	15.12	6.7	33	13.4	180	369.6	865.73	2.34	85.2004	28.9005	3500	8.16	1.59	0.8	50	149	Planted
493	**TSPF**	6.70	112.29	49.29	12.1	30	20.3	250	712.9	1008.09	1.41	96.7078	28.7067	2335	7.62	0.78	1.06	81	278	Primary
494	**TSPF**	6.38	95.49	31.22	14.1	25	22.0	170	742.6	1002.27	1.35	97.009	28.4013	2010	7.56	1.17	0.82	50	2082	Planted
495	**TSPF**	7.39	154.48	55.46	9.3	36	19.0	245	1149.0	1039.9	0.91	99.201	27.1053	3012	5.39	0.95	1.26	72	1347	Primary
496	**TSPF**	9.54	230.61	75.47	4.9	26	14.6	183	1188.7	909.39	0.77	107.8074	27.1003	3640	5.42	1.32	1.55	222	2250	Planted
497	**TSPF**	3.22	57.33	20.98	7.2	38	16.5	256	1069.9	946.87	0.89	99.7084	27.702	3400	6.36	1.35	1.34	26	3173	Second
498	**SPPF**	6.24	321.9	33.74	12.8	30	17.8	279	739.1	995.63	1.35	97.4054	28.6083	2200	5.71	2.05	1.42	76	171	Primary
499	**SPPF**	11.69	183.5	14.66	11.4	30	21.4	267	1174.2	1024.55	0.87	99.0807	26.16	2636	4.94	2.12	1.24	110	204	Primary
500	**SPPF**	9.70	163.26	10.70	10.3	32	15.9	298	1128.4	1048.53	0.93	99.3246	26.3026	2880	5.33	2.36	1.26	120	89	Primary
501	**SPPF**	6.78	88.36	12.74	8.8	34	17.5	281	1191.6	1028.99	0.86	99.3282	27.8254	3050	6.01	1.96	1.46	35	898	Primary
502	**SPPF**	8.57	125.74	12.24	14.3	33	20.3	270	1079.4	1059.04	0.98	99.3814	25.7265	2020	5.15	2.46	1.27	50	288	Planted
503	**SPPF**	7.45	104.68	11.69	11.1	36	17.7	264	1127.1	1054.8	0.94	99.3858	26.0279	2715	5.74	2.33	1.53	42	400	Planted
504	**SPPF**	8.26	108.74	15.11	9.5	35	18.4	278	1098.4	1005	0.91	99.4486	27.2322	2940	6.18	2.11	1.35	30	956	Second
505	**SPPF**	6.33	97.29	8.24	9.5	32	18.9	293	1104.9	994.13	0.90	99.45	27.3293	2940	4.96	2.3	1.21	45	134	Primary
506	**SPPF**	10.78	181.01	11.94	11.9	37	18.6	276	1122.2	1065.71	0.95	99.5038	25.7294	2550	4.84	2.13	1.37	50	101	Planted
507	**SPPF**	9.24	132.66	13.81	9.4	32	15.2	284	1081.6	1020.14	0.94	99.5094	27.1751	2980	5.31	1.96	1.27	50	389	Planted
508	**SPPF**	12.99	225.34	13.57	9.0	36	18.2	279	1075.1	1040.1	0.97	99.5781	27.3202	3040	5.23	2.36	1.57	110	91	Primary
509	**SPPF**	6.69	83.44	14.60	14.0	31	22.1	270	1080.5	1090.23	1.01	99.6244	25.431	2104	5.82	2.59	1.33	25	1874	Second
510	**SPPF**	6.81	90.41	12.22	12.9	32	22.5	283	1091.6	1090.23	1.00	99.6297	25.5803	2340	5.83	2.57	1.24	30	714	Second
511	**SPPF**	11.36	179.72	14.12	11.5	35	16.6	281	1088.2	1043.65	0.96	99.6213	25.8765	2635	6.01	2.2	1.44	63	201	Primary
512	**SPPF**	7.65	93.44	19.20	8.9	36	14.8	262	1042.6	1041.31	1.00	99.6844	26.801	3120	5.33	2.07	1.47	25	4330	Second
513	**SPPF**	6.61	89.93	11.15	11.3	33	17.0	268	1031.1	1044.67	1.01	99.6948	27.1058	2540	5.21	2.12	1.32	60	510	Planted
514	**SPPF**	6.46	79.26	17.71	9.4	35	14.8	261	1086.4	946.87	0.87	99.7009	27.7096	2900	5.43	2.05	1.58	22	5675	Planted
515	**SPPF**	6.84	105.01	8.94	14.1	30	22.3	278	1065.6	1066.59	1.00	99.7494	25.3756	2092	5.88	2.19	1.53	50	147	Planted
516	**SPPF**	9.52	124.89	17.57	11.2	31	19.1	263	1155.5	1075.98	0.93	99.7451	25.4361	2750	4.91	2.01	1.46	30	1154	Second
517	**SPPF**	7.13	98.47	11.59	12.5	33	19.4	291	1038.7	1039.03	1.00	99.7424	25.88	2400	5.09	2.14	1.44	45	458	Primary
518	**SPPF**	6.03	79.29	11.07	10.3	32	17.3	275	1017.3	1023.71	1.01	99.7501	26.9522	2780	5.7	2.09	1.27	35	710	Primary
519	**SPPF**	7.68	112.06	11.11	14.7	31	19.8	296	1048.7	1119.92	1.07	99.7985	25.2876	1950	4.83	2.54	1.26	70	275	Primary
520	**SPPF**	6.16	83.52	10.45	15.2	31	23.9	281	983.3	1117.25	1.14	99.8093	25.5821	1800	6.07	2.23	1.28	35	491	Primary
521	**SPPF**	6.98	99.5	10.59	14.7	36	23.3	276	1038.1	1119.92	1.08	99.8592	25.2853	1951	5.69	2.29	1.57	45	317	Primary

522	SPPF	10.94	157.14	18.25	10.2	37	17.0	280	938.2	1082.14	1.15	100.2064	26.8063	2800	6.14	2.48	1.31	68	1487	Primary
523	SPPF	6.60	63.28	18.50	12.9	37	18.4	268	892.2	1187.49	1.33	100.3461	26.3738	2210	5.71	2.45	0.91	45	557	Primary
524	SPPF	6.21	76.01	15.14	8.7	35	18.1	295	912.3	1100.7	1.21	100.4838	27.4519	3060	5.76	2.4	1.02	20	3032	Second
525	SPPF	8.38	116.45	13.47	7.5	32	12.5	278	914.8	1006.81	1.10	100.5461	27.7558	3300	5.61	2.14	1.16	150	507	Primary
526	SPPF	22.71	368.81	26.74	11.1	32	17.4	282	880.1	1081.59	1.23	100.5919	27.1072	2540	5	2.08	0.62	140	289	Primary
527	SPPF	7.01	92.54	12.73	9.0	34	15.3	270	925.7	1006.81	1.09	100.6017	27.8267	2940	5.49	2.22	0.84	150	784	Primary
528	SPPF	12.27	156.63	24.54	10.3	33	15.7	276	875.4	1075.21	1.23	100.6524	27.1084	2730	6.1	2.34	0.8	48	2222	Planted
529	SPPF	6.35	87.27	10.44	7.6	32	15.2	276	892.2	1014.65	1.14	100.6619	27.67	3278	6.11	1.91	0.73	100	431	Primary
530	SPPF	12.72	193.9	18.03	10.2	33	18.0	280	873.1	1164.2	1.33	100.704	26.7075	2779	5.87	2.16	0.61	77	600	Primary
531	SPPF	6.79	85.54	14.23	8.5	36	17.3	290	873.8	1081.87	1.24	100.7248	27.3223	3110	5.35	2.39	1.12	20	1552	Second
532	SPPF	6.83	94.84	10.98	10.5	37	19.1	286	876.5	1095.36	1.25	100.763	26.4596	2735	5.75	2.42	0.85	80	414	Primary
533	SPPF	16.58	278.1	18.40	10.0	34	18.2	273	871.8	1105.04	1.27	100.8949	26.4594	2850	5.6	2.56	0.74	100	157	Primary
534	SPPF	11.11	164.88	19.61	12.5	31	22.2	285	802.5	1263.27	1.57	101.2083	26.6036	2241	5.24	2.43	0.88	71	895	Primary
535	SPPF	7.24	106.35	11.20	9.5	31	17.1	296	823.4	1055.53	1.28	101.2056	27.9097	2850	6.17	2.4	0.77	93	828	Primary
536	SPPF	10.79	156.4	16.05	13.3	38	22.4	285	783.5	1278.09	1.63	101.3467	26.7341	2039	5.1	2.01	0.64	55	512	Planted
537	SPPF	11.40	162.57	20.90	12.9	31	19.1	263	807.8	1303.17	1.61	101.701	26.001	2210	5.01	2.27	0.76	58	914	Planted
538	SPPF	6.42	79.5	8.59	12.4	33	22.0	264	932.3	960.43	1.03	102.5047	28.0085	2068	6.1	2.31	1.19	23	1800	Second
539	SPPF	6.00	74.66	17.53	9.7	32	16.0	300	1541.1	980.69	0.64	104.1392	26.7794	2344	5.2	2.5	1.14	23	7239	Second
540	SPPF	7.33	70.89	20.20	13.2	30	19.8	292	1518.6	1003.94	0.66	104.4122	26.7305	1697	5.07	2	1.2	36	575	Primary
541	SPPF	10.28	165	12.35	17.7	36	23.3	280	1641.7	1006.97	0.61	106.2308	24.7328	970	5.23	2.32	0.93	57	145	Planted
542	SPPF	7.03	111.28	8.67	17.4	35	24.3	265	1654.3	978.15	0.59	106.2942	24.8092	1011	4.94	2.4	0.77	70	113	Primary
543	SPPF	6.48	100.94	8.23	21.1	37	29.3	286	1314.6	1015.81	0.77	106.2933	24.9591	206	5.43	2.09	1.11	48	121	Planted
544	SPMF	8.69	100.99	14.58	22.0	35	30.0	270	1601.8	1070.5	0.67	110.6565	23.0053	175	6.18	1.33	0.44	27	481	Second
545	SPMF	8.44	105.69	15.76	21.4	36	29.9	261	1709.7	1052.5	0.62	113.7013	23.0021	200	6.07	0.85	0.7	18	1089	Planted
546	SPMF	17.54	158.07	84.92	21.3	38	29.0	290	1710.3	1046.2	0.61	112.354	23.0884	270	5.04	1.38	0.27	30	2500	Planted
547	SPMF	8.13	101.91	15.20	19.4	39	27.3	284	1919.5	987.5	0.51	115.1016	23.6018	500	5.89	1.09	0.4	22	1065	Planted
548	SPMF	9.00	164.5	29.10	21.7	36	28.8	283	1414.0	1062.1	0.75	105.0889	23.7559	300	5.38	1.14	0.57	38	435	Planted
549	SPMF	7.51	89.52	13.11	20.0	34	26.9	278	1659.3	1009.8	0.61	111.7709	24.2362	380	5.93	1.42	0.27	28	542	Second
550	SPMF	9.63	112.24	16.22	20.5	35	29.1	276	1562.9	1025.4	0.66	111.7738	24.4544	255	4.85	1.13	0.49	32	546	Planted
551	SPMF	7.77	92.34	13.50	21.1	37	30.0	276	1415.8	1045.2	0.74	108.289	24.5865	215	5.98	0.45	0.45	28	540	Second
552	SPMF	16.61	171.83	45.75	18.2	32	26.0	285	1860.9	953.7	0.51	114.5039	24.7037	600	5.54	1.48	0.59	29	1425	Planted
553	SPMF	9.24	110.21	16.14	19.5	34	28.6	281	1552.2	995.3	0.64	108.8035	24.8055	490	5.23	1.38	0.37	26	671	Planted
554	SPMF	16.71	172.76	46.03	18.2	40	25.4	298	1847.2	952.3	0.52	114.4043	24.8038	600	5.64	1.34	0.69	29	1425	Planted
555	SPMF	10.21	275.02	86.39	19.3	33	29.0	264	1545.7	991.8	0.64	111.3009	25.3096	380	5.74	0.88	0.21	54	563	Planted
556	SPMF	6.64	93.09	11.67	14.9	36	21.0	286	1439.5	855.4	0.59	105.1376	25.5845	1390	5.18	1.11	0.68	20	1161	Planted
557	SPMF	6.82	174.24	45.76	16.1	32	21.7	283	2006.0	889.2	0.44	116.9059	25.7072	820	5.77	0.75	0.49	53	1185	Planted
558	SPMF	7.53	92.67	13.75	18.5	34	28.3	273	1537.9	966.7	0.63	110.6026	25.8075	500	5.02	0.47	0.51	28	775	Second
559	SPMF	8.26	205.32	29.50	18.5	34	24.6	293	1507.8	967.1	0.64	110.1026	25.9088	500	4.87	1.4	0.31	101	534	Primary
560	SPMF	7.73	164.07	16.83	15.2	36	21.5	270	1218.9	889.8	0.73	106.656	26.2374	1050	4.96	0.76	0.66	46	795	Primary
561	SPMF	6.36	141.61	13.51	16.5	37	24.3	300	1233.5	959.1	0.78	108.5952	26.5293	520	5.23	0.52	0.5	36	493	Planted
562	SPMF	6.06	87.96	11.58	15.9	37	22.0	296	1369.5	956.8	0.70	109.3587	26.6339	500	5.06	1.56	0.5	21	1389	Planted
563	SPMF	8.89	82.93	23.44	18.4	34	24.6	271	1358.8	966.1	0.71	109.5946	26.8336	410	5.47	0.94	0.48	30	392	Planted
564	SPMF	9.03	73.97	17.04	18.9	39	26.2	273	1314.8	982.7	0.75	109.7561	26.8359	300	4.93	1.16	0.6	20	1750	Planted
565	SPMF	13.18	149.12	35.71	18.9	34	24.5	269	1302.7	984.7	0.76	109.9024	26.9	275	5.82	0.81	0.34	25	2900	Second

566	**SPMF**	6.43	83.53	9.66	15.8	40	23.7	299	1313.4	950.2	0.72	109.1348	27.039	500	5.23	1.02	0.35	30	708	Planted
567	**SPMF**	10.07	100.95	27.45	16.8	38	22.9	290	1374.4	918.9	0.67	109.5809	27.6214	615	5.69	1.25	0.3	30	689	Planted
568	**SPMF**	6.42	76.04	8.13	13.9	35	21.6	280	1171.4	863.7	0.74	107.614	27.709	1012	5.73	1.35	0.66	28	447	Second
569	**SPMF**	6.92	125.62	22.63	15.8	40	22.2	271	1142.3	925.6	0.81	108.121	28.258	540	5.4	0.43	0.6	30	2120	Planted
570	**SPMF**	10.44	117.49	28.17	16.7	37	23.1	278	1327.5	917.3	0.69	111.1064	28.3068	500	5.75	0.91	0.24	21	2415	Planted
571	**SPMF**	7.66	90.59	9.68	12.2	40	18.1	293	1127.3	802.0	0.71	106.9767	28.4753	1330	5.33	0.61	0.23	28	531	Second
572	**SPMF**	8.84	96.85	24.44	15.3	37	21.1	297	1213.5	876.4	0.72	109.303	29.4058	680	5.65	1.08	0.32	22	1290	Planted
573	**SPMF**	6.93	77.28	16.88	12.7	39	20.8	261	1274.2	794.6	0.62	109.4018	30.209	1100	5.37	1.16	0.61	22	3345	Planted
574	**SPMF**	7.74	127.75	24.27	14.3	32	22.7	266	1141.7	854.0	0.75	114.8019	31.6017	400	5.22	1.31	0.7	30	3200	Planted
575	**SPMF**	7.30	81.8	19.86	13.4	39	21.2	293	1211.8	827.1	0.68	118.2052	32.2009	400	5.99	1.6	0.32	20	1515	Planted
576	**SPMF**	12.35	118.68	24.08	24.0	34	34.0	295	1541.1	1130.3	0.73	108.4092	21.7589	10	6.02	1.27	0.66	39	2213	Planted
577	**SPMF**	10.67	134.33	27.95	21.1	40	28.5	275	1727.0	1040.5	0.60	113.712	23.2221	250	6.17	0.61	0.45	30	1700	Planted
578	**SPMF**	9.48	120.72	17.97	21.4	33	31.3	266	1677.9	1051.1	0.63	113.2015	23.2007	200	5.56	0.98	0.58	23	1782	Planted
579	**SPMF**	13.13	163.02	24.17	19.8	32	29.7	268	1704.6	1002.4	0.59	111.504	24.1043	450	5.59	0.96	0.27	24	1697	Second
580	**SPMF**	15.14	181.06	28.74	18.6	34	24.4	299	1532.7	968.1	0.63	110.0045	25.71	520	6.13	0.46	0.35	30	1109	Planted
581	**SPMF**	9.02	103.11	24.69	18.2	36	23.6	273	1654.6	956.2	0.58	113.3006	25.7064	500	4.82	1.24	0.22	19	2020	Planted
582	**SPMF**	10.78	113.58	30.09	17.3	33	26.2	268	1821.4	928.5	0.51	117.2096	25.8031	550	5.19	0.91	0.52	25	1251	Second
583	**SPMF**	7.63	93.92	15.08	16.9	34	23.7	272	1674.2	915.5	0.55	110.6008	26.0097	800	5.99	0.53	0.28	30	1135	Planted
584	**SPMF**	10.28	124	18.34	18.3	34	26.6	279	1526.2	959.1	0.63	110.4004	26.0044	530	5.47	0.83	0.64	30	937	Planted
585	**SPMF**	11.84	270.36	84.74	18.2	35	27.4	297	1443.7	958.4	0.66	110.1063	26.5004	483	5.48	1.37	0.5	54	795	Planted
586	**SPMF**	13.20	141.36	36.60	18.8	36	25.1	271	1343.4	978.0	0.73	109.6079	26.7003	350	6.16	0.79	0.21	28	1418	Second
587	**SPMF**	12.79	293.72	90.24	18.2	38	24.9	284	1387.4	960.5	0.69	109.9013	26.8011	440	5.19	0.96	0.37	52	797	Planted
588	**SPMF**	8.15	149.52	42.23	15.8	34	21.4	282	1508.1	889.6	0.59	114.7097	28.3021	571	5.57	1	0.59	52	848	Planted
589	**SPMF**	7.51	96.97	19.53	16.9	35	25.0	278	1295.0	927.3	0.72	115.8007	29.009	217	5.6	1.05	0.68	34	915	Planted
590	**SPMF**	7.27	70.68	16.21	12.6	36	22.2	281	1205.6	803.3	0.67	117.9083	32.7074	500	5.24	0.89	0.42	19	2235	Planted
591	**SPMF**	4.70	61.86	9.06	21.8	37	29.0	261	1773.9	1057.2	0.60	107.6526	21.9287	475	5.3	1.31	0.62	16	1920	Second
592	**SPMF**	4.10	53.91	7.91	22.4	34	27.7	289	1675.2	1078.4	0.64	107.0732	21.9869	350	5.24	0.57	0.49	15	1606	Second
593	**SPMF**	2.80	139.9	17.10	21.7	36	27.5	275	1414.0	1062.1	0.75	105.0849	23.7598	300	5.21	1.59	0.33	23	1725	Planted
594	**SPMF**	4.14	218.48	37.51	21.1	35	27.1	277	1445.1	1045.6	0.72	108.6872	24.4237	220	5.7	1.27	0.51	31	838	Planted
595	**SPMF**	3.76	56.55	7.98	13.6	34	23.4	281	1283.9	835.1	0.65	106.0958	26.1756	1420	4.89	1.35	0.58	18	1260	Planted
596	**SPMF**	6.01	76.92	8.78	13.8	35	20.9	295	1310.7	878.6	0.67	108.2453	26.2777	1070	5.3	0.85	0.46	21	612	Planted
597	**SPMF**	6.30	75.88	8.23	16.0	39	24.4	279	1204.7	937.0	0.78	108.0008	26.3356	700	4.93	0.89	0.49	25	476	Planted
598	**SPMF**	3.22	48.18	6.70	14.8	35	21.9	276	1218.2	878.7	0.72	106.7392	26.3359	1106	5.91	0.8	0.48	19	999	Planted
599	**SPMF**	5.77	77.16	9.18	14.6	34	23.3	263	1209.4	892.7	0.74	107.8339	26.8863	920	6.18	1.4	0.45	23	747	Planted
600	**SPMF**	4.75	71.05	9.89	16.0	34	23.0	270	1136.4	902.5	0.79	106.6033	28.0093	760	4.85	0.86	0.35	26	1473	Planted
601	**SPMF**	4.84	70.74	9.43	13.9	38	22.0	299	1193.3	872.9	0.73	108.112	28.0319	900	5.26	1.44	0.43	27	1186	Planted
602	**SPMF**	3.55	79.37	16.99	15.3	36	22.2	282	1642.4	875.1	0.53	119.9042	28.406	500	5.41	0.71	0.5	22	1695	Planted
603	**SPMF**	5.53	49.74	11.32	15.8	32	24.3	290	1450.9	894.9	0.62	119.6053	29.1071	300	5.69	0.41	0.6	20	1725	Planted
604	**SPMF**	3.43	27.5	5.73	17.2	36	22.6	262	1136.4	918.9	0.81	106.6331	29.7279	450	5.26	0.68	0.37	20	3600	Planted
605	**SPMF**	3.22	87.73	18.73	14.4	36	22.7	269	1432.8	850.5	0.59	118.1538	30.1348	500	4.81	0.9	0.44	21	1425	Planted
606	**SPMF**	5.11	57.67	13.09	16.5	34	26.5	263	1022.6	922.4	0.90	112.8063	30.8049	120	6.16	1.09	0.29	19	1845	Planted
607	**SPMF**	5.34	60.03	13.42	13.5	35	23.2	274	1067.9	827.2	0.77	111.2009	31.8098	650	6.14	0.83	0.52	18	2169	Planted
608	**SPMF**	7.17	117.87	21.59	15.9	40	25.1	272	1554.2	893.4	0.57	120.004	28.5054	369	5.53	1.37	0.67	18	2816	Planted
609	**SPMF**	9.29	86.59	19.65	16.9	35	25.3	275	1503.3	926.8	0.62	120.6016	28.0026	200	5.66	1.42	0.5	18	1733	Planted

610	SMPF	11.28	83.07	19.37	8.4	38	16.6	304	1848.0	654.1	0.35	116.1047	31.0096	1700	6.19	1.41	0.55	25	2200	Planted
611	SMPF	13.30	165.02	40.50	11.6	37	17.6	261	1573.7	759.6	0.48	116.3075	30.8062	1050	5.49	0.62	0.52	28	2360	Second
612	SMPF	8.40	168.69	35.52	14.3	38	23.0	279	1802.6	930.9	0.52	119.3032	26.0014	845	4.81	0.84	0.38	102	710	Primary
613	SMPF	11.28	190.63	46.58	13.1	37	18.7	263	2205.0	795.5	0.36	118.9057	27.0088	1200	5.53	1.38	0.22	160	2100	Primary
614	SMPF	8.94	96.25	27.22	13.3	34	19.1	294	2099.8	800.0	0.38	117.106	27.2058	1200	5.54	1.47	0.3	20	2500	Planted
615	SMPF	6.83	46.82	10.61	8.9	35	18.4	276	564.1	645.9	1.15	105.8839	34.5557	1625	4.97	1.02	0.68	26	3500	Planted
616	SMPF	5.58	46.41	6.85	11.1	37	16.1	299	1272.4	750.6	0.59	105.6252	27.1747	1850	5.26	0.68	0.5	19	4767	Planted
617	SMPF	3.54	29.81	4.36	11.4	34	18.4	309	1426.8	743.0	0.52	104.6506	27.0594	1940	5.35	1.18	0.59	17	3639	Planted
618	SMPF	7.18	50.98	8.03	14.0	39	24.0	289	1396.5	818.9	0.59	104.8974	27.1349	1443	4.84	1.43	0.27	16	821	Second
619	SMPF	6.99	59.67	10.50	12.7	35	18.3	280	1208.3	818.4	0.68	106.8247	27.0576	1400	5.13	0.8	0.4	23	674	Planted
620	SMPF	8.78	66.68	10.59	12.5	37	21.0	267	1333.8	786.8	0.59	105.3727	26.7391	1680	5.4	1.29	0.52	23	1520	Planted
621	SMPF	9.56	70.39	10.94	13.1	37	18.6	282	1443.0	804.5	0.56	105.1305	25.69	1700	4.98	1.55	0.38	23	1724	Planted
622	SMPF	3.70	62.27	18.22	10.8	36	19.4	273	1635.5	712.3	0.44	103.8421	26.7068	2200	5.26	1.24	0.67	31	1174	Planted
623	SMPF	5.96	41.93	6.42	9.4	37	15.8	306	1483.5	681.4	0.46	104.337	26.8805	2360	4.86	1.21	0.62	16	899	Second
624	SMPF	5.86	45.76	7.22	9.8	36	16.6	316	1513.6	689.0	0.46	104.1669	27.0237	2300	5.51	0.65	0.66	30	1370	Planted
625	SMPF	8.03	138.37	37.64	10.4	36	15.7	301	1530.2	704.8	0.46	104.2095	26.8046	2225	5.38	1.51	0.61	52	968	Planted
626	SMPF	4.47	37.33	6.05	12.7	36	19.4	301	1596.7	786.7	0.49	104.6551	24.904	1920	5.39	1.16	0.27	20	1202	Second
627	SMPF	10.58	112.89	30.79	12.4	37	19.4	294	1337.7	789.2	0.59	115.5077	31.5068	802	5.78	1	0.48	48	2556	Planted
628	SMPF	9.19	95.73	23.95	12.8	36	21.7	314	1274.6	803.8	0.63	114.8035	31.6071	718	5.32	1.15	0.4	34	1442	Planted
629	SMPF	13.19	78.29	15.78	9.5	39	18.7	318	1412.1	690.5	0.49	110.3094	31.0043	1638	5.43	1.34	0.57	20	3066	Second
630	SMPF	4.88	55.58	20.70	10.3	36	19.0	302	1015.2	916.4	0.90	111.7045	30.8087	1190	5.91	0.69	0.61	20	950	Second
631	SMPF	5.43	57.99	14.12	8.2	36	16.3	303	1392.6	650.3	0.47	110.6086	31.7042	1800	5.51	0.79	0.28	30	1085	Second
632	SMPF	6.48	53.12	7.69	8.4	37	15.8	295	1630.0	658.0	0.40	113.602	31.4085	1700	5.87	1.44	0.27	16	6195	Second
633	SMPF	17.65	255.28	56.83	12.6	37	18.1	280	1242.3	788.7	0.63	108.9052	30.3064	1140	5.48	0.99	0.32	80	840	Primary
634	SMPF	6.47	62.66	17.38	10.1	36	17.5	293	1661.6	711.2	0.43	115.7071	31.0021	1350	5.2	0.97	0.66	18	2536	Second
635	SMPF	7.60	52.06	10.38	13.7	37	21.5	284	1561.3	820.9	0.53	113.8009	29.2088	925	4.99	1.18	0.22	16	1575	Second
636	SMPF	9.05	81.02	13.57	17.0	39	24.1	299	1147.6	936.3	0.82	114.3057	29.7071	120	5.24	0.98	0.61	16	2550	Second
637	SMPF	15.44	95.02	14.46	10.2	36	17.0	311	1480.1	713.3	0.48	111.3093	30.701	1500	5.56	0.52	0.64	21	845	Second
638	SMPF	9.16	82.75	22.32	11.5	37	20.7	282	1845.7	746.3	0.40	113.8033	28.9028	1433	5.01	1.55	0.43	23	3667	Second
639	SMPF	6.13	44.7	6.43	7.7	37	15.6	286	1021.4	657.9	0.64	117.2022	36.4077	1000	5.65	0.44	0.48	18	2545	Second
640	SMPF	5.06	62.87	13.35	11.5	37	16.7	262	692.0	712.6	1.03	105.6023	32.6725	1435	5.84	0.51	0.67	30	4200	Second
641	SMPF	4.73	79.21	28.62	7.5	36	15.5	268	724.3	596.9	0.82	106.6	33.5079	2040	5.52	0.72	0.27	41	920	Primary
642	SMPF	6.60	68.73	17.55	8.6	37	15.9	267	784.8	631.6	0.80	108.3579	33.3558	1787	5.76	1	0.3	29	2825	Second
643	SMPF	7.96	104.46	25.92	9.3	36	16.3	296	751.7	652.1	0.87	107.5041	33.2013	1700	4.92	0.51	0.23	56	640	Planted
644	SMPF	7.00	124.14	39.60	6.7	38	16.7	263	727.3	574.0	0.79	107.208	33.9093	2120	4.97	0.99	0.31	35	2104	Planted
645	SMPF	9.60	80.46	14.81	7.0	36	16.1	265	806.9	505.3	0.63	101.2086	27.9064	3445	4.89	1.43	0.64	64	993	Primary
646	SMPF	11.73	102.87	17.96	7.1	37	16.7	293	712.7	521.1	0.73	101.0007	30.0077	3100	4.95	1.49	0.51	48	1291	Planted
647	SMPF	7.01	122.76	16.90	10.6	35	16.0	313	582.3	655.0	1.12	95.0078	30.107	2414	4.99	1.46	0.21	40	288	Planted
648	SMPF	7.18	133.17	20.45	10.7	35	20.6	310	573.5	655.8	1.14	94.9024	30.2086	2400	6.17	0.98	0.44	40	307	Planted
649	SMPF	7.92	220.78	82.82	8.5	35	17.8	311	619.7	586.4	0.95	95.7051	29.8018	2864	6.11	1.39	0.68	68	266	Primary
650	SMPF	9.24	213.07	28.67	8.5	37	17.4	284	619.7	586.1	0.95	95.7019	29.8066	2866	6.05	1.36	0.66	70	369	Primary
651	SMPF	7.17	102.7	15.26	7.3	37	13.7	318	608.5	550.4	0.90	94.1075	29.209	3210	5.9	1.18	0.46	80	338	Primary
652	SMPF	10.78	143.38	22.22	6.9	38	16.1	307	579.4	539.6	0.93	94.1088	29.7075	3219	6.03	0.96	0.34	71	853	Primary
653	SMPF	9.88	147.41	21.34	11.1	39	18.0	286	369.7	685.1	1.85	85.2003	28.9029	2651	5.25	1.59	0.23	57	589	Planted

654	**SMPF**	6.68	63.41	14.04	10.3	38	15.3	266	620.7	637.0	1.03	91.9031	27.9016	2880	6.15	1.22	0.27	35	1069	Planted
655	**SMPF**	6.62	110.63	16.65	5.7	36	14.2	310	591.3	503.8	0.85	93.1092	29.006	3558	5.55	1.28	0.31	108	316	Primary
656	**SMPF**	5.60	79.51	11.86	9.7	36	15.0	305	603.2	622.6	1.03	92.3046	28.409	2900	5.08	1.53	0.6	90	267	Primary
657	**SMPF**	5.22	148.79	61.46	12.1	35	19.5	276	712.9	683.7	0.96	96.7001	28.7075	2350	5.55	1.23	0.51	55	183	Planted
658	**SMPF**	12.12	100.36	18.25	8.7	38	14.6	296	712.5	581.1	0.82	96.7009	28.7065	3000	5.87	1.14	0.51	80	1178	Primary
659	**SMPF**	11.98	186.05	26.76	10.8	36	17.8	282	563.4	657.0	1.17	88.9019	27.4071	2900	5.47	1.56	0.49	60	509	Planted
660	**SMPF**	8.51	59.47	11.41	7.2	34	14.9	300	1285.8	521.3	0.41	99.207	28.2516	3360	4.91	2.02	0.58	50	1055	Planted
661	**SMPF**	8.89	37.03	7.95	7.4	36	15.3	287	987.6	532.6	0.54	100.1159	26.6728	3450	5.24	2.28	0.41	38	2581	Planted
662	**SMPF**	11.31	91.03	16.71	8.3	37	17.3	310	908.4	564.6	0.62	100.4874	27.3815	3152	6.03	2.19	0.29	60	1145	Planted
663	**SMPF**	12.12	112.92	19.73	7.8	37	14.3	307	1101.7	542.9	0.49	99.5113	27.5336	3300	5.21	2.07	0.35	45	1002	Planted
664	**SMPF**	5.47	20.62	4.46	8.0	36	16.7	301	845.7	554.5	0.66	100.9645	27.2359	3220	5.71	2.41	0.66	20	1931	Planted
665	**SCLF**	6.63	103.84	19.37	22.4	33	28.1	314	2367.1	1064.4	0.45	108.8088	18.7051	780	6.04	0.79	0.67	16	2003	Second
666	**SCLF**	6.29	101.63	20.00	21.4	31	27.4	290	1922.7	1045.6	0.54	110.3077	21.6866	500	6.1	0.95	0.33	20	2750	Planted
667	**SCLF**	5.03	38.36	10.09	20.5	34	30.4	307	1790.1	1021.5	0.57	111.1085	23.1086	460	5.45	1.48	0.24	16	3075	Second
668	**SCLF**	28.17	190.6	63.97	13.6	34	19.8	308	2945.9	797.1	0.27	120.9023	23.7073	1526	5.48	1.44	0.68	21	2177	Planted
669	**SCLF**	10.61	163.81	34.05	21.2	31	31.0	298	1548.3	1047.9	0.68	109.7021	23.8079	250	4.84	0.54	0.6	19	1380	Planted
670	**SCLF**	4.86	43.98	10.77	19.9	33	29.5	275	1725.7	1003.1	0.58	110.8835	23.881	490	5.38	0.55	0.22	25	1379	Planted
671	**SCLF**	18.27	178.54	56.23	14.5	34	21.0	295	2716.7	827.6	0.30	121.1287	24.0367	1287	5.58	0.52	0.5	31	1300	Planted
672	**SCLF**	10.27	52.71	14.74	20.7	32	26.3	311	1454.9	1030.5	0.71	106.2387	24.2736	405	5.57	1.03	0.27	18	3475	Second
673	**SCLF**	5.89	48.17	15.25	18.9	33	24.9	314	1855.6	976.7	0.53	117.6059	24.4014	400	5.48	0.62	0.31	21	2400	Planted
674	**SCLF**	26.70	132.27	45.40	12.1	34	17.9	269	2989.1	752.1	0.25	121.109	24.5206	1707	5.24	0.74	0.63	18	1640	Second
675	**SCLF**	5.90	52.56	12.96	21.0	34	28.1	299	1426.1	1044.7	0.73	108.7527	24.5899	205	5.86	0.78	0.56	16	1796	Second
676	**SCLF**	12.47	51.95	32.27	18.8	32	26.2	268	1815.9	972.9	0.54	117.2057	24.7098	400	5	1.22	0.62	21	1630	Planted
677	**SCLF**	24.10	133.9	45.20	17.8	35	23.2	263	1984.4	941.0	0.47	121.5772	24.836	460	5.82	0.89	0.21	18	1700	Second
678	**SCLF**	5.79	75.4	15.14	18.3	34	28.1	308	1685.0	956.9	0.57	111.0486	25.1749	624	5.43	0.86	0.4	24	2184	Second
679	**SCLF**	4.66	60.73	12.27	17.4	31	24.3	285	1787.2	926.1	0.52	111.1213	25.2304	810	5.85	0.48	0.64	21	1896	Planted
680	**SCLF**	5.36	50.44	12.15	19.5	33	27.2	302	1482.0	995.8	0.67	109.0904	25.3077	419	5.7	1.2	0.65	22	1280	Planted
681	**SCLF**	9.64	44.59	14.24	14.7	33	22.0	308	1437.9	849.7	0.59	105.2179	25.4076	1445	5.54	1.07	0.25	16	2022	Second
682	**SCLF**	8.40	214.6	29.30	18.6	34	26.1	294	1148.8	994.5	0.87	107.1718	25.4807	461	5.35	0.79	0.54	18	1950	Second
683	**SCLF**	11.39	74.1	20.07	17.9	35	25.0	271	1216.3	1002.7	0.82	108.6195	25.5091	365	5.66	0.85	0.44	19	1850	Planted
684	**SCLF**	4.50	42.35	10.20	16.6	35	23.1	297	1784.3	900.5	0.50	110.1068	25.5311	966	5.97	0.8	0.41	17	1068	Planted
685	**SCLF**	7.93	77.69	18.40	17.5	33	23.7	310	1634.5	931.8	0.57	109.8021	25.7505	750	5.71	0.78	0.42	16	1582	Second
686	**SCLF**	7.55	88.31	21.03	19.2	30	29.0	270	1452.5	988.3	0.68	110.0053	25.8076	380	6.04	0.97	0.55	28	1400	Second
687	**SCLF**	9.24	39.98	13.18	16.5	32	24.3	293	1370.4	977.2	0.71	109.402	26.1223	430	5.99	1.12	0.59	30	2038	Planted
688	**SCLF**	14.31	184.78	34.89	18.6	34	24.6	262	1430.8	972.1	0.68	109.5075	26.2082	450	5.63	0.42	0.46	18	2399	Second
689	**SCLF**	13.14	161.73	35.42	18.9	33	26.3	272	1472.3	983.6	0.67	114.5036	26.3095	220	5.3	0.91	0.69	32	1850	Planted
690	**SCLF**	10.28	62.3	17.47	13.5	34	21.6	275	1259.3	831.8	0.66	106.1746	26.4891	1402	6.11	1.36	0.23	26	1760	Planted
691	**SCLF**	11.49	137.91	31.14	18.4	32	27.2	266	1501.3	967.6	0.64	114.4071	26.5003	300	5.19	0.92	0.48	25	2073	Planted
692	**SCLF**	7.32	52.17	13.52	14.3	31	23.1	284	1371.0	903.8	0.66	108.9181	26.5329	860	5.34	0.66	0.5	18	1113	Second
693	**SCLF**	13.98	186.35	44.08	17.1	30	22.7	278	1710.5	925.1	0.54	118.1091	26.6015	450	4.85	0.67	0.62	20	2160	Planted
694	**SCLF**	10.28	542.26	103.50	17.1	34	22.5	262	1710.5	925.1	0.54	118.1001	26.6017	450	5.98	0.9	0.42	39	1716	Planted
695	**SCLF**	13.28	198.01	44.90	17.4	35	24.7	308	1670.4	933.9	0.56	117.9024	26.6051	400	5.47	0.58	0.56	27	1558	Planted
696	**SCLF**	9.73	48.47	14.94	13.8	35	20.9	282	1286.3	831.3	0.65	105.7739	26.6218	1400	6.14	0.64	0.66	16	1928	Second
697	**SCLF**	10.80	153.03	35.37	17.2	34	24.3	318	1662.6	928.8	0.56	118.2021	26.806	400	4.83	0.66	0.52	24	1435	Second

698	**SCLF**	10.84	58.44	17.35	14.4	32	19.8	261	1359.7	908.5	0.67	108.9965	26.8509	790	5.74	1.04	0.47	16	2025	Second
699	**SCLF**	15.55	116.09	29.40	16.3	31	23.0	277	1347.9	967.8	0.72	109.4355	27.0361	380	5.4	0.98	0.34	18	2285	Second
700	**SCLF**	9.99	43.86	14.35	16.2	34	25.5	279	1334.0	963.7	0.72	109.3553	27.1092	400	6.16	1.37	0.49	16	2178	Planted
701	**SCLF**	4.63	60.35	12.24	16.7	33	24.1	289	1492.0	911.9	0.61	110.2362	27.127	700	5.78	1.2	0.58	32	1992	Planted
702	**SCLF**	14.33	74.61	22.52	14.1	34	20.0	264	1185.5	872.4	0.74	107.5665	27.2759	1010	4.85	0.99	0.68	18	2748	Second
703	**SCLF**	4.80	251.53	51.56	17.4	34	22.5	267	1498.5	935.9	0.62	114.6033	27.3007	400	5.27	0.69	0.6	55	1027	Planted
704	**SCLF**	17.40	67.9	23.48	14.7	34	22.9	297	1223.1	903.2	0.74	108.3709	27.5321	760	5.54	0.92	0.55	19	4154	Planted
705	**SCLF**	7.07	33	10.49	14.6	31	24.4	316	1241.3	903.8	0.73	108.6027	27.8247	715	5	0.91	0.62	20	1473	Planted
706	**SCLF**	4.65	61.24	13.30	18.2	31	25.2	299	1215.8	965.7	0.79	111.0031	28.1006	220	4.9	0.57	0.6	23	4978	Planted
707	**SCLF**	6.02	30.81	9.38	15.1	33	24.4	286	1261.3	921.0	0.73	108.9231	28.1359	560	5.07	0.7	0.57	20	1170	Planted
708	**SCLF**	6.78	38.08	11.08	12.2	34	20.7	269	1148.6	794.9	0.69	106.5695	28.1351	1427	5.06	0.68	0.68	32	1227	Planted
709	**SCLF**	5.19	26.16	8.02	14.6	32	23.7	303	1254.9	904.1	0.72	108.8028	28.2251	660	6.02	0.43	0.61	20	1018	Planted
710	**SCLF**	8.32	41.78	12.83	14.6	32	20.9	275	1205.2	896.9	0.74	108.4524	28.2517	712	5.17	1.44	0.46	20	1638	Planted
711	**SCLF**	12.71	74.01	21.17	14.1	32	20.3	311	1184.5	835.0	0.70	105.8704	28.3585	1167	5.57	0.98	0.34	22	2243	Planted
712	**SCLF**	6.12	66.35	17.30	16.1	33	25.9	316	1453.1	899.8	0.62	114.5093	28.4017	500	5.63	0.67	0.36	22	3103	Planted
713	**SCLF**	7.50	40.46	12.01	13.2	32	18.6	308	1138.3	842.0	0.74	107.5499	28.4755	1060	5.06	1.01	0.6	17	1401	Planted
714	**SCLF**	5.29	26.69	8.17	12.6	30	17.8	297	1119.2	818.1	0.73	107.1899	28.6566	1200	6.16	1.41	0.24	18	1037	Second
715	**SCLF**	14.85	108.12	25.04	17.0	32	25.8	286	1313.9	931.9	0.71	115.7253	28.7518	220	5.19	0.97	0.21	24	2016	Second
716	**SCLF**	9.33	51.51	15.11	12.5	32	22.2	270	1158.1	826.4	0.71	107.9204	28.874	1100	5.55	1.27	0.31	20	1713	Planted
717	**SCLF**	11.93	103.03	21.27	16.5	31	22.1	295	1253.3	913.7	0.73	111.1378	28.9092	450	5.96	1.46	0.61	21	3783	Planted
718	**SCLF**	15.76	102.51	31.08	15.7	35	22.4	262	1354.7	888.0	0.66	113.8002	29.209	500	5.05	1.53	0.2	24	1354	Second
719	**SCLF**	5.78	28.93	9.85	9.4	33	17.7	299	1588.9	679.4	0.43	109.1049	29.605	1910	5.96	1.21	0.33	22	1702	Planted
720	**SCLF**	5.49	124.7	18.00	14.9	31	23.3	304	1418.5	866.7	0.61	117.708	29.8099	450	5.26	1.4	0.31	50	3360	Planted
721	**SCLF**	23.40	83.07	22.50	14.6	34	19.9	316	923.9	811.7	0.88	103.3726	29.9279	1200	6.09	1.44	0.61	17	2005	Planted
722	**SCLF**	15.15	93.42	29.03	15.1	31	23.5	289	1115.9	873.5	0.78	109.4041	30.2019	600	5.9	1.57	0.32	23	1668	Planted
723	**SCLF**	15.28	88.81	28.34	14.1	34	20.0	312	1269.4	845.4	0.67	115.7002	31.0038	500	4.95	0.94	0.31	21	2217	Planted
724	**SCLF**	9.38	51.35	16.82	14.3	33	21.4	279	1219.6	852.8	0.70	116.3091	31.3042	400	5.56	0.8	0.33	20	1800	Planted
725	**SCLF**	12.25	63.71	21.34	13.8	31	21.6	270	1336.7	836.7	0.63	119.8045	31.3036	400	5.43	1.12	0.61	20	3000	Planted
726	**SCLF**	6.58	30.77	10.79	14.2	32	21.1	288	1177.5	849.4	0.72	115.8081	31.6054	400	5.27	1.35	0.48	20	2700	Planted
727	**SCLF**	12.10	60.84	19.11	15.3	34	23.5	301	1123.8	886.9	0.79	119.5161	31.6454	50	5.9	1.44	0.69	16	3765	Planted
728	**SCLF**	12.23	66.25	21.84	15.0	33	21.2	287	996.6	879.1	0.88	114.0883	32.1268	200	5.89	1.01	0.45	23	2750	Planted
729	**SCLF**	5.00	40.5	10.39	21.7	33	31.6	304	1776.1	1055.3	0.59	109.5027	22.2091	400	6.13	1.37	0.43	18	2408	Second
730	**SCLF**	5.78	70.46	17.19	21.4	34	29.0	291	1747.6	1049.8	0.60	114.706	22.9066	200	5.33	0.82	0.29	21	2550	Planted
731	**SCLF**	5.10	75.48	16.25	20.7	33	27.3	296	1778.2	1027.0	0.58	111.8834	23.128	400	5.6	1.03	0.64	21	2520	Planted
732	**SCLF**	6.65	61.1	14.87	20.0	33	28.6	294	1673.8	1010.3	0.60	111.5077	24.107	400	4.92	0.43	0.44	20	1860	Planted
733	**SCLF**	10.12	100.91	19.87	19.2	32	27.8	319	1592.8	980.1	0.62	106.3356	24.2896	720	6.03	1.18	0.35	24	1500	Second
734	**SCLF**	6.47	67.28	17.65	19.5	30	27.9	269	1758.7	993.3	0.56	114.4068	24.3023	400	5.19	1.17	0.42	16	3015	Planted
735	**SCLF**	9.18	81.14	20.85	16.0	34	23.2	295	1854.9	874.9	0.47	106.3118	24.4044	1373	5.81	0.51	0.38	18	2304	Second
736	**SCLF**	12.97	142.61	35.31	18.2	33	26.9	275	1860.9	953.7	0.51	114.5084	24.7041	600	5.37	1.48	0.66	29	1905	Planted
737	**SCLF**	12.97	142.61	35.31	18.2	35	25.3	303	1847.2	952.3	0.52	114.4064	24.8063	600	5.94	0.87	0.24	29	1905	Planted
738	**SCLF**	5.13	66.65	13.22	19.1	33	26.9	276	1668.8	981.4	0.59	111.5092	24.8022	500	5.86	1.02	0.44	26	1645	Planted
739	**SCLF**	6.67	90.92	18.13	19.3	31	25.0	278	1631.3	988.2	0.61	111.7054	24.9086	439	5.15	0.66	0.25	23	2005	Planted
740	**SCLF**	9.60	98.35	22.84	19.8	31	27.6	265	1504.3	1003.9	0.67	109.2038	25.0025	400	5.57	0.82	0.21	24	1640	Second
741	**SCLF**	6.23	80.72	15.66	19.7	30	29.3	291	1565.4	1001.5	0.64	111.7075	25.0076	343	5.94	0.58	0.32	29	1681	Planted

742	**SCLF**	4.53	93.24	18.86	18.6	33	24.6	265	1710.8	967.5	0.57	113.3004	25.1003	500	5.98	0.88	0.65	25	1463	Planted
743	**SCLF**	4.80	62.57	12.70	19.8	34	27.0	267	1510.0	1006.0	0.67	111.3093	25.2063	302	5.29	0.67	0.46	22	2122	Planted
744	**SCLF**	5.02	142.73	37.20	15.9	33	22.8	271	2039.8	882.4	0.43	116.9074	25.6862	865	6.19	0.45	0.46	53	1530	Planted
745	**SCLF**	8.83	95.68	24.36	17.8	35	23.8	268	1701.2	945.2	0.56	117.5098	26.1061	400	5.73	0.73	0.25	17	2690	Planted
746	**SCLF**	13.55	183.82	43.15	17.4	34	22.5	296	1685.5	934.9	0.55	118.1046	26.5058	400	4.95	0.72	0.48	27	2075	Planted
747	**SCLF**	19.56	194.52	42.24	16.5	34	23.4	266	1298.8	968.9	0.75	109.1022	26.6022	434	5.34	1.14	0.45	24	2464	Second
748	**SCLF**	19.09	246.3	46.18	18.3	34	23.5	290	1389.1	962.2	0.69	109.6086	26.7062	450	4.86	1.38	0.54	24	2951	Second
749	**SCLF**	11.03	61.13	17.90	16.2	32	23.8	287	1346.6	966.3	0.72	109.3583	26.7572	425	5.28	1.3	0.42	18	2017	Second
750	**SCLF**	6.45	160.29	35.39	17.1	34	24.7	267	1667.7	927.9	0.56	118.3027	26.8071	403	6.16	0.75	0.47	32	1213	Planted
751	**SCLF**	10.14	397.41	82.66	18.3	30	23.7	299	1360.9	966.0	0.71	109.5038	26.809	417	5.27	0.77	0.41	38	2263	Planted
752	**SCLF**	11.18	132.24	28.50	18.1	33	27.0	309	1527.4	960.1	0.63	117.9262	26.8021	210	6.09	0.62	0.69	18	3418	Second
753	**SCLF**	9.11	185.5	12.59	18.4	32	24.8	280	1360.5	966.9	0.71	109.7557	26.8351	400	6.2	0.87	0.38	31	1868	Planted
754	**SCLF**	11.26	135.05	33.18	16.9	34	23.0	314	1649.4	921.3	0.56	118.6015	27.1067	400	5.31	1.23	0.46	19	2435	Planted
755	**SCLF**	9.20	248.4	50.51	15.3	34	23.8	269	1773.9	870.5	0.49	117.6061	27.5052	703	5.26	0.93	0.52	35	1584	Planted
756	**SCLF**	10.13	73.18	19.57	17.1	33	23.7	279	1463.7	928.3	0.63	114.6012	27.7097	400	4.92	0.75	0.59	19	3611	Planted
757	**SCLF**	11.66	71.1	22.45	11.2	33	19.8	296	1515.4	750.6	0.50	116.2007	31.3061	1050	5.37	0.97	0.49	23	2280	Planted
758	**SCLF**	9.28	61.87	19.85	14.8	33	22.9	292	870.2	821.8	0.94	109.3095	32.3009	680	5.76	1.49	0.63	28	1532	Second
759	**SCLF**	15.79	45.79	17.24	14.7	34	21.6	280	1109.3	871.0	0.79	121.1868	32.3318	20	5.27	0.62	0.22	20	1665	Planted
760	**SCLF**	6.06	91.68	25.34	15.9	32	21.5	314	1229.3	904.2	0.74	120.5025	30.4885	50	5.92	1.44	0.62	18	2490	Second
761	**SCLF**	4.73	104.13	21.01	19.6	34	29.1	313	1778.3	995.6	0.56	112.1085	23.9043	500	5.43	1.41	0.24	18	1478	Second
762	**SCLF**	6.34	58.36	14.19	21.6	35	31.2	277	1734.0	1053.5	0.61	109.9926	22.5588	355	5.39	0.9	0.64	22	1702	Planted
763	**SEBF**	9.33	143.59	31.91	18.2	34	27.3	311	1688.4	956.3	0.57	116.3081	25.8053	400	5.55	1.58	0.28	45	1515	Planted
764	**SEBF**	19.27	377.64	73.74	17.6	33	25.4	322	1766.8	938.8	0.53	119.3014	26.0015	400	5.38	0.45	0.66	71	533	Primary
765	**SEBF**	18.91	319.07	54.38	18.8	35	26.4	251	1815.9	972.9	0.54	117.2017	24.705	400	5.47	1.34	0.35	47	1580	Planted
766	**SEBF**	9.93	153.65	38.07	14.8	37	24.2	226	1952.0	851.3	0.44	118.1008	27.0009	870	6.76	1.07	0.68	20	2300	Planted
767	**SEBF**	19.40	317.17	55.34	17.1	33	26.8	295	1620.3	926.8	0.57	118.1078	27.1037	380	5.31	1.14	0.34	120	1175	Primary
768	**SEBF**	14.92	229.66	53.21	16.3	37	23.3	256	1895.9	896.5	0.47	118.7029	26.3057	650	6.45	0.58	0.35	50	2850	Planted
769	**SEBF**	22.70	456.08	86.48	15.1	38	20.3	314	1972.8	859.0	0.44	118.0037	26.7022	860	6.84	1.46	0.37	200	150	Planted
770	**SEBF**	7.92	145.78	46.79	14.0	38	19.3	323	2149.2	822.5	0.38	118.1093	26.602	1100	6.86	1.3	0.49	40	5400	Primary
771	**SEBF**	9.85	156.42	39.19	15.9	36	24.6	241	1699.6	888.7	0.52	118.507	27.6056	550	6.39	0.83	0.24	29	1833	Second
772	**SEBF**	22.65	446.65	80.88	17.7	34	22.9	263	1713.4	942.0	0.55	117.4065	26.1079	423	5.46	0.52	0.28	99	625	Primary
773	**SEBF**	10.83	173.02	35.89	17.0	36	26.6	325	2009.7	915.7	0.46	116.4037	25.0012	750	6.48	0.81	0.35	45	1196	Primary
774	**SEBF**	14.15	251.86	48.50	15.4	34	23.5	260	1814.3	871.1	0.48	117.1062	27.2057	750	5.84	0.49	0.34	40	1203	Primary
775	**SEBF**	19.78	306.28	66.75	15.2	35	24.3	286	1871.3	863.9	0.46	117.6062	27.1093	793	5.19	0.58	0.36	55	2675	Planted
776	**SEBF**	22.81	410.52	70.86	16.8	36	25.1	227	1617.3	917.1	0.57	117.4095	27.3026	438	5.06	1.55	0.52	115	417	Primary
777	**SEBF**	21.74	403.57	70.35	17.1	38	24.6	312	1635.7	925.4	0.57	117.6032	27.0038	415	5.07	0.6	0.3	80	433	Primary
778	**SEBF**	15.69	248.31	50.62	18.2	37	24.1	268	1816.7	954.2	0.53	117.5096	25.2085	450	4.89	1.26	0.7	50	1500	Planted
779	**SEBF**	12.47	279.55	133.45	21.2	35	29.7	280	1713.3	1044.2	0.61	112.5858	23.1377	270	5.04	0.44	0.37	200	672	Planted
780	**SEBF**	16.89	141.16	48.30	24.2	38	32.6	240	1714.0	1131.7	0.66	110.0084	20.7022	80	6.47	0.85	0.26	22	1050	Second
781	**SEBF**	14.88	266.3	72.34	20.5	38	29.8	275	1734.5	1024.0	0.59	111.8859	23.4565	380	5.86	1.44	0.59	100	3739	Primary
782	**SEBF**	9.07	152.05	28.89	20.3	38	29.0	249	1927.4	1014.2	0.53	115.6023	22.9028	400	5.17	0.57	0.46	25	525	Second
783	**SEBF**	7.67	117.93	26.42	18.6	35	25.2	267	1756.0	968.0	0.55	114.0078	24.9074	500	5.33	0.79	0.32	25	1457	Second
784	**SEBF**	6.03	180.54	35.59	19.2	38	25.8	312	1638.6	983.9	0.60	110.8224	24.8356	500	5.75	0.47	0.65	35	2200	Primary
785	**SEBF**	12.63	216.11	44.16	16.9	37	22.2	268	1763.9	909.7	0.52	108.6285	25.106	1002	6.28	1.35	0.27	51	735	Planted

786	SEBF	10.94	181.61	38.29	17.3	33	26.6	249	1710.4	925.6	0.54	109.9714	25.4588	820	4.87	1.09	0.5	35	800	Primary
787	SEBF	10.27	159.86	37.10	16.1	34	23.9	275	1845.0	884.0	0.48	109.9393	25.4525	1085	5.95	0.7	0.47	40	1487	Planted
788	SEBF	10.44	175.65	34.37	13.1	36	22.0	261	2135.2	787.0	0.37	109.9822	25.6042	1680	6.33	1.5	0.55	60	620	Planted
789	SEBF	6.94	113.12	22.15	18.7	36	24.1	236	1626.5	970.4	0.60	110.6415	25.1741	550	4.92	0.9	0.68	30	499	Planted
790	SEBF	10.41	169.37	36.49	17.1	35	26.3	264	1710.9	917.7	0.54	110.2861	25.6764	835	6.34	0.78	0.3	32	898	Planted
791	SEBF	3.75	63.96	18.79	18.7	35	27.3	273	1483.4	972.8	0.66	110.2809	25.9751	450	6.54	1.01	0.44	29	1882	Planted
792	SEBF	12.41	198.12	46.30	18.5	35	26.9	269	1507.8	967.1	0.64	110.1063	25.9081	500	6.8	0.7	0.5	31	3272	Planted
793	SEBF	11.66	88.44	28.00	19.4	37	28.1	256	1763.5	987.3	0.56	108.3652	23.573	700	5.57	1.44	0.59	20	1906	Planted
794	SEBF	11.67	205.4	34.91	18.2	33	27.5	258	1667.6	952.7	0.57	109.6307	25.109	700	5.51	1.42	0.39	44	439	Primary
795	SEBF	21.17	155.41	47.93	18.6	36	27.2	236	2176.9	950.1	0.44	107.711	21.852	1160	6.01	0.99	0.61	41	4404	Primary
796	SEBF	8.23	152.2	65.31	15.1	37	21.3	289	1925.4	845.8	0.44	106.3404	24.4548	1550	6.61	1.08	0.56	44	294	Planted
797	SEBF	6.87	137.41	37.99	13.0	34	22.7	284	2101.4	776.7	0.37	106.4021	24.5254	1978	5.24	0.45	0.22	53	504	Planted
798	SEBF	5.19	93.38	22.13	17.7	33	22.9	302	1623.6	929.9	0.57	104.4172	24.236	1100	6.6	0.84	0.3	24	1085	Second
799	SEBF	4.32	86.55	17.29	17.0	34	27.0	236	1626.4	910.7	0.56	104.6845	24.5876	1172	5.09	1.24	0.6	20	845	Planted
800	SEBF	4.37	72.61	20.61	20.0	38	26.8	326	1467.0	1012.5	0.69	109.936	25.1771	305	6.56	1.32	0.49	17	1913	Second
801	SEBF	11.87	199.06	35.55	19.1	34	27.0	260	1525.9	983.5	0.64	109.7537	25.3853	468	6.16	0.48	0.45	30	627	Second
802	SEBF	9.72	176.23	30.92	18.4	38	27.0	307	1519.2	962.9	0.63	110.605	26.006	500	6.76	0.78	0.37	42	585	Planted
803	SEBF	17.75	313.32	57.01	18.4	35	24.2	247	1553.1	961.8	0.62	110.0072	25.7069	560	6.79	0.42	0.28	81	1087	Primary
804	SEBF	2.82	34.37	10.20	17.4	33	24.8	292	1622.5	931.3	0.57	110.6074	26.0017	700	5.03	1.24	0.54	3	1680	Second
805	SEBF	5.65	110.18	22.91	17.1	33	23.9	292	1317.6	934.1	0.71	106.0027	24.757	965	6.59	0.49	0.6	25	1134	Second
806	SEBF	16.57	270.38	232.53	19.5	33	29.4	277	1290.2	997.4	0.77	105.802	25.0002	540	5.68	1.14	0.29	78	680	Primary
807	SEBF	7.06	125.02	25.44	12.0	34	19.8	300	1174.0	776.2	0.66	105.874	28.3257	1543	5.23	1.14	0.65	70	2718	Primary
808	SEBF	4.92	92.56	18.06	12.9	37	19.1	261	1197.0	799.6	0.67	105.7044	28.3269	1400	5.07	1.2	0.3	50	1538	Planted
809	SEBF	4.36	79.64	14.95	14.7	35	23.8	295	1192.5	850.0	0.71	105.8309	28.3554	1073	5.87	1.12	0.49	51	1874	Planted
810	SEBF	4.22	71.65	14.00	15.8	37	23.2	274	1151.5	888.0	0.77	106.2018	28.5346	800	5.88	1.5	0.21	35	2405	Planted
811	SEBF	6.67	126.75	22.83	15.5	37	24.7	267	1150.6	879.7	0.76	106.1666	28.6526	840	4.99	1.06	0.39	30	2646	Planted
812	SEBF	6.23	114.89	21.05	15.8	37	21.5	330	1295.1	942.7	0.73	108.4598	25.506	750	5.54	0.63	0.33	30	2765	Planted
813	SEBF	4.97	92.76	14.80	13.3	38	22.1	320	1386.6	870.7	0.63	108.3391	25.5852	1200	6.44	1.14	0.22	20	3545	Second
814	SEBF	4.99	101.44	20.28	12.2	37	18.5	300	1426.5	838.6	0.59	108.2998	25.628	1400	5.63	1.11	0.61	50	885	Planted
815	SEBF	6.69	109.15	27.14	16.9	37	23.8	266	1178.3	961.7	0.82	107.9762	26.2006	560	5.52	0.42	0.51	40	2004	Planted
816	SEBF	6.88	110.4	20.74	15.0	34	21.9	314	1245.3	908.6	0.73	108.0059	26.2314	892	5.74	0.72	0.59	35	6378	Planted
817	SEBF	4.81	83.71	16.11	14.0	36	20.8	325	1272.3	878.5	0.69	107.9361	26.275	1080	5.03	0.83	0.23	22	2515	Planted
818	SEBF	7.18	121.58	26.97	14.9	36	20.8	261	1241.1	906.1	0.73	107.9799	26.3084	900	6.78	1.12	0.56	40	2623	Planted
819	SEBF	3.56	66.23	11.20	10.9	34	16.3	286	1112.1	772.3	0.69	107.4461	29.2296	1415	5.71	1.03	0.4	30	2033	Planted
820	SEBF	6.17	99.46	22.39	11.7	33	19.5	265	1159.0	802.5	0.69	107.7101	28.6827	1280	5.81	0.49	0.47	20	2797	Planted
821	SEBF	7.06	133.75	31.44	12.9	35	18.2	307	1312.7	845.5	0.64	107.6815	25.9064	1340	5.83	1.02	0.26	31	1131	Planted
822	SEBF	3.56	68.06	16.69	11.7	36	21.4	299	1439.8	748.2	0.52	104.5702	27.139	1900	6.52	0.55	0.49	22	478	Planted
823	SEBF	9.95	196.09	34.19	13.2	36	20.5	223	1229.0	849.7	0.69	107.6386	26.9548	1190	6.64	1.14	0.34	41	3516	Primary
824	SEBF	4.11	77.62	15.92	14.5	38	20.1	326	1308.5	905.4	0.69	108.557	26.4542	870	5.67	1.3	0.41	30	1055	Second
825	SEBF	13.93	268.39	46.37	15.2	35	22.8	327	1316.0	929.6	0.71	108.8369	26.5225	700	6.69	1.06	0.69	39	5897	Second
826	SEBF	10.27	195.83	74.41	15.2	35	22.0	224	1288.3	926.2	0.72	108.7154	26.7342	700	4.81	0.75	0.5	65	423	Primary
827	SEBF	14.67	272.99	45.34	13.8	34	21.4	259	1482.9	901.2	0.61	109.3984	26.5554	860	6.69	1.34	0.66	50	9057	Planted
828	SEBF	5.94	106.31	21.10	14.5	37	22.8	270	1243.3	894.1	0.72	107.9878	26.5273	950	5.91	1.36	0.63	46	2371	Primary
829	SEBF	8.36	160.55	31.20	10.8	34	19.7	237	1404.6	794.2	0.57	108.1624	26.3374	1600	5.06	0.96	0.23	35	2347	Planted

830	SEBF	6. 16	111. 74	22. 88	11. 6	34	18. 0	330	1383. 4	818. 8	0.59	108. 273	26. 4277	1430	5. 6	1. 58	0. 46	20	2011	Planted
831	SEBF	5. 75	108. 65	22. 15	9. 1	35	16. 0	281	1458. 0	748. 5	0.51	108. 2311	26. 5289	1865	5. 76	0. 46	0. 4	35	1505	Planted
832	SEBF	8. 74	154. 22	49. 01	14. 6	33	21. 3	274	1267. 6	900. 8	0.71	108. 2034	26. 4093	915	6. 39	0. 61	0. 25	43	1196	Planted
833	SEBF	7. 77	151. 72	41. 46	9. 9	38	17. 7	255	1415. 7	766. 3	0.54	108. 0021	26. 3007	1785	6. 86	0. 76	0. 55	80	882	Primary
834	SEBF	4. 68	89. 73	16. 77	16. 7	35	23. 5	268	1292. 0	973. 1	0.75	108. 9296	25. 9581	490	5. 49	0. 74	0. 54	26	1523	Planted
835	SEBF	4. 25	77. 26	15. 16	15. 0	37	24. 1	229	1379. 7	927. 1	0.67	108. 9601	25. 9565	780	6. 12	1. 35	0. 38	25	1598	Planted
836	SEBF	8. 24	152. 22	38. 52	14. 5	37	22. 6	244	1475. 5	920. 8	0.62	109. 3677	26. 0273	800	6. 14	0. 45	0. 56	50	1212	Planted
837	SEBF	5. 17	96. 23	17. 30	14. 5	34	19. 8	270	1477. 7	922. 7	0.62	109. 4029	26. 0803	780	4. 96	0. 69	0. 36	30	2356	Planted
838	SEBF	4. 52	79. 66	18. 28	15. 2	35	24. 8	278	1411. 9	938. 3	0.66	109. 2849	26. 2063	670	6. 11	1. 43	0. 56	30	1169	Planted
839	SEBF	11. 35	211. 52	37. 33	14. 7	37	22. 3	234	1443. 9	925. 0	0.64	109. 3244	26. 2396	750	6. 68	0. 97	0. 3	25	5462	Planted
840	SEBF	6. 42	118. 82	25. 41	16. 3	33	26. 0	226	1241. 8	943. 3	0.76	107. 7911	25. 2127	800	4. 92	1. 59	0. 23	31	1600	Planted
841	SEBF	7. 01	124. 6	27. 30	16. 2	33	22. 9	283	1251. 9	942. 7	0.75	107. 9149	25. 2114	800	5. 56	0. 67	0. 55	50	2033	Planted
842	SEBF	6. 49	111. 11	22. 16	16. 0	35	21. 1	312	1259. 2	940. 2	0.75	107. 952	25. 2169	815	6. 21	1. 34	0. 29	45	3277	Planted
843	SEBF	7. 59	136. 43	28. 49	16. 4	36	23. 1	249	1247. 7	950. 3	0.76	107. 994	25. 2101	750	6. 72	0. 82	0. 3	55	2447	Planted
844	SEBF	6. 17	108. 86	21. 00	16. 2	36	24. 7	234	1252. 0	943. 4	0.75	107. 9513	25. 2584	790	6. 74	0. 83	0. 49	20	2953	Planted
845	SEBF	7. 65	134. 14	28. 61	15. 8	36	21. 6	307	1270. 1	933. 7	0.74	107. 9973	25. 2567	850	6. 41	0. 47	0. 53	45	2637	Planted
846	SEBF	3. 82	66. 57	14. 52	15. 6	37	20. 9	320	1268. 0	925. 4	0.73	107. 8796	25. 3006	900	6. 67	0. 65	0. 45	30	1257	Planted
847	SEBF	6. 20	109. 4	22. 37	15. 2	38	22. 2	299	1282. 7	916. 3	0.71	107. 9154	25. 3398	953	6. 67	0. 43	0. 52	30	2408	Planted
848	SEBF	4. 48	75. 43	13. 71	17. 1	36	22. 9	299	1214. 5	970. 6	0.80	108. 0618	25. 38	600	5. 42	1. 42	0. 3	50	3555	Planted
849	SEBF	3. 57	60. 05	11. 87	16. 7	36	25. 5	300	1234. 4	962. 3	0.78	108. 1477	25. 3749	650	6. 71	0. 61	0. 35	30	2079	Planted
850	SEBF	4. 65	94. 15	16. 37	16. 5	36	22. 2	241	1230. 4	954. 6	0.78	107. 9999	25. 4087	700	5. 06	0. 77	0. 32	50	1395	Planted
851	SEBF	3. 40	56. 18	10. 63	16. 2	37	22. 8	300	1247. 2	945. 0	0.76	108. 0207	25. 4005	760	5. 49	1. 4	0. 38	20	2595	Planted
852	SEBF	5. 55	108. 52	18. 31	15. 3	37	25. 2	242	1261. 2	912. 8	0.72	107. 6789	25. 4332	970	6. 13	1. 09	0. 57	46	2348	Primary
853	SEBF	5. 15	96. 13	17. 79	15. 6	35	23. 9	246	1280. 6	929. 6	0.73	108. 142	25. 4364	850	4. 93	0. 64	0. 48	50	2063	Planted
854	SEBF	6. 73	123. 06	23. 11	16. 7	38	22. 8	260	1212. 0	954. 2	0.79	107. 7936	25. 4793	700	5. 02	1. 41	0. 21	50	2896	Planted
855	SEBF	5. 65	95. 81	19. 15	15. 8	36	24. 3	221	1261. 1	936. 0	0.74	108. 1	25. 5295	800	6. 81	1. 15	0. 54	40	3002	Planted
856	SEBF	4. 65	80. 78	16. 08	15. 8	34	24. 0	273	1265. 7	935. 8	0.74	108. 1475	25. 5236	800	6. 3	0. 79	0. 57	50	2162	Planted
857	SEBF	3. 35	53. 56	10. 55	12. 7	34	18. 2	274	1314. 7	829. 8	0.63	107. 0491	25. 5569	1500	5. 08	0. 53	0. 42	36	2613	Planted
858	SEBF	7. 70	125. 71	25. 63	14. 9	36	22. 5	235	1281. 3	904. 9	0.71	107. 886	25. 5524	1000	6. 26	1. 26	0. 37	30	4695	Planted
859	SEBF	3. 66	67. 23	14. 02	16. 8	35	23. 2	309	1208. 9	960. 0	0.79	107. 9245	25. 5577	650	5. 92	1. 46	0. 61	30	1037	Planted
860	SEBF	8. 78	151. 51	37. 67	17. 4	37	26. 5	299	1187. 9	977. 1	0.82	107. 9642	25. 551	540	5. 34	0. 54	0. 45	46	1958	Primary
861	SEBF	3. 63	60. 32	12. 35	15. 6	35	22. 0	279	1265. 0	927. 2	0.73	108. 0509	25. 589	850	5. 52	1. 39	0. 69	45	1958	Planted
862	SEBF	5. 31	91. 82	22. 45	16. 4	34	26. 0	285	1235. 1	950. 7	0.77	108. 0952	25. 5816	700	6. 2	1. 48	0. 3	38	1242	Planted
863	SEBF	8. 16	160. 37	44. 59	12. 6	38	22. 3	240	1257. 3	820. 7	0.65	106. 9758	26. 3767	1462	4. 96	1. 24	0. 61	46	649	Primary
864	SEBF	5. 13	98. 2	22. 48	17. 3	35	25. 1	265	1194. 6	957. 1	0.80	106. 9651	25. 4376	710	5. 8	1. 56	0. 21	21	848	Second
865	SEBF	5. 52	97. 48	21. 32	14. 7	35	22. 2	258	1230. 6	885. 3	0.72	107. 1304	26. 0279	1090	6. 87	0. 61	0. 48	35	1668	Primary
866	SEBF	4. 54	84. 67	15. 30	16. 0	34	24. 7	298	1238. 0	939. 2	0.76	108. 051	25. 7563	754	5. 88	1. 31	0. 56	20	1990	Second
867	SEBF	5. 85	109. 63	21. 77	14. 2	36	19. 8	270	1296. 6	889. 9	0.69	108. 1287	26. 029	1032	5. 18	0. 7	0. 51	25	1767	Second
868	SEBF	8. 17	153. 78	40. 22	16. 5	34	25. 2	272	1217. 1	954. 6	0.78	108. 205	26. 0275	620	5. 58	0. 98	0. 38	40	981	Primary
869	SEBF	9. 75	169. 65	42. 47	15. 6	34	24. 2	257	1229. 3	925. 8	0.75	107. 9304	26. 0216	810	6. 41	0. 41	0. 47	46	2039	Planted
870	SEBF	5. 20	84. 88	18. 45	14. 1	33	20. 3	253	1226. 0	881. 5	0.72	108. 0536	27. 172	950	6. 39	0. 55	0. 2	25	2502	Second
871	SEBF	5. 90	113. 46	29. 66	13. 4	35	20. 5	302	1273. 2	868. 2	0.68	108. 4577	27. 5393	980	5. 81	1. 3	0. 4	50	633	Planted
872	SEBF	5. 60	109. 32	23. 62	13. 2	38	19. 3	313	1389. 7	873. 7	0.63	109. 2555	28. 1349	850	5. 19	0. 94	0. 7	22	986	Second
873	SEBF	6. 50	131. 9	22. 40	10. 4	37	15. 5	268	1421. 8	786. 4	0.55	108. 3112	26. 5555	1619	6. 4	1. 25	0. 61	25	2074	Second

874	**SEBF**	7.54	129.5	31.39	15.8	38	23.7	301	1367.5	954.9	0.70	109.3585	26.7304	500	6.42	1.42	0.68	30	1869	Second
875	**SEBF**	11.60	226.08	75.66	14.8	33	24.2	254	1336.9	917.8	0.69	109.0268	27.1012	700	5.44	1.6	0.21	110	588	Primary
876	**SEBF**	7.71	138.46	37.30	15.8	38	22.3	236	1326.6	949.3	0.72	109.3425	27.7039	420	6.31	1.08	0.36	29	1088	Second
877	**SEBF**	5.54	109.09	24.35	17.3	34	24.3	230	1284.4	940.9	0.73	106.1263	25.0326	885	5.9	0.58	0.27	25	837	Second
878	**SEBF**	5.42	96.7	37.65	18.3	36	27.4	278	1245.7	972.0	0.78	106.2474	25.0751	680	5.44	1.01	0.45	45	292	Primary
879	**SEBF**	5.49	99.87	17.75	16.1	34	22.2	268	1150.1	921.2	0.80	107.2366	27.027	740	6.86	1.11	0.27	16	2993	Planted
880	**SEBF**	5.83	109.28	20.45	14.1	37	23.9	248	1203.5	873.6	0.73	107.6369	27.0286	1030	4.83	1.51	0.58	30	2171	Planted
881	**SEBF**	4.48	78.47	15.08	13.1	35	23.0	259	1223.5	844.8	0.69	107.5943	27.0522	1210	6.46	0.97	0.38	24	2267	Second
882	**SEBF**	4.36	80.73	14.70	14.0	37	19.2	222	1202.0	871.4	0.72	107.6743	27.1362	1030	5.99	0.47	0.57	23	1954	Planted
883	**SEBF**	13.21	252.7	74.58	12.2	36	21.5	290	1212.4	802.2	0.66	106.7058	27.1002	1500	6.39	0.67	0.51	37	1037	Planted
884	**SEBF**	3.61	71.52	12.91	14.2	38	23.6	310	1168.8	841.7	0.72	106.0307	28.3514	1120	5.42	0.57	0.23	40	1076	Planted
885	**SEBF**	5.14	83.74	21.59	13.9	35	20.4	279	1165.0	831.3	0.71	106.031	28.4085	1180	6.46	1.46	0.63	41	1376	Primary
886	**SEBF**	5.07	97.21	18.17	13.0	35	19.3	227	1158.2	809.0	0.70	106.0754	28.4064	1320	5.82	1.4	0.58	31	1650	Planted
887	**SEBF**	3.99	80.32	13.97	14.3	38	24.1	231	1161.7	843.2	0.73	106.07	28.4395	1100	5.79	1.01	0.29	40	1242	Planted
888	**SEBF**	6.20	99.83	21.11	12.6	38	17.7	245	1146.6	798.4	0.70	106.1954	28.4364	1380	4.95	0.78	0.54	43	3556	Planted
889	**SEBF**	6.55	124.33	26.40	12.5	38	22.4	258	1135.5	799.7	0.70	106.3239	28.5054	1360	5.76	1.11	0.7	80	1440	Primary
890	**SEBF**	7.47	145.78	31.51	14.7	35	21.7	247	1144.4	857.9	0.75	106.2495	28.5336	990	6.67	0.87	0.3	70	1315	Primary
891	**SEBF**	5.46	104.69	19.57	13.9	36	19.8	289	1139.3	837.2	0.73	106.2862	28.5386	1120	5.76	0.57	0.66	70	1777	Primary
892	**SEBF**	5.46	104.69	19.57	13.3	35	21.6	306	1129.1	821.6	0.73	106.4004	28.5709	1210	6.36	0.61	0.32	41	1777	Primary
893	**SEBF**	3.34	67.03	10.04	11.8	36	19.9	235	1124.7	781.8	0.70	106.4875	28.5712	1460	6.39	1.12	0.55	30	1896	Planted
894	**SEBF**	7.38	141.42	33.97	16.4	38	24.1	281	1541.8	886.7	0.58	104.6116	25.7577	1186	5.41	1.35	0.38	40	1039	Planted
895	**SEBF**	6.37	120.56	25.56	14.4	36	20.8	320	1206.4	892.6	0.74	108.4395	28.2534	740	4.93	1.46	0.62	32	1434	Planted
896	**SEBF**	5.34	90.73	18.92	14.5	36	23.2	267	1154.6	887.9	0.77	108.1472	28.6826	727	5.51	0.69	0.67	38	2396	Planted
897	**SEBF**	4.78	78.27	15.78	14.2	35	23.9	229	1155.3	877.0	0.76	108.1988	28.9757	760	6.12	0.57	0.3	31	3645	Planted
898	**SEBF**	4.68	79.34	15.37	12.8	35	19.6	309	1290.0	851.3	0.66	108.6022	27.9782	1030	5.46	0.64	0.56	22	2809	Planted
899	**SEBF**	3.41	72.86	10.14	13.6	33	21.1	277	1269.6	870.9	0.69	108.3034	27.1706	1010	5.48	1.5	0.35	20	1651	Planted
900	**SEBF**	6.17	116.78	25.57	15.3	34	20.6	257	1282.1	889.4	0.69	106.3666	25.4052	1160	5.94	1.54	0.65	21	1255	Planted
901	**SEBF**	9.54	175.39	49.40	12.6	36	20.7	301	1309.4	845.7	0.65	108.6511	27.9002	1072	5.84	1.17	0.39	61	2384	Primary
902	**SEBF**	6.12	107	24.68	15.4	36	23.7	250	1267.8	923.1	0.73	108.1358	25.7758	851	6.21	1.34	0.48	25	1630	Second
903	**SEBF**	4.27	81.77	15.28	14.9	37	24.5	302	1323.3	916.8	0.69	108.5361	25.9067	864	6.45	0.51	0.45	20	1388	Second
904	**SEBF**	4.91	89.7	19.64	16.3	35	25.6	234	1238.0	950.8	0.77	108.3278	25.9883	645	6.38	0.41	0.21	45	1215	Primary
905	**SEBF**	8.02	140.63	36.02	12.3	36	17.4	270	1433.4	845.8	0.59	108.6022	26.0871	1290	5.52	1.17	0.56	70	1495	Primary
906	**SEBF**	4.21	82.69	16.03	16.2	38	25.9	291	1240.9	951.2	0.77	108.4882	26.3	600	5.41	0.8	0.21	40	1029	Primary
907	**SEBF**	4.98	87.74	17.69	15.5	34	22.0	226	1261.0	931.6	0.74	108.4781	26.4251	710	5.46	0.76	0.65	20	2032	Second
908	**SEBF**	6.24	103.86	29.77	14.1	38	21.3	248	1925.3	821.0	0.43	110.1672	26.1822	1390	5.27	0.97	0.42	22	2784	Planted
909	**SEBF**	8.79	135.95	34.08	17.5	34	25.1	257	1543.4	934.5	0.61	110.2084	26.3502	650	5.5	0.5	0.62	34	1834	Planted
910	**SEBF**	9.43	146.41	37.51	16.2	33	23.0	242	1651.3	894.7	0.54	110.5679	26.532	870	6.67	1.48	0.46	59	1857	Planted
911	**SEBF**	17.66	334.05	63.11	15.2	35	20.4	316	1806.5	858.3	0.48	110.209	26.2067	1150	6.67	1.13	0.54	111	671	Primary
912	**SEBF**	15.23	262.08	71.06	18.1	37	26.9	319	1383.4	959.0	0.69	109.7507	26.8378	450	6.52	1.31	0.48	80	1125	Primary
913	**SEBF**	15.09	312.78	54.52	16.2	36	22.1	225	2049.7	885.1	0.43	111.5545	24.7098	1120	6.59	0.97	0.6	90	254	Primary
914	**SEBF**	28.53	541.58	87.19	18.7	33	24.6	291	1696.9	970.7	0.57	111.7055	24.9072	550	5.64	1.42	0.34	124	978	Primary
915	**SEBF**	19.41	310.73	60.11	17.1	36	23.8	286	1858.1	915.0	0.49	110.9639	25.0225	910	5.75	1.11	0.68	55	1564	Planted
916	**SEBF**	11.37	192.69	35.56	14.8	37	21.6	239	2138.5	840.4	0.39	111.9533	25.203	1332	5.55	0.77	0.53	40	590	Planted
917	**SEBF**	10.55	163.83	40.38	17.9	36	25.3	281	1681.0	945.1	0.56	111.3681	25.5048	650	5.49	1.54	0.65	34	1821	Planted

918	SEBF	8.40	133.41	31.65	18.2	36	23.5	246	1708.4	951.1	0.56	111.3065	25.209	650	6.6	0.82	0.53	27	1353	Planted
919	SEBF	12.05	189.54	41.99	18.1	33	27.3	238	1415.6	956.2	0.68	109.516	26.6203	500	6.11	0.47	0.51	51	1412	Planted
920	SEBF	9.61	160.54	32.15	17.9	34	26.3	257	1835.2	942.0	0.51	112.8065	24.9017	700	5.54	0.97	0.63	33	900	Planted
921	SEBF	10.49	162.3	40.35	18.2	35	24.3	317	1697.8	956.2	0.56	113.785	25.5099	510	6.67	0.55	0.28	35	2389	Planted
922	SEBF	10.49	167.17	47.48	11.3	34	20.4	287	1489.0	745.5	0.50	110.1368	29.783	1440	5.03	0.8	0.66	55	3671	Planted
923	SEBF	7.31	116.69	32.80	18.6	36	26.7	222	1538.5	970.9	0.63	111.7665	25.8883	430	5.51	1	0.3	21	2577	Planted
924	SEBF	8.42	132.86	29.09	16.4	36	23.9	286	1638.4	898.6	0.55	110.1214	26.4394	870	5.02	1.41	0.47	35	942	Planted
925	SEBF	14.04	226.22	47.95	16.8	36	25.8	230	1545.7	913.6	0.59	110.1218	26.7205	740	5.01	1.35	0.49	75	1256	Primary
926	SEBF	6.49	100.63	25.74	16.6	35	22.0	305	1571.1	908.1	0.58	110.3612	26.7286	768	6.1	0.57	0.42	24	1576	Second
927	SEBF	9.07	141.6	31.92	16.2	34	25.0	227	1596.2	893.6	0.56	110.124	26.7853	860	5.8	0.9	0.51	60	1190	Planted
928	SEBF	15.69	244.44	52.59	16.0	33	25.7	281	1593.3	887.3	0.56	110.1901	26.9385	880	6.89	0.57	0.59	50	1914	Planted
929	SEBF	8.79	155.58	36.37	16.7	33	26.6	305	1202.0	920.5	0.77	110.909	29.1026	390	6.74	0.66	0.28	59	1721	Planted
930	SEBF	7.62	127.67	36.32	17.9	35	23.9	301	1493.1	947.2	0.63	109.8494	26.3559	580	6.47	1.2	0.57	22	3462	Planted
931	SEBF	9.16	158.18	37.55	14.3	33	21.4	321	1826.3	829.7	0.45	110.6081	26.7079	1260	5.78	0.78	0.65	60	741	Planted
932	SEBF	7.12	115.24	34.03	16.2	37	22.8	284	2051.0	885.5	0.43	112.8699	24.9533	1050	5.74	0.45	0.4	24	2844	Second
933	SEBF	9.67	151.67	38.96	15.9	34	22.9	311	2078.3	876.0	0.42	112.7437	24.9855	1110	5.93	0.75	0.21	29	2754	Second
934	SEBF	6.50	112.17	35.48	16.8	37	24.8	303	1958.4	907.2	0.46	112.8239	24.9888	910	6.65	0.41	0.59	26	3619	Second
935	SEBF	13.28	207.63	55.50	14.1	36	22.3	228	2323.1	816.5	0.35	112.986	24.9879	1480	6.5	1.28	0.62	55	3725	Planted
936	SEBF	9.31	146.57	38.77	16.0	34	21.5	311	1293.5	897.3	0.69	110.2697	28.7368	600	6.65	1.11	0.41	23	2804	Second
937	SEBF	7.69	119.4	28.95	16.1	38	22.7	286	1273.1	900.3	0.71	110.2631	28.8298	570	5.98	0.83	0.53	19	1865	Planted
938	SEBF	10.25	164.72	38.36	17.5	36	26.8	303	1646.8	934.5	0.57	113.3622	26.2569	570	6.25	1.29	0.32	65	1132	Primary
939	SEBF	13.11	216.19	44.92	15.9	35	21.3	279	1403.0	891.8	0.64	110.4719	28.1336	700	5.31	0.83	0.42	65	971	Primary
940	SEBF	9.37	144.45	34.49	15.7	36	21.2	294	1416.2	883.1	0.62	110.5903	28.2312	740	5.31	0.61	0.46	45	1935	Primary
941	SEBF	7.24	111.53	26.09	16.0	35	23.7	253	1384.0	893.2	0.65	110.6387	28.2739	670	6.77	0.9	0.69	35	1440	Primary
942	SEBF	6.98	113.48	33.04	16.7	36	26.3	303	1377.6	918.1	0.67	113.6913	28.389	410	6.38	0.77	0.45	28	2833	Second
943	SEBF	11.30	174.18	40.91	17.1	38	24.4	255	1697.2	923.1	0.54	113.6829	26.2544	633	6.64	0.9	0.61	55	2027	Planted
944	SEBF	11.44	177.05	44.41	17.5	35	27.0	263	1643.3	935.8	0.57	113.6424	26.2823	550	5.13	0.68	0.6	60	2287	Planted
945	SEBF	8.37	128.88	30.24	14.0	37	21.6	322	2117.6	820.2	0.39	113.7673	26.1094	1300	6.13	0.8	0.66	54	1530	Planted
946	SEBF	13.01	200.77	45.46	14.4	35	21.5	281	2043.8	832.8	0.41	113.8404	26.2548	1200	5.84	0.71	0.47	55	2030	Planted
947	SEBF	9.44	149.81	37.69	16.8	36	24.6	272	1713.5	914.0	0.53	113.6433	26.3591	680	6.21	0.56	0.41	64	1372	Primary
948	SEBF	15.63	253.69	55.05	17.3	36	25.2	257	1647.5	928.6	0.56	113.7317	26.4353	575	6.44	0.61	0.53	49	1386	Planted
949	SEBF	10.20	160.03	43.60	18.7	35	24.7	288	1460.1	977.1	0.67	114.0533	26.5076	250	6.16	1.26	0.57	60	3004	Planted
950	SEBF	12.78	223.08	42.67	16.0	35	21.5	256	1787.2	887.7	0.50	113.7098	26.5091	827	5.2	1.55	0.33	54	1102	Planted
951	SEBF	6.39	114.58	31.02	16.2	33	23.9	236	1331.3	902.4	0.68	112.3046	28.8003	500	5.76	0.54	0.33	20	3475	Primary
952	SEBF	7.73	144.16	46.72	17.0	34	26.6	261	1376.1	927.3	0.67	114.0245	28.2313	360	5.82	1.31	0.44	22	5499	Planted
953	SEBF	9.16	145.95	39.48	13.5	38	20.3	240	1714.5	814.7	0.48	114.1543	28.4841	1040	5.59	1.14	0.42	29	3085	Second
954	SEBF	16.18	252.33	48.43	17.0	34	24.7	239	1682.4	920.3	0.55	114.4053	26.5085	600	5.01	1.22	0.34	31	1680	Primary
955	SEBF	6.29	72.01	21.28	15.2	34	22.1	231	1553.1	870.2	0.56	114.501	28.4096	688	5.53	1.06	0.28	7	3958	Second
956	SEBF	8.66	153.51	33.57	15.6	34	25.4	264	1507.2	883.9	0.59	114.5051	28.4092	601	6.51	1.44	0.55	46	778	Planted
957	SEBF	11.78	204.32	38.31	16.2	37	22.6	304	1448.0	902.1	0.62	114.6033	28.4066	483	5.91	0.56	0.34	43	593	Primary
958	SEBF	13.89	247.02	44.18	16.0	37	22.3	275	1489.9	894.8	0.60	114.7063	28.304	538	6.47	1.41	0.56	47	865	Planted
959	SEBF	5.06	79.22	25.24	14.7	36	20.0	277	1639.0	851.4	0.52	114.7037	28.3038	813	5.66	0.61	0.48	13	4344	Second
960	SEBF	7.59	149.76	64.86	12.8	36	21.5	225	993.9	731.6	0.74	103.4082	28.3021	1900	6.84	1.23	0.42	105	188	Primary
961	SEBF	9.32	176.24	60.11	14.0	33	19.3	236	980.7	781.7	0.80	103.5017	28.8027	1520	6.22	1.43	0.46	53	529	Planted

962	**SEBF**	7.68	143.46	27.92	14.9	37	20.9	231	1224.0	822.0	0.67	106.3961	28.6466	1200	5.79	1.42	0.21	40	827	Planted
963	**SEBF**	9.66	233.38	21.73	14.4	33	21.9	268	800.0	753.4	0.94	97.4047	28.6052	1900	4.9	1.54	0.66	100	1120	Primary
964	**SEBF**	4.60	122.24	48.81	9.2	38	16.4	283	960.4	613.1	0.64	94.902	30.2045	2670	5.41	1.12	0.85	67	289	Primary
965	**SEBF**	4.00	92.86	33.95	9.4	36	17.9	316	636.6	633.1	0.99	85.2053	28.9007	2980	4.86	1.59	1.18	45	1118	Planted
966	**SEBF**	8.04	210.61	85.33	14.0	35	20.8	325	800.0	740.0	0.93	97.0014	28.4065	2020	4.95	1.59	1.18	71	992	Primary
967	**SEBF**	6.39	178.95	68.75	15.2	33	22.7	292	1500.0	800.3	0.53	85.9003	27.9092	2020	6.23	0.86	0.64	50	1025	Planted
968	**SEBF**	7.74	216.4	83.72	9.9	33	15.2	296	1157.3	619.0	0.53	99.3202	27.531	2823	5.03	2.31	1.07	100	1010	Primary
969	**SEBF**	6.01	154.43	54.18	7.3	36	13.4	221	1302.3	523.7	0.40	98.9605	27.9763	3385	6.47	2.2	0.8	100	888	Primary
970	**SEBF**	3.64	338.08	149.45	12.2	35	19.1	257	1171.6	710.9	0.61	101.0073	24.533	2550	5.25	2.3	0.84	100	2000	Primary
971	**SEBF**	5.24	130.17	45.15	12.8	35	19.5	251	1141.5	727.5	0.64	99.681	25.2835	2394	4.84	2.08	0.93	50	1511	Planted
972	**SEBF**	7.31	207.49	80.98	12.4	33	21.7	263	1147.5	712.4	0.62	99.7377	25.2812	2488	5.39	2.27	1.12	60	900	Planted
973	**SEBF**	11.66	306.43	111.00	11.6	36	18.9	256	1150.8	685.0	0.60	99.6867	25.4334	2645	6	2.45	1.2	90	1393	Primary
974	**SEBF**	6.69	172.79	63.70	7.0	36	15.6	304	1378.6	510.2	0.37	98.7891	28.1071	3460	6.75	1.91	0.64	50	2075	Planted
975	**SEBF**	6.76	177.42	63.80	7.4	33	15.6	321	1364.9	527.1	0.39	98.9002	28.1847	3340	6.54	2.44	0.95	150	1034	Primary
976	**SEBF**	5.80	138.59	46.69	3.5	35	8.5	229	1378.5	386.4	0.28	99.0287	28.7586	4160	6.58	2.49	1.02	50	2064	Planted
977	**SEBF**	4.92	128.13	45.79	9.2	34	18.2	282	1125.2	596.0	0.53	99.5636	26.0875	3135	4.96	2.52	1.24	85	942	Primary
978	**SEBF**	5.64	126.2	36.20	12.0	34	21.4	282	1049.1	699.1	0.67	99.6218	26.0871	2480	5.85	1.9	1.39	58	1127	Planted
979	**SEBF**	8.54	189.46	68.20	7.6	34	14.9	325	1019.8	538.3	0.53	100.1082	26.372	3450	5.73	2.28	1.24	85	249	Primary
980	**SEBF**	4.73	117.23	39.88	10.4	36	19.5	311	925.7	639.7	0.69	100.3446	26.525	2783	6.16	2.24	1.53	45	1166	Primary
981	**SEBF**	7.58	175.83	53.68	13.5	34	21.6	322	792.7	754.4	0.95	101.1901	26.4573	2040	5.53	2.4	1.33	85	1586	Primary
982	**SEBF**	8.47	188.21	60.66	10.1	35	16.0	276	1086.4	629.8	0.58	99.57	26.2351	2903	6.05	2.37	1.29	85	474	Primary
983	**SEBF**	4.77	122.88	44.01	9.2	37	16.6	305	1021.2	595.6	0.58	99.8096	26.678	3060	5.98	2	1.22	70	1216	Primary
984	**SEBF**	6.67	161.5	52.99	9.8	37	15.2	308	917.5	618.1	0.67	100.3543	27.0255	2860	5.35	2.57	1.3	120	847	Primary
985	**SEBF**	15.97	359.86	124.73	9.9	38	19.6	316	1094.0	619.4	0.57	99.4413	27.1753	2860	6.3	2.47	1.53	100	631	Primary
986	**SEBF**	7.03	176.69	62.42	10.2	34	18.9	292	1026.4	630.6	0.61	99.7591	27.1789	2780	6.42	2.19	1.59	70	592	Primary
987	**SEBF**	6.77	177.69	66.10	8.1	37	14.1	314	943.4	555.8	0.59	100.2366	27.1736	3240	5.3	2.29	1.48	120	522	Primary
988	**SEBF**	3.58	92.18	33.41	9.6	35	19.4	266	1110.3	609.2	0.55	99.4499	27.3861	2900	6.73	1.99	1.22	85	1021	Primary
989	**SEBF**	5.99	151.51	52.24	7.9	37	15.2	266	876.1	551.6	0.63	100.8939	26.7394	3300	6.89	2.52	1.5	90	867	Primary
990	**SEBF**	5.76	128.23	37.26	9.4	34	18.8	260	870.5	604.1	0.69	100.721	27.1718	2920	6.67	1.97	1.32	100	764	Primary
991	**SEBF**	5.31	133.08	45.40	10.8	36	17.4	320	853.4	655.7	0.77	100.9012	27.324	2570	5.01	2.11	1.28	25	1038	Planted
992	**SEBF**	7.37	168.88	53.55	7.6	37	16.5	236	910.3	538.7	0.59	100.5403	27.6747	3280	5.46	2.21	1.29	20	2799	Planted
993	**SEBF**	8.25	207.42	76.81	10.6	38	19.3	313	1360.3	646.1	0.47	98.7803	25.5838	2900	4.88	2.14	1.46	70	448	Primary
994	**SEBF**	6.26	123.01	29.69	20.0	36	25.7	300	1155.2	992.4	0.86	100.8551	23.21	930	6.51	1.94	1.26	42	2030	Planted
995	**SEBF**	5.09	141.37	53.91	10.6	35	16.1	326	1108.6	648.7	0.59	99.9254	25.5821	2850	5.36	2.28	1.48	50	742	Planted
996	**SEBF**	8.36	215.82	76.14	12.2	35	18.9	309	1233.4	705.0	0.57	99.1468	25.5073	2526	5.03	2.1	1.59	55	1281	Planted
997	**SEBF**	7.38	180.23	59.46	10.9	38	16.4	298	1231.9	657.9	0.53	99.089	25.8088	2790	5.13	2.24	1.34	35	1116	Planted
998	**SEBF**	5.02	126.91	43.72	13.1	35	22.7	251	1066.2	735.3	0.69	99.3862	26.2362	2240	5.98	2.23	1.5	45	713	Planted
999	**SEBF**	4.84	70.59	31.97	15.1	34	21.3	252	1363.2	874.5	0.64	120.1768	30.2559	275	4.96	2.5	1.54	35	1665	Planted
1000	**SEBF**	6.93	89.32	36.77	15.0	38	24.4	277	1490.8	867.4	0.58	119.2775	29.4886	438	6.38	2.39	1.46	35	4584	Planted
1001	**TRMF**	8.75	483.9	80.20	21.9	34	27.4	332	2366.4	1048.2	0.44	109.8224	19.2388	790	5.06	1.39	0.45	110	1127	Primary
1002	**TRMF**	7.80	54.92	26.83	25.2	37	26.5	326	1784.7	1161.3	0.65	110.7388	19.5016	15	5.56	1.13	0.34	20	1967	Planted
1003	**TRMF**	11.63	229.25	171.80	25.0	34	28.2	309	1750.7	1154.6	0.66	110.6217	19.8893	15	4.83	0.48	0.35	55	1100	Planted
1004	**TRMF**	9.83	79.71	26.62	24.1	37	27.6	312	1593.7	1132.4	0.71	109.4004	21.4004	10	5.63	0.67	0.49	20	875	Planted
1005	**TRMF**	7.03	187.3	95.37	24.0	37	28.5	331	1599.2	1128.8	0.71	109.7226	21.4761	15	6.23	0.88	0.26	70	7400	Primary

1006	**TRMF**	5.64	79.73	16.60	20.3	37	29.6	322	2300.0	1031.6	0.45	110.4263	21.6804	585	5.17	0.99	0.35	22	1290	Planted
1007	**TRMF**	22.44	377.88	131.41	23.8	35	29.4	316	1581.0	1124.2	0.71	109.6086	21.7043	20	5.57	1.45	0.58	97	300	Primary
1008	**TRMF**	21.56	185.09	65.50	24.1	37	29.7	343	1542.6	1131.7	0.73	108.3051	21.7032	10	5.6	0.77	0.68	49	1650	Planted
1009	**TRMF**	6.13	84.6	69.26	20.7	34	26.7	358	1574.7	1036.1	0.66	117.9228	24.4085	15	5.07	0.62	0.38	20	20800	Planted
1010	**TRMF**	15.51	425.43	68.72	22.6	39	29.9	343	2205.7	1069.8	0.49	104.6853	18.6214	875	5.71	0.62	0.6	93	1986	Primary
1011	**TRMF**	6.35	253.57	64.67	22.9	38	28.1	328	2292.6	1080.3	0.47	108.8024	18.703	679	6.23	1.53	0.61	49	1007	Planted
1012	**TRMF**	9.58	74.34	24.02	24.0	37	27.8	319	1594.3	1127.9	0.71	109.108	21.5006	35	6.38	0.51	0.22	19	1404	Planted
1013	**TRMF**	8.76	68.3	22.19	23.9	37	29.6	307	1551.2	1124.3	0.72	108.7056	21.8555	28	6.89	1.41	0.39	18	1207	Second
1014	**DRW**	1.40	68.51	18.46	13.7	37	21.5	182	27.6	1297.19	47.00	80.8	38.4013	900	7.97	0.19	0.37	59	700	Planted
1015	**DRW**	0.42	27.92	4.60	11.0	38	20.9	183	44.3	1489.73	33.63	88.1056	40.1027	900	8.24	0.14	0.35	15	550	Second
1016	**DRW**	0.63	42.91	6.63	11.5	36	20.3	165	46.0	1233.25	26.81	80.6061	40.4062	900	8.14	0.37	0.31	68	320	Primary
1017	**DRW**	1.99	50.64	6.60	9.6	35	19.6	193	51.1	1336.63	26.16	85.2009	41.5058	925	8.25	0.39	0.5	56	800	Planted
1018	**DRW**	0.98	67.14	9.60	9.7	35	15.1	179	48.8	1081.39	22.16	82.9055	41.7084	900	7.5	0.22	0.22	25	420	Planted
1019	**DRW**	0.50	24.92	7.57	11.4	34	17.7	172	46.0	1240.47	26.97	80.8092	40.4	900	7.66	0.21	0.53	63	498	Primary
1020	**DRW**	0.66	44.52	8.55	9.3	35	17.4	203	53.2	1289.23	24.23	84.3075	41.7098	950	8.2	0.13	0.68	78	449	Primary
1021	**DRW**	2.29	54.26	27.43	7.9	35	16.5	177	297.2	1248.77	4.20	86.2064	44.3047	650	7.5	0.1	0.45	50	2406	Planted
1022	**DRW**	1.47	43.52	1.48	4.6	34	13.1	200	270.9	1027.6	3.79	86.4068	48.0037	500	7.55	0.18	0.31	42	261	Planted
